# Cholesterol Promotes Lung Adenocarcinoma Brain Metastasis by Stabilizing EGFR Protein to Drive EMT, Metabolic Reprogramming, and Premetastatic Niche Formation

**DOI:** 10.1002/advs.73843

**Published:** 2026-01-21

**Authors:** Ying Chen, Xiaoteng Cui, Xinyi Shi, Xu Yang, Yujing Cao, Haolin LI, Qi Zhan, Qixue Wang, Ang Li, Qihong Cheng, Yunfei Wang, Junhu Zhou, MingJie Wang, Chunsheng Kang, Xiaomin Liu

**Affiliations:** ^1^ Neuro‐Oncology Center Huanhu Hospital Affiliated to Tianjin Medical University Tianjin China; ^2^ Laboratory of Neuro‐oncology Tianjin Neurological Institute Tianjin Medical University General Hospital Tianjin China; ^3^ Neuro‐Oncology Center Huanhu Hospital Affiliated to Tianjin University Tianjin China

**Keywords:** brain metastasis, cholesterol, EGFR, glycolytic reprogramming, lung adenocarcinoma

## Abstract

Brain metastasis is a major cause of mortality in advanced lung adenocarcinoma (LUAD). Accumulating evidence indicates that dysregulated lipid metabolism contributes to metastatic colonization; however, how cholesterol functions as a downstream effector within established lipid‐metabolic programs to regulate key steps of the LUAD brain metastasis (LUAD‐BM) cascade remains incompletely defined. Here, we demonstrate that cholesterol directly engages EGFR and stabilizes its membrane localization by blocking ubiquitin–proteasome–mediated degradation, thereby sustaining AKT/NF‐κB signaling. This signaling axis promotes glycolytic reprogramming and epithelial–mesenchymal transition in LUAD cells, enhancing metastatic capacity and resistance to TKIs. Cholesterol also disrupts blood–brain barrier integrity by reducing endothelial membrane fluidity and accelerating Claudin‐5 ubiquitination and degradation. Within the brain microenvironment, cholesterol directly interacts with IL‐4Rα, facilitating its recruitment into lipid rafts and activation of JAK1/STAT6 signaling, which drives microglial M2 polarization and establishes a permissive pre‐metastatic niche. The cholesterol‐lowering drug atorvastatin reverses these tumor‐intrinsic and microenvironmental effects and suppresses LUAD brain metastasis in vivo. Retrospective clinical analyses further show that hypercholesterolemia is associated with shortened survival in LUAD‐BM patients, whereas statin use correlates with improved outcomes. These findings identify cholesterol as a functional mediator downstream of lipid‐metabolic dysregulation and therapeutic target in LUAD‐BM.

## Graphical abstract

Cholesterol is revealed as a multitasking fuel for lung adenocarcinoma brain metastasis: it locks EGFR at the membrane to sustain AKT/NF‐κB–driven glycolysis and EMT, loosens the blood–brain barrier by promoting Claudin‐5 loss, and rewires microglia through IL‐4R lipid‐raft–JAK1/STAT6 signaling. Atorvastatin counteracts these programs, boosting EGFR‐TKI response and highlighting cholesterol lowering as an actionable strategy.

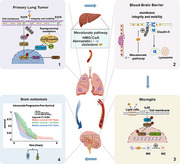

## 1 Introduction

Lung cancer remains one of the most prevalent and lethal malignancies worldwide [1], accounting for a substantial proportion of cancer‐related deaths. The high mortality rate is primarily attributable to distant metastases in advanced stages [2], particularly brain metastases (BMs), which develop in approximately 40%–50% of patients during disease progression [3]. Lung adenocarcinoma (LUAD), the predominant histological subtype of non‐small cell lung cancer (NSCLC) [4], has benefitted from significant therapeutic advances [5], notably through targeted therapies such as tyrosine kinase inhibitors (TKIs) against activating epidermal growth factor receptor (EGFR) mutations [6]. These strategies markedly improve the control of both early and advanced LUAD. However, the clinical efficacy of TKIs is frequently limited by acquired resistance [7], and up to 11% of patients with EGFR‐mutant LUAD eventually develop brain metastases [3]. The prognosis of LUAD patientswith brain metastases (LUAD‐BM) remains poor, with a median survival time ranging from 2 to 27 months and 5‐year survival rates of only 2.4% [8]. These statistics underscore the urgent need to elucidate the molecular mechanisms underlying LUAD‐BM and develop novel therapeutic strategies.

The formation of brain metastases involves a multistep biological cascade [9], including local invasion of the primary tumor, intravasation into the bloodstream, survival of circulating tumor cells (CTCs), extravasation across the blood–brain barrier (BBB), and eventual colonization and proliferation within the brain parenchyma [10, 11]. Each step represents a potential therapeutic target. Unlike metastases to peripheral organs, brain metastasis is uniquely constrained by two major features: (1) the restrictive BBB, which serves as a critical rate‐limiting step in metastatic progression [12], and (2) the distinct immunological milieu of the brain, which exerts selective pressure on disseminated tumor cells and profoundly shapes metastatic seeding and outgrowth [13].

Concurrently, the global obesity epidemic has heightened focus on metabolic dysregulation in cancer biology [14, 15]. Epidemiological studies have linked hypercholesterolemia with adverse outcomes across multiple solid tumors [16, 17, 18]. Cholesterol, beyond serving as a structural lipid, supports membrane organization, lipid raft assembly, and receptor signaling [19, 20], and has been implicated in therapy resistance and metastatic progression [21, 22, 23, 24]. In parallel, emerging work has established that lipid metabolic programs can facilitate brain metastasis, including SREBP1‐driven lipogenesis and broader lipid transport/oxidation adaptations within the brain niche [25, 26, 27].

Together, these studies position lipid metabolism as an established contributor to brain metastasis; however, key questions remain regarding how cholesterol—as a bioactive downstream lipid species within lipid‐metabolic frameworks—interfaces with oncogenic EGFR signaling and the brain microenvironment to coordinate multiple steps of the LUAD‐BM cascade. Specifically, whether cholesterol modulates (1) tumor‐intrinsic programs (EGFR stability, metabolic reprogramming, EMT, and TKI response) and (2) tumor‐extrinsic barriers and niches (BBB integrity and neuroimmune remodeling) in an integrated manner remains insufficiently defined. Addressing these gaps may reveal clinically actionable opportunities to enhance EGFR‐TKI efficacy in LUAD‐BM, particularly in patients with hypercholesterolemia.

In this study, we systematically elucidate the functional contributions of cholesterol across the LUAD‐BM cascade. We demonstrate that cholesterol stabilizes EGFR protein by inhibiting proteasomal degradation and enhancing membrane localization, leading to sustained activation of AKT/NF‐κB signaling, glycolytic metabolic reprogramming, and epithelial–mesenchymal transition (EMT)—collectively facilitating primary tumor progression and invasion. Furthermore, cholesterol disrupts BBB integrity and reprograms microglia toward immunosuppressive M2‐like phenotypes through IL‐4R–JAK1–STAT6 axis activation, thereby establishing a permissive pre‐metastatic niche in the brain. Importantly, we identify atorvastatin (ATO)—a widely used cholesterol‐lowering drug in clinics—as a potent therapeutic agent that reverses these effects, synergizes with EGFR‐TKIs, and improves clinical outcomes in LUAD‐BM patients, as confirmed by retrospective cohort analysis. These findings extend existing lipid metabolism–brain metastasis paradigms by defining cholesterol as a downstream effector that links EGFR signaling, BBB function, and neuroimmune remodeling, supporting statin‐based combination strategies for LUAD‐BM.

## 2 Results

### 2.1 Elevated Level of Serum Cholesterol Promotes Malignant Progression and Brain Metastasis of LUAD In Vivo

**FIGURE 1:**
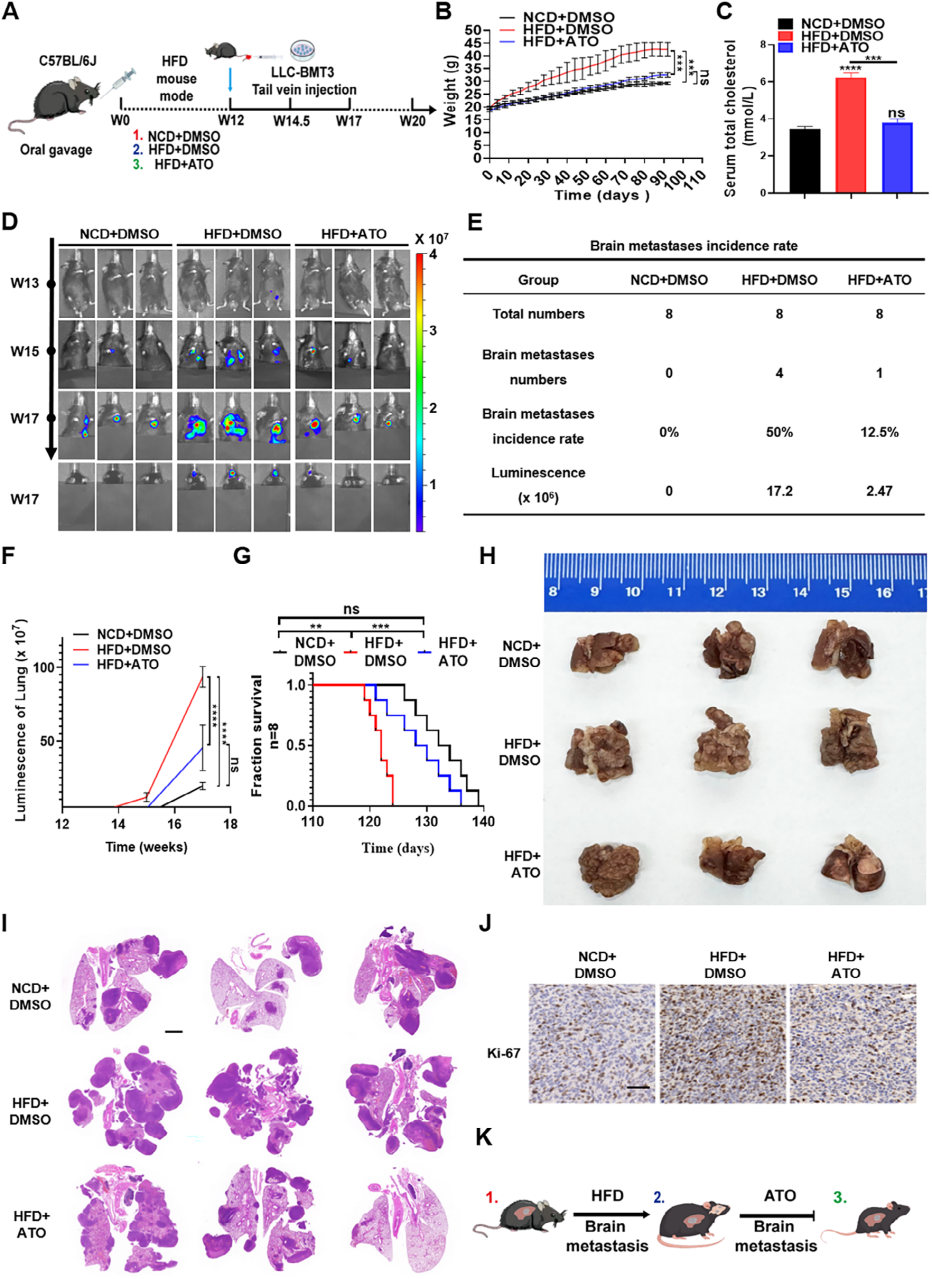
Elevated levels of serum cholesterol promote malignant progression and brain metastasis of LUAD in vivo. A) Schematic diagram illustrating that C57BL/6J male mice (n = 8) received a normal chow diet (NCD), a high‐fat diet (HFD), or HFD with daily oral ATO (10 mg/kg). At week 12, LLC‐BMT3 cells were injected via the tail vein. B‐C) Effects of high‐fat diet on body weight (B) and serum total cholesterol (C). ^***^ *p* < 0.001, ^****^ *p* < 0.0001, ns, not significant (two‐way ANOVA for body weight and one‐way ANOVA for serum total cholesterol). D) Representative bioluminescent imaging of tumor‐bearing mice. E) Tumor growth curves were quantified and illustrated after LLC‐BMT3 cells were injected via the tail vein. F) Tumor growth curves quantified by bioluminescence intensity. ^****^ *p* < 0.0001, ns, not significant (two‐way ANOVA). G) Kaplan–Meier survival curves of mice in different groups. ^**^ *p* < 0.01, ^***^ *p* < 0.001, ns, not significant (log‐rank test). H–J) Representative images (H), HE images (I) of orthotopic tumors presented, and IHC staining of Ki67 (Scale bar = 2 mm for H&E, 60 µm for IHC) (J). K) Schematic illustration of the effect of HFD on promoting LUAD‐BM and the inhibitory role of ATO treatment.

To investigate the role of cholesterol in LUAD‐BM, we first established a brain‐metastatic cell line through in vivo selection. Luciferase‐labeled Lewis lung carcinoma cells (LLC‐Luc) were injected into the carotid artery of C57BL/6J mice. Brain metastases (BrMs) were harvested and re‐injected over three integrative cycles, yielding a brain‐tropic subline named after LLC‐BMT3 with enhanced metastatic potential (Figure S1A,B). To determine the optimal atorvastatin (ATO) dosage for subsequent experiments, we first evaluated three doses (5, 10, and 20 mg/kg/day) via oral gavage in mice. ATO achieved dose‐dependent intracranial exposure: measured brain concentrations were 1.994, 4.361, and 13.987 ng/g at 5, 10, and 20 mg/kg/day, respectively. Notably, the widely used experimental dose of 10 mg/kg/day resulted in readily detectable ATO levels within the central nervous system (CNS)—a concentration that falls within the pharmacologically active range reported in previous preclinical studies(Figure S1C,D). Using a tail vein injection model to simulate lung‐derived hematogenous dissemination, LLC‐BMT3 cells were employed to evaluate systemic metastatic colonization under different cholesterol conditions. Male C57BL/6J mice were then randomized into three treatment groups: normal chow diet (NCD), high‐fat diet (HFD) to induce hypercholesterolemia, and HFD with daily oral atorvastatin administration (ATO, 10 mg/kg) to reduce cholesterol levels. After 12 weeks of dietary intervention, LLC‐BMT3 cells were administered via tail vein injection to model systemic tumor colonization (Figure 1A). We next assessed the metabolic phenotype of mice exposed to a high‐fat diet (HFD): HFD‐fed mice displayed marked increases in body weight, along with elevated serum total cholesterol (TC), LDL‐cholesterol (LDL‐C), and triglycerides (TG), concurrent with reduced HDL‐cholesterol (HDL‐C) — changes validated via serum lipid profiling (HFD: TC = 6.23 ± 0.25 mmol/L, LDL‐C = 0.87 ± 0.06 mmol/L; normal chow diet (NCD) controls: TC = 3.43 ± 0.16 mmol/L, LDL‐C = 0.42 ± 0.06 mmol/L). Hepatic lipid accumulation was also significantly enhanced in HFD‐fed mice, as confirmed by histopathological staining (Figure 1B,C; Figure S2A–D).

Longitudinal bioluminescence imaging (BLI) was used to monitor metastatic progression, revealing that HFD‐fed mice displayed accelerated tumor growth and a marked increase in brain metastatic burden compared with NCD controls. Notably, brain metastasis incidence increased from 0% in the NCD group to approximately 50% in the HFD group, accompanied by a substantial elevation in intracranial luminescence intensity (Figure 1D–F; Figure S3A). Consistent with these findings, Kaplan–Meier survival analysis revealed significantly shortened overall survival in the HFD group (*p* < 0.01, Figure 1G). To further evaluate systemic dissemination, we assessed extracranial organs: while HFD modestly increased metastatic lesions in the lungs, liver, kidneys, and bones, the magnitude of this enhancement was far weaker than that observed in the brain (Figure S3A–C). Histopathological examination confirmed these observations. Notably, ATO reduced these extracranial metastatic events, but its most pronounced therapeutic effect was consistently observed in the brain. Additionally, hematoxylin and eosin (H&E) staining of lung tissues from HFD mice revealed a higher number and density of metastatic nodules, frequently exhibiting the characteristic millet‐like morphology (Figure 1H,I). Immunohistochemical (IHC) analysis of Ki67 expression further showed that cholesterol treatment elevated the proliferative capacity of lung tumors in situ (Figure 1J). In contrast, ATO‐treated mice exhibited attenuated tumor progression, reduced brain metastasis incidence, and prolonged survival—supporting the efficacy of cholesterol‐lowering therapy in suppressing metastatic progression (Figure 1K).

Collectively, using a tail vein–based systemic dissemination model in immunocompetent mice, these findings demonstrate that elevated hypercholesterolemia promotes in situ malignant progression and preferentially drives brain metastatic colonization of LUAD cells, while ATO‐mediated cholesterol reduction reverses this phenotype.

### 2.2 Cholesterol Increases the Membrane‐Localized Levels of EGFR in LUAD

Epidermal growth factor receptor (EGFR) mutations occur in approximately 10%–35% of LUAD [28], with significantly higher frequencies (up to 66.3%) observed in non‐smoking Asian populations. At initial diagnosis, up to 29% of patients with EGFR mutations present with brain metastases [29]. To assess whether EGFR expression is associated with brain‐metastatic potential, we compared EGFR levels between the parental LLC cells and the brain‐seeking LLC‐BMT3 subline. Western blotting result revealed elevated EGFR expression in LLC‐BMT3 cells (Figure S4A), suggesting an association between EGFR protein expression levels and LUAD‐BM.

**FIGURE 2:**
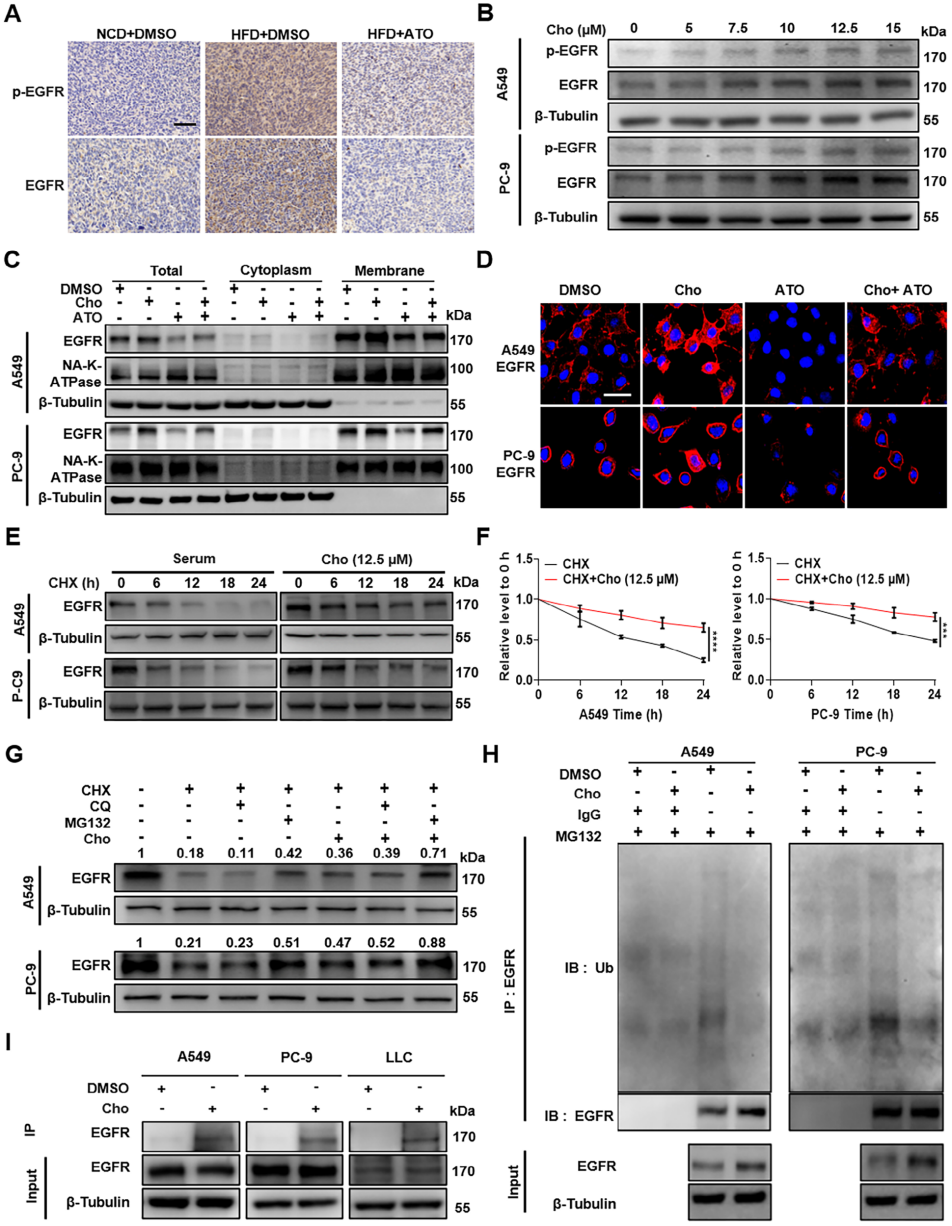
Cholesterol increases the membrane‐localized levels of EGFR in LUAD. A) Representative IHC staining of total EGFR and p‐EGFR in tumor tissues from C57BL/6J male mice fed with NCD, HFD, or HFD with ATO treatment. (scale bar, 100 µm). B) A549 and PC‐9 cells were treated with increasing concentrations of cholesterol (Cho) (0, 5, 7.5, 10, 12.5, and 15 µmol/L) for 48 h. Protein levels of p‐EGFR, EGFR, and β‐Tubulin were examined by western blotting. C) Cytosolic and membrane protein fractions were isolated from A549 and PC‐9 cells treated with DMSO, Cho (12.5 µmol/L), ATO (10 µmol/L), or the combination of ATO and Cho. Membrane‐associated EGFR and p‐EGFR levels were detected via western blot, with Na‐K‐ATPase and β‐Tubulin used as membrane and cytosolic references, respectively. D) Immunofluorescence staining of EGFR in A549 and PC‐9 cells under the same treatment conditions (scale bar, 60 µm). E,F) EGFR degradation was assessed in A549 and PC‐9 cells treated with CHX (10 µg/mL) alone or in combination with 12.5 µmol/L Cho for 48 h. Protein levels were measured by western blotting (E), and band intensities were quantified (F). ^****^ *p* < 0.0001 (two‐way ANOVA). G) Western blot analysis of EGFR following treatment with CHX (10 µg/mL), MG132 (10 µm), CQ (25 µm), or Cho (12.5 µmol/L) for 48 h. H) Co‐immunoprecipitation of EGFR from A549 and PC‐9 cells treated with DMSO or Cho (12.5 µmol/L) for 48 h, followed by immunoblotting for assessing ubiquitinated EGFR level. I) Co‐immunoprecipitation of EGFR from A549 and PC‐9 cells treated with DMSO or cholesterol‐binding chemical probe for 24 h, followed by immunoblotting for assessing ubiquitinated EGFR level.

To determine whether cholesterol regulates EGFR expression during LUAD progression, we analyzed tumor tissues from mice administrated with different dietary regimens. IHC staining revealed significantly higher levels of both total EGFR and phosphorylated EGFR (p‐EGFR) in tumors from HFD mice compared to NCD controls. These elevations were markedly attenuated by ATO treatment (Figure 2A).

Then, we treated LUAD cell lines A549, PC‐9, and LLC with increasing concentrations of cholesterol. Both total EGFR and p‐EGFR protein levels were upregulated in dose‐ and time‐dependent manners (Figure 2B; Figure S4B–I). Considering the role of cholesterol in regulating membrane dynamics [30], we subsequently analyzed EGFR subcellular distribution. Cellular fractionation of cytosolic and membrane compartments revealed pronounced enrichment of membrane‐localized EGFR and p‐EGFR upon cholesterol treatment (Figure 2C; Figure S5A,C–E). These findings were confirmed by immunofluorescence (IF) staining, which demonstrated enhanced EGFR membrane localization in cholesterol‐treated cells (Figure 2D; Figure S5B).

We next explored whether cholesterol affects EGFR protein turnover. Cycloheximide (CHX) chase assays showed that cholesterol treatment significantly prolonged the half‐life of EGFR protein, indicating suppressed degradation (Figure 2E–F; Figure S6A,B). Since autophagy and the ubiquitin‐proteasome system (UPS) represent major protein degradation pathways, we tried to distinguish which one is more important for EGFR degradation. A548, PC‐9, and LLC cells were treated with lysosome inhibitor Chloroquine (CQ), proteasome inhibitor MG132, CHX, or Cholesterol (Cho) for 24 h, respectively. CHX monotreatment induced substantial EGFR degradation, which was rescued by MG132 but not by CQ co‐treatment. Compared to CHX treatment alone, combinatorial treatment of Cho and CHX slowed down the degradation of EGFR protein. This cholesterol‐mediated stabilization of EGFR was reversed by MG132 (Figure 2G; Figure S6C). Furthermore, co‐immunoprecipitation analysis demonstrated that cholesterol treatment could reduce EGFR ubiquitination (Figure 2H; Figure S6D), consistent with proteasomal degradation blockade. To further underlying molecular mechanism by which cholesterol affects the ubiquitination‐mediated degradation of membrane‐localized EGFR, we performed co‐immunoprecipitation (co‐IP) assays using a cholesterol‐binding chemical probe. Consistent with prior evidence that cholesterol can directly bind and modulate target proteins, including membrane receptors [31], our co‐IP results demonstrated a direct physical interaction between cholesterol and EGFR in LUAD cells (Figure 2I). This interaction led to a marked reduction in EGFR ubiquitination levels, thereby impairing ubiquitin‐mediated proteasomal degradation of membrane‐localized EGFR. These data provide a direct molecular explanation for why cholesterol stabilizes EGFR and facilitates its persistent membrane retention.

Collectively, these findings reveal that cholesterol enhances LUAD brain‐metastatic potential, at least in part, by directly binding to EGFR, reducing its ubiquitination, stabilizing membrane‐localized EGFR, and sustaining downstream oncogenic signaling, including AKT/NF‐κB activation and metabolic reprogramming.

### 2.3 Cholesterol Promotes EMT and Malignant Progression in LUAD via Activating EGFR/AKT/NF‐κB/β‐Catenin Signaling

**FIGURE 3:**
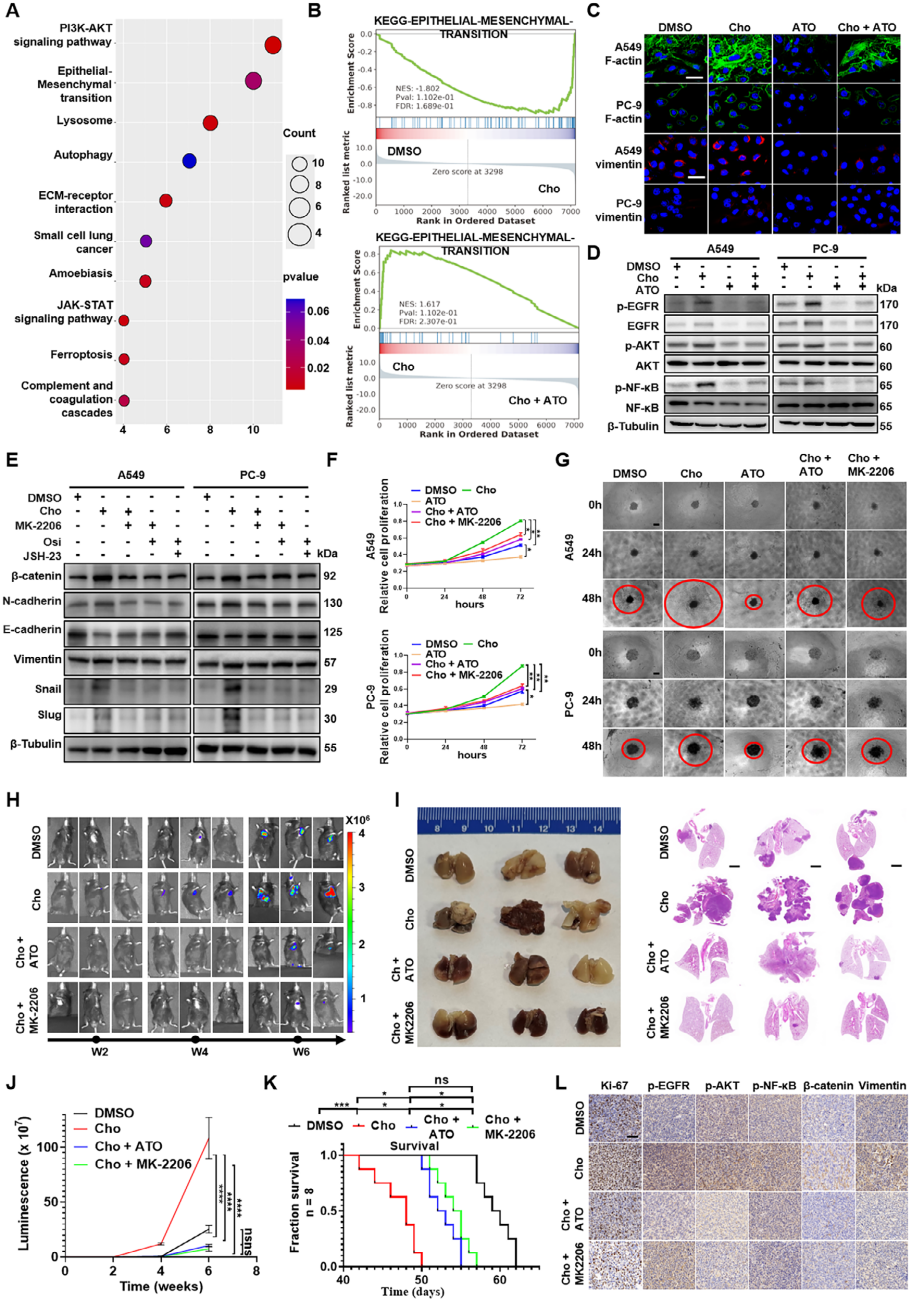
Cholesterol promotes EMT and tumor progression in LUAD via activating EGFR/AKT/NF‐κB/β‐catenin signaling. A) PC‐9 cells were treated with DMSO, Cho (12.5 µmol/L) or ATO (10 µmol/L) plus Cho (12.5 µmol/L), and then subjected to MS‐based proteomic analysis (n = 3 biological replicates for each group). KEGG pathway analysis shows the top 10 enriched pathways, including the PI3K‐AKT signaling and EMT. B) Gene set enrichment analysis of the EMT. NES (normalized enrichment score) and p‐value are shown. C) Immunofluorescence staining for F‐actin and Vimentin in A549 and PC‐9 cells treated with DMSO, Cho, ATO, or ATO plus Cho (scale bar, 60 µm). D) Western blot of p‐EGFR, EGFR, p‐AKT, AKT, p‐NF‐κB, NF‐κB, and β‐Tubulin in A549 and PC‐9 cells treated with DMSO, Cho (12.5 µmol/L), ATO (10 µmol/L), or ATO plus Cho. E) Western blot analysis of EMT markers in A549 and PC‐9 cells treated with DMSO, Cho (12.5 µmol/L), Osi, MK‐2206, or JSH‐23. F, G) Growth curves (F) and brightfield images of 3D tumor sphere invasion assays (G) for A549 and PC‐9 cells treated with DMSO, Cho, Osi, MK‐2206 (scale bar, 300 µm). ^*^ *p* < 0.05, ^**^ *p* < 0.01, ^***^ *p* < 0.001, ^****^ *p* < 0.0001 (two‐way ANOVA for growth curves and one‐way ANOVA for 3D tumor sphere invasion assays). H, I) In vivo metastasis model: LLC‐BMT3 cells treated with DMSO, Cho, ATO plus Cho, or MK‐2206 plus Cho were injected via tail vein into C57BL/6J male mice. Representative bioluminescent imaging of tumor‐bearing mice (H). Representative images, HE images of orthotopic tumors presented (I). J) Tumor growth curves were quantified and illustrated in each treatment group following intravenous injection of LLC‐BMT3 cells. ^****^ *p* < 0.0001, ns, not significant (two‐way ANOVA). K) Kaplan‐Meier curves for different experimental and control groups. ^*^ *p* < 0.05, ^***^ *p* < 0.001, ns, not significant (log‐rank test). L) IHC staining of Ki67, p‐EGFR, p‐AKT, p‐NF‐κB, PGK1, LDHA, ALDOA, β‐catenin, and Vimentin (Scale bar, 60 µm).

EMT is a hallmark of cancer progression, conferring enhanced migratory and invasive capabilities to tumor cells [32]. To explore the EMT impact of cholesterol on LUAD‐BM, we performed mass spectrometry‐based proteomic profiling of PC‐9 cells treated with DMSO (control), cholesterol, or cholesterol in combination with ATO. KEGG pathway enrichment of the proteomics dataset revealed significant upregulation of EMT‐related proteins following cholesterol treatment (Figure 3A), further confirmed by gene set enrichment analysis (Figure 3B). Among the 7408 differentially expressed proteins identified, 201 were upregulated in the cholesterol‐treated group and downregulated upon ATO treatment, while 773 proteins exhibited the opposite pattern (Figure S7A). RT‐qPCR analysis demonstrated dose‐ and time‐ dependent induction of EMT‐associated transcription factors Snail1 and Snail2 (Slug) in A549, PC‐9, and LLC cells upon cholesterol stimulation (Figure S7B–D).

Given the critical roles of cytoskeletal remodeling during EMT, we evaluated F‐actin and vimentin expression [33]. Cholesterol treatment markedly enhanced both F‐actin polymerization and vimentin expression, as visualized by immunofluorescence (Figure 3C; Figure S8A). Vimentin, a key intermediate filament protein, is known to facilitate cytoskeletal stability, cell motility, and EMT induction [34]. Correspondingly, western blot analysis revealed increased expression levels of mesenchymal markers (Vimentin, N‐cadherin, β‐catenin, Snail, Slug) with concomitant reduction of epithelial marker E‐cadherin, indicating a shift from epithelial toward mesenchymal phenotype (Figure S8B).

To elucidate the upstream regulatory mechanism, KEGG analysis of proteomics data revealed significant enrichment of differentially expressed proteins in the PI3K–AKT signaling (Figure 3A), corroborated by gene set enrichment analysis (Figure S8C). Western blotting showed that cholesterol activated the EGFR/AKT/NF‐κB signaling, evidenced by increased phosphorylation levels of EGFR, AKT, and NF‐κB (Figure 3D; Figure S8D).

To explore the upstream mechanism, we treated cells with EGFR inhibitor Osimertinib, AKT inhibitor MK‐2206, or NF‐κB inhibitor JSH‐23. These inhibitors attenuated cholesterol‐induced expression of EMT markers, demonstrating that EGFR/AKT/NF‐κB signaling is required for cholesterol‐driven EMT (Figure 3E; Figure S8E, F). To establish causality, we conducted gene‐perturbation and rescue experiments. EGFR knockdown abrogated cholesterol‐induced AKT/NF‐κB activation and EMT marker upregulation, whereas expression of constitutively active myr‐AKT restored EMT signaling even under EGFR depletion or ATO treatment (Figure S9A,B), confirming that AKT acts downstream of EGFR and is sufficient to drive cholesterol‐mediated EMT.

Functionally, cholesterol promoted tumorigenic behaviors in LUAD cells. By employing in vitro assays, cholesterol enhanced cell proliferation, 3D spheroid invasion, wound closure, and transwell migration in A549, PC‐9, and LLC cells. These pro‐invasive effects were reversed by ATO or MK‐2206 (Figure 3F,G; Figure S10A–E). To evaluate whether cholesterol‐induced tumor‐intrinsic alterations facilitate metastatic dissemination from the primary tumor site, LLC‐BMT3 cells were pre‐treated in vitro with DMSO, cholesterol (Cho), cholesterol plus ATO, or cholesterol plus MK‐2206, and subsequently injected via the tail vein into male C57BL/6J mice. This tail vein–based model was used to simulate hematogenous dissemination of tumor cells originating from the primary lung tumor and to assess systemic metastatic competence, including secondary brain colonization (Figure S11A). Mice in the cholesterol‐treated group exhibited significantly increased lung colonization and metastatic foci compared to those in the DMSO control group, while ATO or MK‐2206 treatment reversed Cho‐induced effects (Figure 3H–J). Kaplan–Meier survival curves showed a marked reduction in survival in the cholesterol group (p < 0.001), whereas ATO or MK‐2206 treatment prolonged survival (Figure 3K). Immunohistochemical analysis of tumor tissues revealed hyperactivation of the EGFR/AKT/NF‐κB signaling axis, accompanied by upregulation of EMT markers in the cholesterol group (Figure 3L), providing in vivo validation of the mechanistic findings observed in vitro. Consistent with a model of primary tumor–derived systemic dissemination, a modest increase in metastatic burden was also observed in extracranial organs (Figure S11B,C).

Collectively, using a tail vein injection model designed to reflect metastatic dissemination from the primary tumor, these findings demonstrate that cholesterol promotes LUAD invasiveness and metastatic progression by inducing EMT through the EGFR/AKT/NF‐κB cascade both in vitro and in vivo.

### 2.4 Cholesterol Reshapes the Energy Metabolism of LUAD Cells by Regulating the EGFR/AKT/NF‐κB Signaling

**FIGURE 4:**
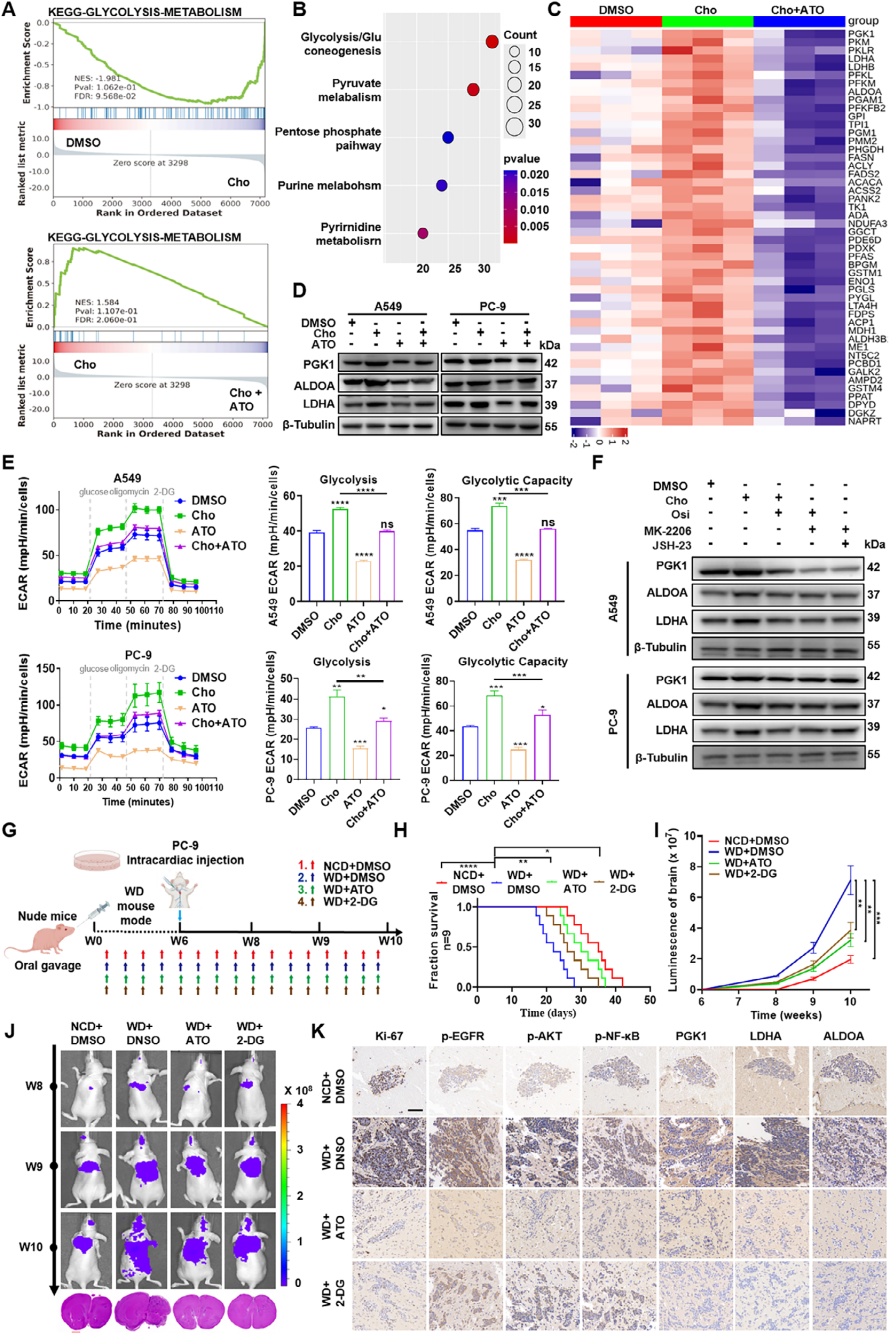
Cholesterol reshapes the energy metabolism of LUAD cells by regulating the EGFR/AKT/NF‐κB signaling. A) PC‐9 cells were treated with DMSO, 12.5 µmol/L Cho or ATO plus 12.5 µmol/L Cho, followed by mass spectrometry‐based proteomic analysis (n = 3 biological replicates per group). Gene set enrichment analysis (GSEA) identified significant enrichment in glycolysis‐related pathways. NES (normalized enrichment score) and p‐values are shown. B) KEGG pathway enrichment of altered metabolic pathways from proteomics data. C) Heatmap showing differentially expressed proteins across treatment groups, highlighting metabolic enzymes. D) Western blot of PGK1, LDHA, ALDOA, and β‐Tubulin in A549 and PC‐9 cells treated with DMSO, 12.5 µmol/L Cho, ATO, or ATO plus 12.5 µmol/L Cho. E) Glycolytic function was assessed by Seahorse XF Glycolytic Rate Assay in A549 and PC‐9 cells treated as above for 48 h. ECAR (extracellular acidification rate), glycolysis, and glycolytic capacity were quantified. ^*^ *p* < 0.05, ^***^ *p* < 0.001, ^****^ *p* < 0.0001 (one‐way ANOVA). F) Western blot of p‐EGFR, EGFR, p‐AKT, AKT, p‐NF‐κB, NF‐κB, and β‐Tubulin in A549 and PC‐9 cells treated with DMSO, 12.5 µmol/L Cho, Osimertinib (EGFR inhibitor), MK‐2206 (AKT inhibitor) or JSH‐23 (NF‐κB inhibitor). G) Schematic diagram illustrating that BALB/C nude mice, male mice (n = 9) received NCD, WD, WD with daily oral ATO (10 mg/kg) or WD with daily oral 2‐DG (400 mg/kg). At week 6, PC‐9 cells were administered via intracardiac injection. H) Kaplan‐Meier curves for different experimental and control groups. ^*^ *p* < 0.05, ^**^ *p* < 0.01, ^****^ *p* < 0.0001 (log‐rank test). I) Tumor growth curves were quantified and illustrated. ^**^ *p* < 0.01, ^***^ *p* < 0.001 (two‐way ANOVA). J) Representative bioluminescent imaging of tumor‐bearing mice and HE images of orthotopic tumors are presented. K) IHC staining of Ki67, p‐EGFR, p‐AKT, p‐NF‐κB, PGK1, LDHA, and ALDOA (Scale bar, 60 µm).

Emerging evidence suggests that glycolytic reprogramming is particularly critical for supporting tumor cell survival and outgrowth in the brain microenvironment, prompting us to investigate whether cholesterol‐driven metabolic shifts contribute to enhanced brain metastatic colonization [35]. The mass spectrometry‐based proteomic result showed cholesterol treatment significantly enriched glycolysis‐related pathways compared to the control group, as revealed by KEGG pathway enrichment analysis and gene set enrichment analysis (Figure 4A,B). Heatmap plot highlighted broad upregulation of metabolism‐associated proteins in cholesterol‐treated cells and reversal by statin co‐treatment. Notably, glycolytic enzymes including ALDOA, PGK1, LDHA, and PFKM were among the most prominently regulated targets (Figure 4C). Western blotting result further confirmed cholesterol stimulation upregulated key glycolytic enzymes—PGK1, LDHA, and ALDOA—in A549, PC‐9, and LLC cells. These effects were effectively reversed by ATO, while PFKM expression remained unchanged (Figure 4D; Figure S12A,B). Functional analysis using Seahorse XF Glycolytic Rate Assay revealed that cholesterol significantly enhanced glycolytic activity, as indicated by elevated extracellular acidification rate (ECAR), glycolytic flux, glycolytic capacity, and glycolytic reserve. These enhancements were abolished by ATO treatment (Figure 4E; Figure S13A–C).

Pharmacological inhibition of EGFR, AKT, or NF‐κB reduced cholesterol‐induced upregulation of PGK1, LDHA, and ALDOA (Figure 4F; Figure S14A). Moreover, Seahorse assays showed that blockade of this signaling cascade also attenuated glycolytic function (Figure S14B–D), suggesting that cholesterol‐mediated metabolic reprogramming is orchestrated through the EGFR/AKT/NF‐κB signaling. To further establish the causal relationship between EGFR–AKT signaling and cholesterol‐driven metabolic reprogramming, we performed gene perturbation and rescue experiments. EGFR knockdown (shEGFR‐1# and shEGFR‐2#) markedly reduced cholesterol‐induced upregulation of key glycolytic enzymes (PGK1, LDHA, ALDOA), whereas reactivation of AKT through myr‐AKT expression restored their protein levels despite EGFR depletion or ATO treatment (Figure S15A,B). Consistently, Seahorse assays demonstrated that EGFR silencing significantly impaired cholesterol‐enhanced glycolytic flux and capacity, while myr‐AKT expression reinstated these metabolic phenotypes (Figure S15C,D). To more directly interrogate brain‐specific metastatic seeding and outgrowth in an EGFR‐mutant context, we established an intracardiac brain metastasis model using PC‐9 cells in Western diet (WD)–fed mice. In this setting, we deliberately employed a Western diet containing 1.25% cholesterol to more directly and robustly accentuate the biological effects of cholesterol itself, rather than generalized high‐fat intake, thereby enabling clearer attribution of brain metastatic phenotypes to hypercholesterolemia. The intracardiac injection strategy bypasses primary tumor growth and pulmonary trapping, allowing direct assessment of brain‐tropic metastatic potential under cholesterol‐enriched systemic conditions. To further investigate the contribution of metabolic reprogramming to cholesterol‐driven brain metastasis, in vivo glycolytic dependency was concurrently evaluated (Figure 3G). Serum biochemical analysis confirmed that Western diet (WD)‐fed mice exhibited significantly elevated serum total cholesterol (TC) and LDL‐cholesterol (LDL‐C) levels, coupled with reduced HDL‐cholesterol (HDL‐C), compared to normal chow diet (NCD)‐fed controls (WD: TC = 5.27 ± 0.25 mmol/L, LDL‐C = 0.57 ± 0.15 mmol/L; NCD: TC = 3.00 ± 0.20 mmol/L, LDL‐C = 0.93 ± 0.15 mmol/L) (Figure S16A–D). WD feeding markedly accelerated intracranial tumor growth, increased brain metastatic burden, and significantly shortened survival compared with NCD controls, whereas ATO and the glycolysis inhibitor 2‐DG effectively suppressed brain colonization and prolonged survival (Figure 4G–J). IHC analyses confirmed that WD‐fed mice exhibited elevated Ki67, p‐EGFR, and glycolytic enzyme expression—changes that were reversed by ATO treatment (K). In addition, although WD modestly increased metastases in extracranial organs, the magnitude of this increase was far less pronounced than that observed in the brain. Both ATO and 2‐DG significantly reduced metastatic lesion numbers across multiple organs, with the strongest therapeutic effect consistently occurring in the brain (Figure S16E,F).

Collectively, using an intracardiac EGFR‐mutant LUAD brain metastasis model combined with a cholesterol‐enriched Western diet specifically designed to emphasize cholesterol‐driven effects, these results collectively demonstrate that cholesterol promotes glycolytic reprogramming in LUAD cells by activating EGFR/AKT/NF‐κB signaling, thereby enhancing the metabolic flexibility required for metastatic progression.

### 2.5 Cholesterol Reduced Cerebral Endothelial Cell Membrane Fluidity and Enhanced Claudin‐5 Ubiquitination, Disrupting BBB Integrity

Our above findings demonstrated that cholesterol promotes LUAD growth and invasion by inducing glycolytic reprogramming and EMT. However, successful metastasis requires a multistep cascade beyond primary tumor invasion, including intravasation into the circulation, survival as circulating tumor cells, extravasation at distant organs, and colonization of the metastatic niche [9]. Interestingly, we observed that the brain metastasis rate in mice under systemic hypercholesterolemia was significantly higher than that in mice injected with cholesterol‐preconditioned LUAD cells (Figure 1E; Figure S11B). These findings suggest that cholesterol may facilitate not only tumor cell aggressiveness in the primary lesion but also their extravasation, colonization, and proliferation in the brain parenchyma.

**FIGURE 5:**
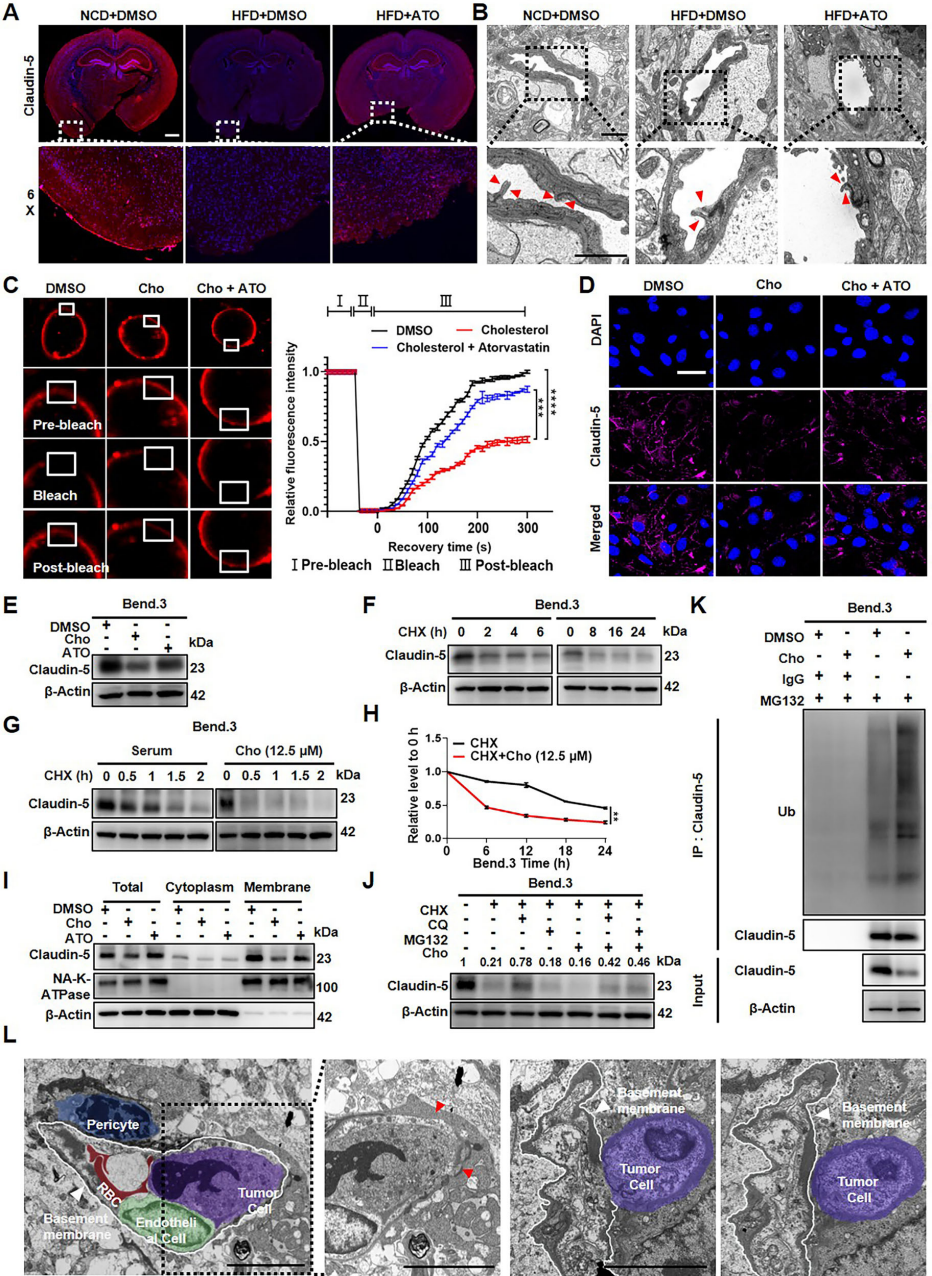
Cholesterol reduced cerebral endothelial cell membrane fluidity and enhanced Claudin‐5 ubiquitination, disrupting BBB integrity. A) Immunofluorescence staining of brain sections from C57BL/6J male mice fed NCD, HFD or HFD were orally administered ATO (10 mg/kg/day) for 12 weeks. Claudin‐5 (pink) expression was used as a marker to evaluate BBB integrity in each group (Scale bar, 1 mm). B) Transmission electron microscopy (TEM) images of the BBB structure in each group. Red arrowheads indicate tight junctions between cerebral endothelial cells (Scale bar, 2 µm and 1.5 µm). C) FRAP assays in Bend.3 cells treated with DMSO, Cho (12.5 µmol/L), or ATO (10 µmol/L) plus Cho for 48 h (scale bar, 10 µm). D‐E) Immunofluorescence assays (D) and western blotting (E) to test the expression levels of Claudin‐5 (pink) in Bend.3 cells treated with DMSO, 12.5 µmol/L Cho (12.5 µmol/L), or ATO (10 µmol/L) plus Cho (scale bar, 60 µm). F) Western blot analysis of Claudin‐5 degradation in Bend.3 cells treated with CHX (10 µg/mL). G,H) Western blot analysis (G) and densitometric quantification (H) of Claudin‐5 in Bend.3 cells treated with CHX alone or CHX plus Cho for 48 h. ^**^ *p* < 0.01 (two‐way ANOVA). I) The components of cytosol and membrane in Bend.3 cells treated with DMSO, Cho (12.5 µmol/L), or ATO (10 µmol/L) plus Cho were fractionated. The distribution levels of Claudin‐5, Na‐K‐ATPase, and β‐Tubulin were measured by western blotting and quantification of the protein expression. J) Western blotting analysis of Claudin‐5 in Bend.3 cells treated with CHX (10 µg/mL), MG132 (10 µm), CQ (25µm), or Cho (12.5 µm) for 48 h, respectively. K) Co‐IP assay using anti–Claudin‐5 antibody followed by immunoblotting with anti‐ubiquitin in Bend.3 cells treated with DMSO or Cho. L) TEM images of tumor cells infiltration through a cholesterol‐compromised BBB. Pericytes (blue), red blood cells (RBCs, red), endothelial cells (green), tumor cells (purple), and basement membrane (white) are highlighted (Scale bars, 5 µm).

The blood–brain barrier (BBB), primarily sealed by endothelial tight junction proteins such as Claudin‐5, Occludin, and ZO‐1 [12]. This structure contributes to the restricted brain metastasis incidence compared to liver or bone [36]. To evaluate cholesterol‐induced blood–brain barrier (BBB) dysfunction in vivo, mice were subjected to a 12‐week dietary intervention with an NCD, HFD, or HFD+ATO, a regimen that induces a stable state of systemic hypercholesterolemia (Figure 1C; Figure S2A–D). Evans Blue tracer assays subsequently revealed extensive dye extravasation in HFD‐fed mice, which was markedly reduced by ATO treatment (Figure S17A, B), directly demonstrating increased BBB permeability under hypercholesterolemic conditions. To determine the most responsive tight junction component, we compared Claudin‐5, Occludin, and ZO‐1 expression. Among them, Claudin‐5 exhibited the most pronounced reduction in HFD mice and the strongest restoration after ATO treatment, whereas Occludin and ZO‐1 showed minimal or no detectable changes (Figure 5A; Figure S17C). This provided a clear rationale for focusing on Claudin‐5 as the key cholesterol‐responsive BBB regulator in our study. Transmission electron microscopy (TEM) revealed disrupted tight junction structures between adjacent endothelial cells in the HFD group, further confirming BBB damage (Figure 5B).

We next investigated whether cholesterol directly regulates the biophysical properties of endothelial cell membranes, using Bend.3 (murine brain microvascular endothelial) cells in an in vitro model. In Bend.3 cells, fluorescence recovery after photobleaching (FRAP) assays revealed that cholesterol reduced membrane fluidity (Figure 5C). Immunofluorescence and western blot analysis further confirmed cholesterol‐induced downregulation of Claudin‐5 (Figure 5D,E). Mechanistically, cycloheximide (CHX) chase assays revealed that cholesterol significantly shortened the half‐life of Claudin‐5 protein, indicating accelerated turnover (Figure 5F,G). Moreover, co‐treatment with the proteasome inhibitor MG132, but not the lysosomal inhibitor chloroquine, rescued cholesterol‐induced Claudin‐5 downregulation, indicating proteasome‐dependent degradation (Figure 5H–J). Co‐immunoprecipitation assays confirmed enhanced ubiquitinated modification of Claudin‐5 upon cholesterol treatment (Figure 5K), supporting a mechanism by which cholesterol promotes Claudin‐5 degradation via the ubiquitin–proteasome system. To visualize the functional consequence of this BBB disruption, we used TEM to track the extravasation process of LUAD cells into the brain parenchyma (Figure 5L): HFD‐induced Claudin‐5 reduction and tight junction disruption created “leakage sites” in the endothelial barrier, allowing tumor cells to traverse the BBB and infiltrate the brain parenchyma. To directly test whether reinforcing BBB tight junctions can counteract cholesterol‐induced dysfunction, we overexpressed Claudin‐5 in endothelial cells. Claudin‐5 overexpression successfully restored its protein levels under cholesterol treatment (Figure S18A). Due to technical limitations in visualizing dynamic BBB permeability in vivo, we next employed an in vitro BBB co‐culture model to quantify functional consequences. GFP‐labeled LUAD cells were added to the endothelial monolayer, and trans‐endothelial migration was assessed. Cholesterol markedly increased tumor cell transmigration, whereas Claudin‐5 overexpression significantly reduced the number of GFP^+^ cells crossing the BBB layer, effectively reversing cholesterol‐induced barrier breakdown (Figure S18B).

Collectively, in vivo dietary models establish BBB leakage and tight junction disruption under systemic hypercholesterolemia, in vitro bEnd.3 assays define the biophysical and ubiquitin–proteasome mechanism driving Claudin‐5 loss, and the BBB co‐culture system functionally links Claudin‐5 restoration to reduced tumor cell transmigration. These findings demonstrate that cholesterol impairs BBB integrity by reducing membrane fluidity and promoting the ubiquitination and degradation of Claudin‐5, thereby facilitating LUAD cell entry into the brain and promoting metastatic colonization.

### 2.6 Cholesterol Promoted the M2 Polarization of Microglia by Facilitating the Binding of the IL‐4 Receptor to Lipid Rafts and Activating the JAK1/STAT6 Signaling

Microglia, the resident macrophages of the brain, are critical regulators of the neural immune microenvironment [37], maintaining central nervous system (CNS) homeostasis and mediating innate immune responses [38]. Previous studies have shown that microglia, particularly those polarized toward M2‐like phenotypes, support the malignant progression of primary and metastatic brain tumors [39].

**FIGURE 6:**
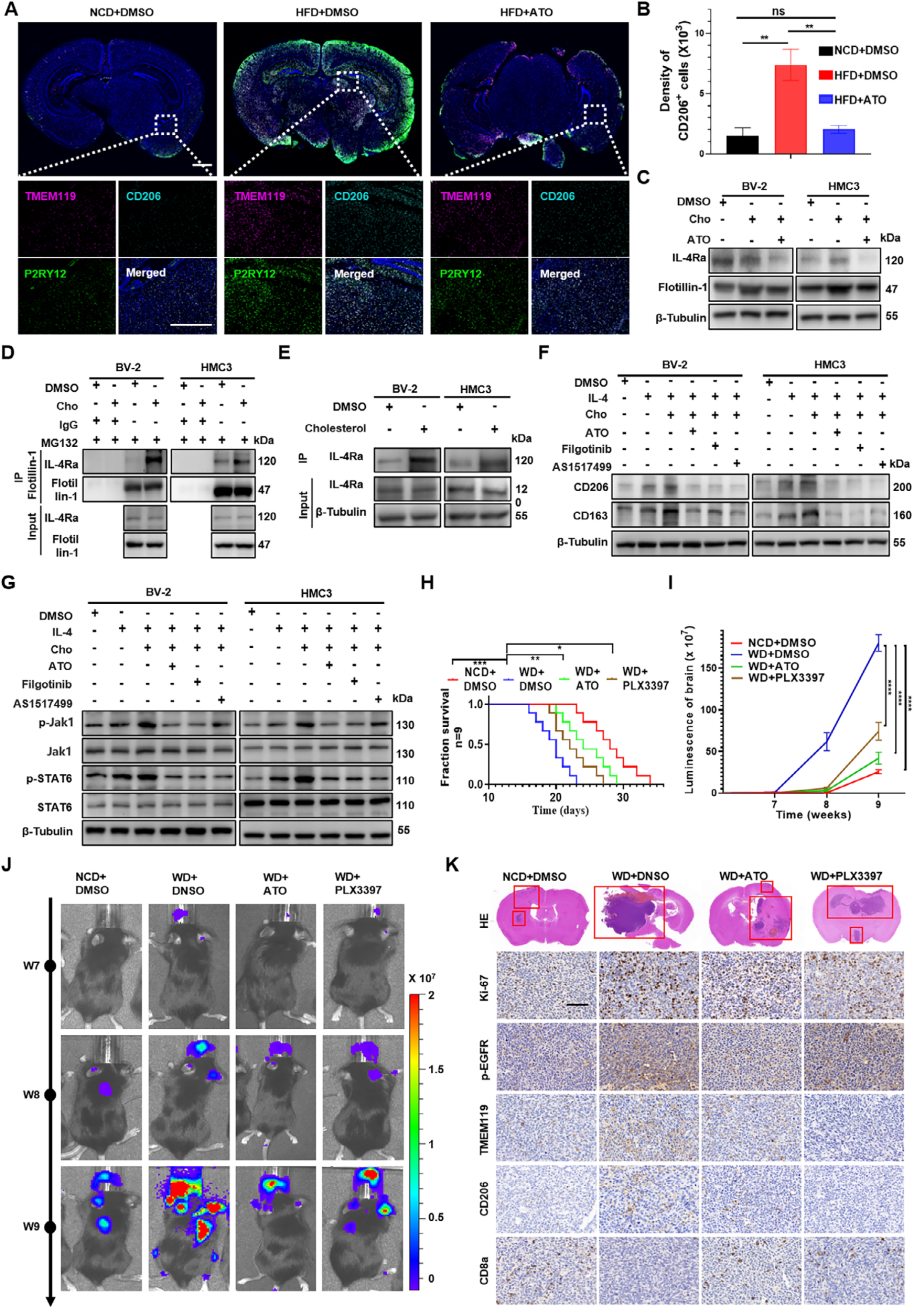
Cholesterol promoted the M2 polarization of microglia by facilitating the binding of the IL‐4 receptor to lipid rafts and activating JAK1/STAT6 signaling. A‐B) Brain sections were obtained from C57BL/6J male mice fed an NCD, HFD, or HFD with oral ATO (10 mg/kg/day) for 12 weeks. Expression levels of TMEM119/P2RY12 (pink/green, microglia marker) and CD206 (cyan, M2 marker) were assessed by immunofluorescence staining (scale bar, 1 mm). The CD206^+^ positive cells in each microscope field were counted. ^**^ *p* < 0.01, ns, not significant (one‐way ANOVA). C) Western blot analysis to assess the expression and localization of IL‐4Rα and lipid raft marker Flotillin‐1 in BV‐2 and HMC3 cells treated with DMSO, Cho (12.5 µmol/L), or Cho plus ATO (10 µmol/L). D) Co‐IP assays using anti–Flotillin‐1 antibody in BV‐2 and HMC3 cells treated with DMSO or Cho (12.5 µmol/L) for 48 h, followed by immunoblotting with IL‐4Rα. E) Co‐IP of IL‐4R from BV‐2 and HMC3 cells treated with DMSO or cholesterol‐binding chemical probe for 24 h, followed by immunoblotting for assessing ubiquitinated IL‐4Rα level. F, G) BV‐2 and HMC3 cells treated with IL‐4 (20 ng/ml), DMSO, Cho (12.5 µmol/L), ATO (10 µmol/L), Filgotinib (1 µm), or AS1517499 (0.1 µm). Western blot analysis of CD206 and CD163 expression. β‐Tubulin served as the loading control (F). Western blot analysis of JAK1/STAT6 pathway activation. The expression levels of total and phosphorylated JAK1 (p‐JAK1), STAT6 (p‐STAT6), and β‐Tubulin were evaluated (G). C57BL/6J male mice (n = 9) received NCD, WD, WD with daily oral ATO (10 mg/kg) or WD with daily PLX3397 (40 mg/kg). At week 6, LLC‐BMT3 cells were administered via intracardiac injection. H‐L) Kaplan‐Meier curves for different experimental and control groups. ^*^ *p* < 0.05, ^**^ *p* < 0.01, ^***^ *p* < 0.001 (log‐rank test) (H). Tumor growth curves were quantified and illustrated. ^****^ *p* < 0.0001 (two‐way ANOVA) (I). Representative bioluminescent imaging of tumor‐bearing mice (J). H&E images of orthotopic tumors presented and IHC staining of Ki67, p‐EGFR, TMEM119, CD206 and CD8a (Scale bar, 60 µm) (K).

To determine whether cholesterol contributes to microglial polarization in vivo, brain tissues were collected from mice after 12 weeks of dietary intervention with an NCD, HFD, or HFD+ATO. This long‐term feeding regimen induces a stable systemic hypercholesterolemic state (Figure 1C; Figure S2A–D), and brain sections were subsequently analyzed by immunofluorescence staining. The result showed that CD206^+^ microglia were markedly increased in the HFD group compared with the NCD group, indicative of M2 polarization, while ATO administration attenuated this effect (Figure 6A,B). Meanwhile, we found that cholesterol treatment of murine BV‐2 and human HMC3 microglial cell lines similarly elevated expression of M2 markers CD206 and CD163 at both the protein and transcript levels in vitro (Figure S19A,B).

Interleukin‐4 (IL‐4), a key Th2‐type cytokine, drives M2 microglial polarization via the IL‐4 receptor (IL‐4R) [40]. Lipid rafts, which are cholesterol‐ and sphingolipid‐rich microdomains within the plasma membrane, serve as critical platforms for receptor clustering and signaling transduction [41]. By using immunofluorescence and co‐immunoprecipitation assays, we found that cholesterol enhanced the co‐localization and physical interaction between IL‐4Rα and the lipid raft marker Flotillin‐1, suggesting that cholesterol facilitates IL‐4R recruitment into lipid raft domains (Figure 6C,D; Figure S19C). Importantly, co‐IP experiments using cholesterol probes further demonstrated that cholesterol directly binds to IL‐4Rα, providing mechanistic evidence that cholesterol promotes IL‐4Rα raft localization through direct receptor engagement (Figure 6E).

The JAK/STAT signaling plays important roles in TAM activation [42]. Downstream of IL‐4R, JAK1/STAT6 signaling is essential for expressions of M2‐type genes in tumor‐associated macrophages (TAMs) and microglia. We observed that cholesterol stimulation significantly increased phosphorylated levels of JAK1 and STAT6, consistent with pathway activation. Pharmacological inhibition of JAK1 or STAT6 by inhibitor Filgotinib or AS1517499 antagonized the cholesterol‐induced M2 polarization and JAK1/STAT6 signaling activation (Figure 6F,G). To further validate pathway dependency, we supplemented STAT6 knockdown (siSTAT6‐1# and siSTAT6‐2#) experiments, which similarly abolished cholesterol‐driven M2 marker expression, confirming that microglial reprogramming requires JAK1/STAT6 signaling (Figure S20A, B).

To further assess how hypercholesterolemia shapes the pre‐metastatic brain niche, we performed immunofluorescence staining of brain tissues to examine lipid raft abundance (Flotillin‐1), M2 microglia (CD206), and BBB integrity (Claudin‐5) across the NCD+DMSO, HFD+DMSO, and HFD+ATO groups. HFD‐fed mice showed markedly increased Flotillin‐1^+^ lipid raft accumulation and elevated CD206^+^ M2 microglia, accompanied by a pronounced reduction in Claudin‐5 expression. Notably, ATO treatment effectively reversed all of these changes (Figure S21A). These results demonstrate that hypercholesterolemia actively remodels the pre‐metastatic brain microenvironment by enhancing lipid‐raft signaling platforms, promoting immunosuppressive M2 microglial expansion, and compromising BBB integrity. To functionally interrogate the contribution of microglia to cholesterol‐driven brain metastasis in vivo, we employed a Western diet–based hypercholesterolemic brain metastasis model combined with pharmacological microglial depletion. Male BALB/C nude mice were maintained on a cholesterol‐enriched Western diet to establish systemic hypercholesterolemia, followed by intracardiac injection of luciferase‐labeled LLC‐BMT3 cells at week 6, to initiate brain metastatic seeding. Microglial depletion was subsequently achieved by administration of the CSF1R inhibitor PLX3397, allowing selective ablation of brain‐resident microglia during metastatic outgrowth (Figure S21B). PLX3397 treatment eliminated brain‐resident microglia, abolished the cholesterol‐associated increase in CD206^+^ M2 cells, and significantly attenuated brain metastatic outgrowth. Notably, mice in the WD group exhibited markedly increased infiltration of M2‐like microglia together with reduced CD8^+^ T‐cell presence, whereas ATO treatment effectively reversed these alterations, indicating that cholesterol‐lowering therapy restores antitumor immune surveillance (Figure 6H–K; Figure S21C,D). Given the critical role of microglia in regulating local immune activity, we further investigated whether cholesterol‐activated microglia directly modulate CD8^+^ T‐cell function. Purified CD8^+^ T cells (Figure S22A) were co‐cultured for 72 h with BV‐2 microglia that had been pre‐treated with DMSO, Cho, or Cho plus ATO for 48h. Flow cytometry analysis revealed that cholesterol‐treated microglia markedly increased PD‐1 expression on CD8^+^ T cells, whereas ATO co‐treatment reversed this effect (Figure S22B,C), demonstrating that cholesterol‐polarized microglia directly induce CD8^+^ T‐cell suppression.

While the IL‐4/JAK1/STAT6 signaling axis is known to regulate microglial M2 polarization, our study identifies a novel upstream mechanism: cholesterol directly interacts with IL‐4R and facilitates the recruitment of IL‐4Rα into lipid rafts, enhancing activation of JAK1/STAT6 signaling—thus amplifying M2 polarization in the context of brain metastasis. Collectively, in vivo dietary models establish microglial M2 enrichment within the brain under systemic hypercholesterolemia, in vitro microglial assays define the IL‐4R–lipid raft–JAK1/STAT6 mechanism, and the WD intracardiac model with PLX3397 depletion provides functional evidence that microglia are required for cholesterol‐driven brain metastatic outgrowth. This microglia‐dependent immunosuppressive reprogramming of the neural microenvironment likely contributes to the cholesterol‐driven promotion of LUAD brain metastasis.

### 2.7 Cholesterol Promotes the Formation of a Pre‐metastatic Niche in the Brain, Facilitating Micro Colonization and Proliferation of LUAD‐BM

Crosstalk between tumor cells and the brain microenvironment critically regulates metastatic colonization [43]. Disseminated tumor cells often undergo mesenchymal‐to‐epithelial transition (MET) at metastatic sites, which is a process enhancing colonization efficiency in lung cancer brain metastases [44]. We therefore hypothesized that cholesterol‐conditioned microglia facilitate MET in mesenchymal‐like LUAD cells to promote metastatic outgrowth.

**FIGURE 7:**
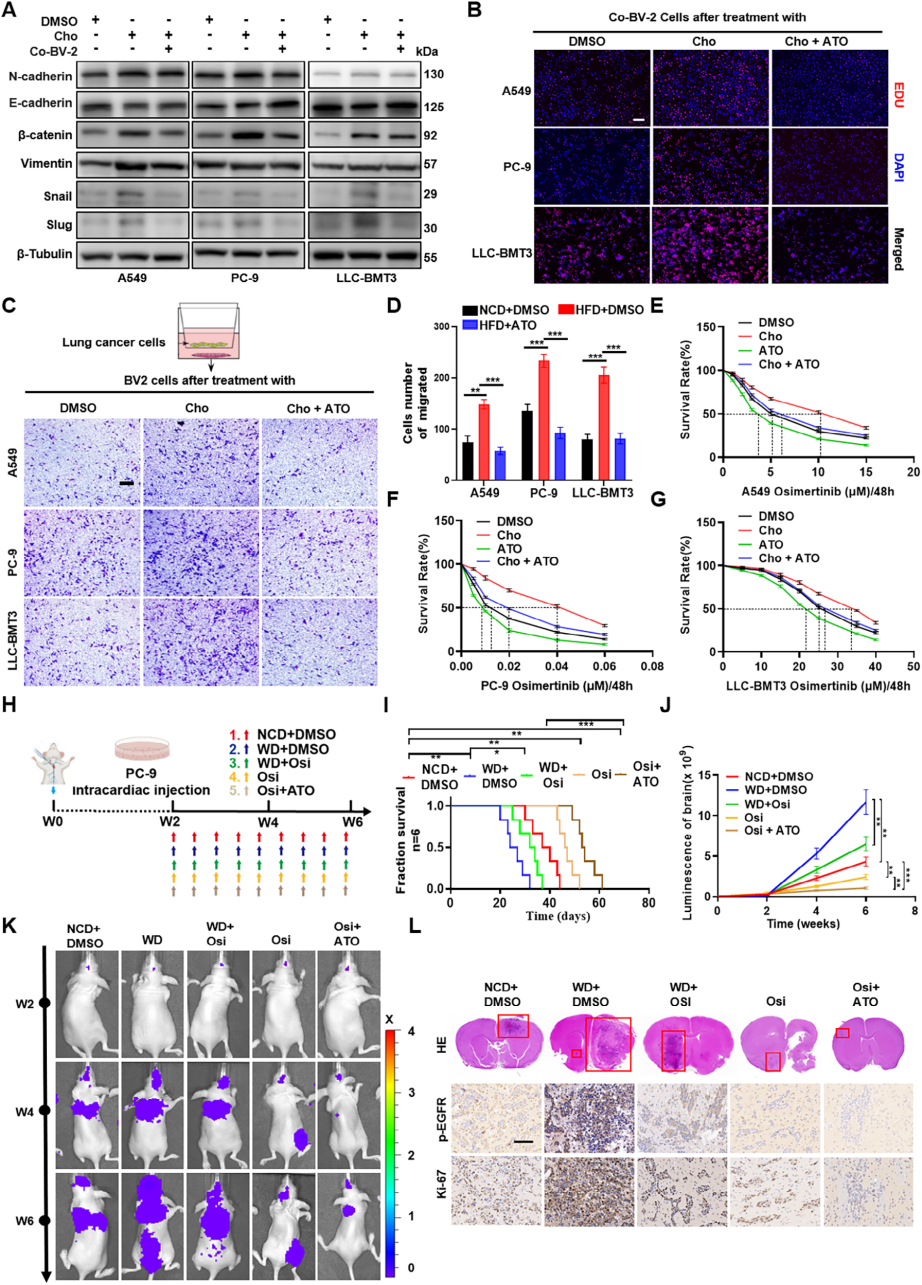
Cholesterol promotes the formation of a pre‐metastatic niche in the brain, facilitating micro colonization and proliferation of LUAD‐BM. A) Western blot analysis of EMT‐related protein expression (β‐catenin, N‐cadherin, E‐cadherin, Snail, Slug, Vimentin, and β‐Tubulin) in A549, PC‐9, and LLC‐BMT3 cells treated with DMSO, Cho, or supernatant from BV‐2 cells. B‐D) BV‐2 microglial cells were treated with DMSO, Cho (12.5 µmol/L), ATO (10 µmol/L), or ATO plus Cho for 48 h, after which the treatment media were completely replaced with fresh standard medium. The cells were then cultured for an additional 48 h before the supernatants were collected. These conditioned media were subsequently applied to A549, PC‐9, and LLC‐BMT3 cells for 48 h. Fluorescence images of EdU assays were obtained using the confocal microscope (scale bar, 500 µm) (B). BV‐2 microglia (pre‐treated with DMSO, 12.5 µmol/L Cho, or Cho plus10 µmol/L ATO for 48 h) were seeded in the lower chamber, and tumor cells in the upper chamber. Migrated onto the lower surface of the transwell chamber were stained with crystal violet. Representative images are shown (scale bar, 300 µm) (C), and data are presented ^**^ *p* < 0.01, ^***^ *p* < 0.001 (one‐way ANOVA) (D). E–G) Survival rates of osi‐treated A549 (E), PC‐9 (F), and LLC‐BMT3 (G) were measured after pre‐exposure to DMSO, Cho (12.5 µmol/L), ATO (10 µmol/L) or ATO plus Cho for 48 h. H) Schematic diagram illustrating that male BALB/C nude mice were initially maintained on a normal chow diet. Luciferase‐labeled PC‐9 cells were administered via intracardiac injection, and two weeks later—after confirmation of established brain metastases by bioluminescence imaging—mice were randomized and subsequently assigned to receive NCD or WD, together with DMSO, Osi, or Osi+ATO treatment (n = 6 per group). I‐L) Kaplan‐Meier curves for different experimental and control groups. ^**^ *p* < 0.01, ^***^ *p* < 0.001 (log‐rank test) (I). Tumor growth curves were quantified and illustrated. ^**^ *p* < 0.01, ^***^ *p* < 0.001 (two‐way ANOVA) (J). Representative bioluminescent imaging of tumor‐bearing mice (K). H&E images of orthotopic tumors presented, and IHC staining of Ki67 and p‐EGFR (Scale bar, 60 µm) (L).

To test this, we collected conditioned media from BV‐2 microglia pre‐treated with DMSO, Cho, or Cho+ATO. Importantly, to avoid direct confounding by cholesterol, BV‐2 cells were first exposed to these treatments for 48 h, after which the media were completely replaced with fresh standard medium and cells were cultured for an additional 48 h before supernatants were collected. Western blotting revealed that supernatant from cholesterol‐treated microglia upregulated MET‐associated markers in A549, PC‐9 and LLC‐BMT3 cells (Figure 7A; Figure S23A–C), indicating EMT reversal. Furthermore, EdU proliferation assays showed that conditioned media from cholesterol‐treated BV‐2 microglial cells enhanced LUAD cells proliferation compared to control media (Figure 7B). We further assessed whether cholesterol‐activated microglia enhance LUAD cell invasiveness using a Transwell co‐culture system, in which BV‐2 microglia (pre‐treated with DMSO, cholesterol, or cholesterol + ATO for 48 h) were seeded in the lower chamber and tumor cells in the upper chamber. Cholesterol markedly increased LUAD cell migration across the membrane, whereas ATO effectively reversed this effect (Figure 7C,D), supporting the notion that cholesterol‐driven microglial activation promotes tumor cell motility and colonization potential.

Brain metastases in LUAD patients pose significant therapeutic challenges due to acquired resistance to EGFR tyrosine kinase inhibitors (EGFR‐TKIs). Given the role of cholesterol in EGFR stabilization and immune modulation, we hypothesized that a hypercholesterolemic microenvironment contributes to TKI resistance in LUAD‐BM. Pharmacological cholesterol reduction using ATO combined with osimertinib leads to a significant decrease in proliferation of EGFR‐TKI‐resistant cells compared to osimertinib monotherapy, and the effect of ATO was counteracted by cholesterol replenishment (Figure 7E–G). To more rigorously evaluate whether hypercholesterolemia affects EGFR‐TKI therapeutic response during established brain metastasis, we employed an intracardiac PC‐9 LUAD‐BM model. Two weeks after cell injection—when brain metastases were already detectable—we performed BLI screening and selected mice that had successfully developed intracranial lesions of comparable tumor burden. These animals were then randomly assigned to receive NCD+DMSO, WD+DMSO, WD+Osi, Osi alone, or combined Osi+ATO treatment (n = 6 per group) (Figure 7H). Under Western diet conditions, intracranial tumors progressed rapidly despite osimertinib treatment, demonstrating that hypercholesterolemia markedly compromises EGFR‐TKI efficacy. Notably, combined Osi+ATO treatment produced the strongest antitumor effect, yielding substantially lower intracranial luminescence signals and significantly prolonged survival relative to Osi alone (Figure 7I–K). Histopathological analysis further supported these findings: brain metastatic lesions from WD‐fed mice displayed higher Ki67 and p‐EGFR staining, reflecting increased proliferative activity and enhanced EGFR pathway activation. In contrast, tumors from the Osi+ATO group exhibited markedly reduced Ki67 and p‐EGFR levels (Figure 7L), demonstrating effective suppression of tumor proliferation and EGFR signaling.

Collectively, in vitro microglia‐conditioned medium and co‐culture systems indicate that cholesterol‐activated microglia promote MET‐associated phenotypic switching, proliferation, and motility of LUAD cells, whereas the in vivo EGFR‐mutant intracardiac PC‐9 model demonstrates that dietary hypercholesterolemia compromises osimertinib efficacy and that atorvastatin co‐treatment restores therapeutic control. These findings support the rationale for cholesterol‐lowering strategies as an adjunct to EGFR‐TKIs, particularly in hypercholesterolemic settings.

### 2.8 Elevated Serum Cholesterol Levels Promote the Initiation and Progression of LUAD‐BM

**FIGURE 8:**
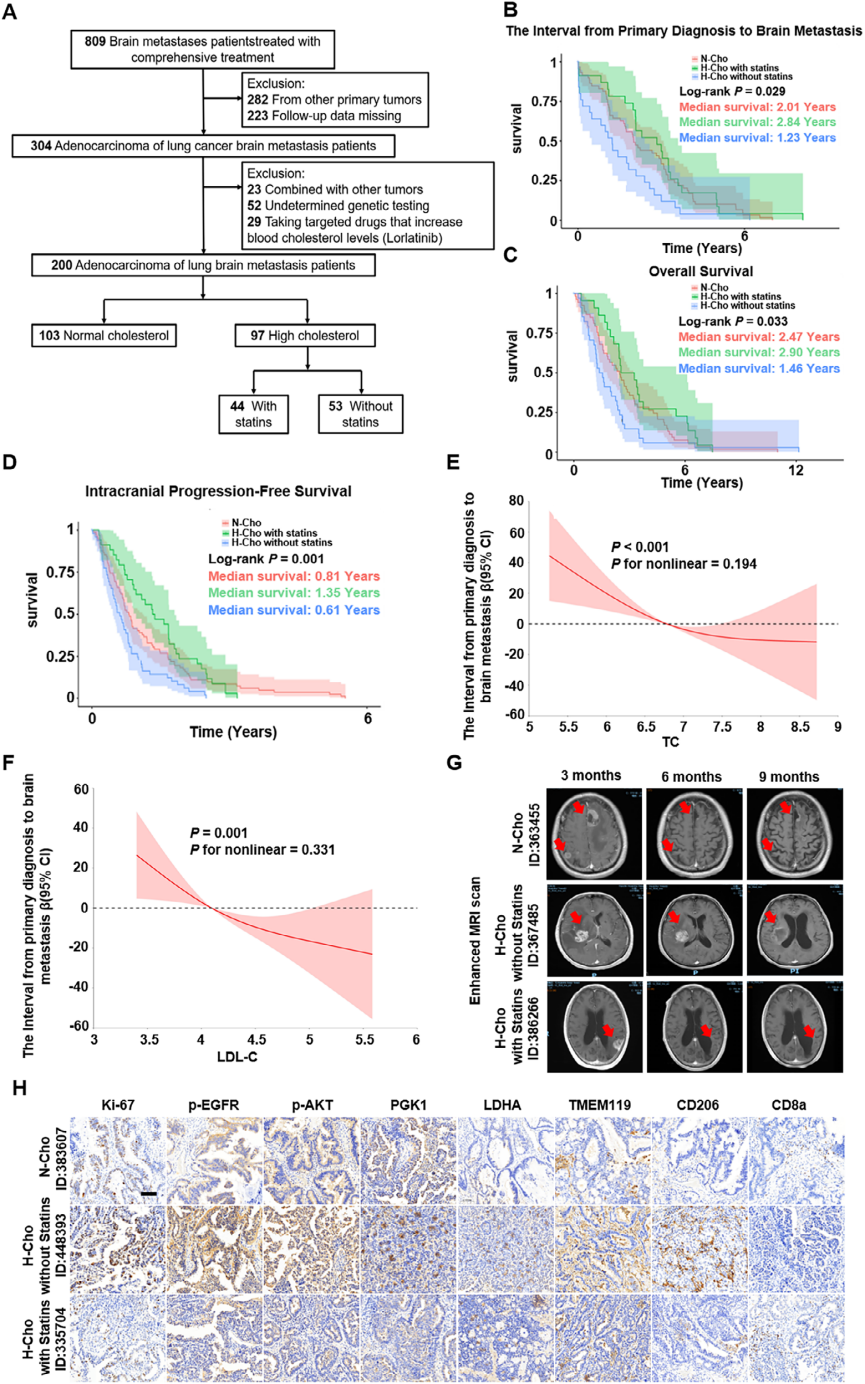
Elevated serum cholesterol levels promote the initiation and progression of LUAD‐BM. A) Flow diagram illustrating patient selection for the retrospective cohort study. B‐D) Kaplan‐Meier curve of the metastasis‐free interval (B), OS (C), and IPFS (C) in LUAD‐BM patients (log‐rank test). E,F) Restricted Cubic Splines for the association of the Interval from metastatic risk with TC (E) and LDL‐C (F). G) Representative intracranial MRI images of LUAD‐BM cases from different patient groups during post‐treatment follow‐up. H) Representative images of FFPE sections of primary tumor tissues in different groups for IHC staining of Ki67, p‐EGFR, p‐AKT, PGK1, LDHA, Iba1, CD206, and CD8a (Scale bar, 60 µm).

To validate the clinical relevance of cholesterol‐mediated EGFR‐TKI resistance in LUAD‐BM, we retrospectively collected patients from Huanhu Hospital Affiliated to Tianjin Medical University during January 2022 to December 2024. Among 809 screened LUAD‐BM patients, 200 patients who had complete clinical records and baseline fasting lipid profiles obtained at the time of lung adenocarcinoma diagnosis were included in the final analysis. These patients were stratified according to baseline serum cholesterol status into three groups: normal cholesterol (N‐Cho, n = 103), hypercholesterolemia with statin treatment (H‐Cho+Statins, n = 44), and hypercholesterolemia without statin treatment (H‐Cho, n = 53) (Figure 8A; Table 1 and Table S1).

**TABLE 1:** Clinical information of a total of 200 LUAD‐BM patients from Huanhu Hospital Affiliated to Tianjin Medical University.

	Full population			
	N‐Cho (N=103)	H‐Cho without Statins (N=53)	H‐Cho with Statins (N=44)	*P*
**Age** ^a^	61.24 (10.06)	62.19 (10.28)	65.00 (8.77)	0.108
**BMI** ^a^	23.87 (2.45)	24.09 (3.06)	23.98 (2.78)	0.889
**ECOG score** ^a^	3.23 (1.84)	3.81 (1.66)	3.55 (1.69)	0.144
**Line of therapy** ^a^	1.43 (0.71)	1.45 (0.77)	1.77 (0.99)	0.047
**Gender** ^b^				
Male	59 (57.3)	23 (43.4)	27 (61.4)	0.150
Female	44 (42.7)	30 (56.6)	17 (38.6)	
**Mutation types** ^b^				
no positive target mutation	43 (41.7)	21 (39.6)	12 (27.3)	0.173
EGFR	47 (45.6)	26 (49.1)	26 (59.1)	
ALK	3 (2.9)	1 (1.9)	4 (9.1)	
ROS1	5 (4.9)	1 (1.9)	0 (0.0)	
KRAS	2 (1.9)	0 (0.0)	0 (0.0)	
MET	0 (0.0)	0 (0.0)	2 (3.8)	
HER2	0 (0.0)	1 (1.9)	1 (2.3)	
RET	1 (1.0)	0 (0.0)	0 (0.0)	
ERBB3	0 (0.0)	1 (1.9)	0 (0.0)	
SMARCA4	1 (1.0)	0 (0.0)	0 (0.0)	
TP53	0 (0.0)	0 (0.0)	1 (2.3)	
BRAF V600E	1 (1.0)	0 (0.0)	0 (0.0)	
**Diabetes** ^b^				
No	89 (86.4)	47 (88.7)	39 (88.6)	0.891
Yes	14 (13.6)	6 (11.3)	5 (11.4)	
**Cardiovascular disease** ^b^				
No	75 (72.8)	38 (71.7)	27 (61.4)	0.363
Yes	28 (27.2)	15 (28.3)	17 (38.6)	
**Hyperlipidemia** ^b^				
No	103 (100.0)	0 (0.0)	0 (0.0)	<0.001
Yes	0 (0.0)	53 (100.0)	44 (100.0)	
**Statin use** ^b^				
No	103 (100.0)	53 (100.0)	0 (0.0)	<0.001
Atorvastatin	0 (0.0)	0 (0.0)	38 (86.4)	
Rosuvastatin	0 (0.0)	0 (0.0)	5 (11.4)	
Lovastatin	0 (0.0)	0 (0.0)	1 (2.3)	
**Steroid use** ^b^				
No	2 (1.9)	1 (1.9)	0 (0.0)	0.651
Yes	101 (98.1)	52 (98.1)	44 (100.0)	
**TKI treatment** ^b^				
No	45 (43.7)	20 (37.3)	12 (27.3)	0.171
Yes	58 (56.3)	33 (62.3)	32 (72.7)	
**Metastasis Count** ^b^				
<3	56 (54.4)	20 (37.7)	14 (31.8)	0.020
>3 or Meningeal Metastasis	47 (45.6)	33 (62.3)	30 (68.2)	
**Smoking history** ^b^				
No	73 (70.9)	35 (66.0)	30 (68.2)	0.819
Yes	30 (29.1)	18 (34.0)	14 (31.8)	
**Alcohol use** ^b^				
No	88 (85.4)	43 (81.1)	39 (88.6)	0.579
Yes	15 (14.6)	10 (18.9)	5 (11.4)	
**Surgical intervention** ^b^				
No	79 (76.7)	45 (84.9)	39 (88.6)	0.177
Yes	24 (23.3)	8 (15.1)	5 (11.4)	

^a^ Mean (Standard Deviation), *P* values from the ANOVA test.

^b^ N (%), *P* values from the χ^2^ test.

Comprehensive clinical information—including demographic variables (age, sex), lifestyle factors (smoking and alcohol use), comorbidities (hypertension, diabetes, cardiovascular disease), EGFR mutation status, diagnostic and treatment history, and baseline fasting serum lipid levels (TC and LDL‐C)—was collected and systematically analyzed. Baseline demographic and clinical characteristics were well balanced across groups and are summarized in Table 1.

Survival analysis revealed that LUAD‐BM patients with statins administration exhibited significantly prolonged time from primary diagnosis to brain metastasis (termed “metastasis‐free interval”), as well as longer overall survival (OS) and intracranial progression‐free survival (IPFS), compared to those not receiving statins (Figure 8B–D). These findings suggest a beneficial effect of cholesterol‐lowering treatment on delaying metastatic progression and enhancing EGFR‐TKI efficacy. Restricted cubic spline (RCS) regression analysis was performed to quantitatively assess the relationship between lipid levels and metastatic risk, OS, and IPFS. Elevated TC and LDL‐C levels were significantly associated with increased risk of brain metastasis (TC: p < 0.001; LDL‐C: p = 0.001), OS (TC: p = 0.002; LDL‐C: p = 0.013), and IPFS (TC: p = 0.005; LDL‐C: p = 0.007). The metastasis‐free intervals were decreased in LUAD‐BM patients with higher TC and LDL‐C levels (Figure 8E, F; Figure S24A–D).

To further determine whether hypercholesterolemia itself represents an independent prognostic factor, we additionally performed univariate and multivariate Cox proportional‐hazards regression analyses, adjusting for potential confounders including hypercholesterolemia status, statin use, age, gender, mutation types, BMI, diabetes, cardiovascular disease, line of therapy, TKI treatment, smoking history, alcohol use, surgical intervention, and metastasis Count. The results showed that hypercholesterolemia remained a significant independent predictor of shortened metastasis‐free interval and poor survival outcomes, whereas statin administration independently conferred a protective survival benefit. Specifically, multivariate models demonstrated that hypercholesterolemic patients without statins had significantly higher metastatic risk (HR = 2.01, 95% CI: 1.16–3.48), whereas statin use was independently associated with improved OS (HR = 2.20, 95% CI = 1.29‐3.74, p = 0.004) and IPFS (HR = 1.75, 95% CI = 1.17‐2.62, p = 0.006). (Table S5–S7).

Representative MRI scans further illustrated these clinical trends. Among EGFR‐mutant LUAD patients receiving standard first‐line EGFR‐TKI–based combination therapy, those with normal serum cholesterol consistently achieved favorable responses, with one representative case showing partial response (PR) at 6 months and complete remission (CR) at 9 months. In contrast, patients with hypercholesterolemia but without statin treatment demonstrated delayed and incomplete responses, exemplified by a case reaching only PR at 9 months despite comparable therapy. Strikingly, hypercholesterolemic patients receiving statin co‐treatment exhibited markedly improved therapeutic outcomes, with one representative case achieving CR within just 3 months (Figure 8G), suggesting that cholesterol lowering substantially enhances EGFR‐TKI responsiveness. To strengthen these clinical observations, we expanded the imaging dataset to include an additional 4 independent patients per group (N‐Cho, H‐Cho, H‐Cho+Statins). Across these additional cases, hypercholesterolemic patients receiving statins displayed more pronounced tumor regression trajectories than hypercholesterolemic patients without statins, further supporting the beneficial effect of cholesterol reduction on treatment efficacy (Figure S25A). Immunohistochemical analysis of surgically resected brain metastatic specimens similarly demonstrated that hypercholesterolemic patients exhibited elevated EGFR/AKT pathway activation, robust PDK1 and LDHA expression, increased M2‐type macrophage infiltration, and reduced CD8^+^ T‐cell density compared with normocholesterolemic patients. Importantly, these pro‐tumorigenic and immunosuppressive alterations were markedly attenuated in hypercholesterolemic patients receiving statin therapy (Figure 8H; Figure S26A). To ensure objective assessment, we additionally performed quantitative analyses of p‐EGFR, CD206, CD8a, and other relevant markers across five cases per group, which consistently confirmed significant differences: H‐Cho patients displayed high p‐EGFR/p‐AKT and CD206 with reduced CD8a, whereas statin co‐treatment reversed these trends in a manner consistent with our preclinical findings (Figure S26B). For full transparency and traceability, each imaging case and IHC sample is annotated with the corresponding patient medical record number (ID), enabling clear linkage to diagnostic procedures and treatment histories and ensuring robustness of the clinical evidence.

Taken together, these clinical observations provide compelling evidence that elevated serum cholesterol level contributes to LUAD‐BM progression by activating EGFR/AKT signaling, enhancing glycolysis, and fostering an immunosuppressive microenvironment. Importantly, statins synergize with EGFR‐TKI, especially in patients with hyperlipidemia, offering a promising combinational strategy for managing LUAD‐BM.

## 3 Discussion

LUAD is the most prevalent subtype of non‐small cell lung cancer (NSCLC), characterized by a high incidence of brain metastases (BM), profound chemoresistance, and dismal prognosis [45]. LUAD‐BM progression undergoes a multistep cascade involving primary tumor growth, intravasation of tumor cells, circulation and survival of CTCs, extravasation into the brain parenchyma, and eventual colonization and proliferation within the brain [9], offering multipletherapeutic intervention targets. Cholesterol is essential for membrane integrity and fluidity [46], and has been implicated in tumor progression and therapy response across multiple malignancies [16, 17, 18, 30]. Building on established links between lipid metabolism and brain metastasis, our study positions cholesterol as a downstream functional mediator within lipid‐metabolic programs that couple tumor‐intrinsic oncogenic signaling with tumor‐extrinsic barrier and immune niche remodeling during LUAD‐BM. This integrated view is supported by complementary in vivo dissemination/seeding models and mechanistic in vitro platforms, collectively delineating how systemic hypercholesterolemia can amplify brain colonization and therapeutic resistance.

### 3.1 Distinction from Prior Lipid‐Metabolism Literature

Multiple studies have shown that brain metastatic colonization requires broad lipid‐metabolic adaptation. Jin et al. identified SREBP1 as a master regulator that rewires fatty acid synthesis and uptake to enable brain metastatic colonization [25]. Ferraro et al. further emphasized that lipid transport, synthesis, and β‐oxidation support survival and outgrowth of metastatic cells within the nutrient‐restricted brain niche [26]. Parida et al. additionally showed that limiting mitochondrial plasticity and associated lipid catabolism can suppress brain metastasis [27]. Our findings refine this framework by identifying cholesterol—a major end product of lipid metabolism and membrane‐organizing lipid—as a proximal effector that directly modulates specific signaling and microenvironmental nodes in LUAD‐BM.

Mechanistically, we demonstrate that cholesterol directly engages EGFR and stabilizes membrane EGFR by attenuating ubiquitin–proteasome–mediated degradation, thereby sustaining AKT/NF‐κB signaling and driving glycolytic reprogramming and EMT. This receptor‐stability mechanism differs from upstream transcriptional lipid regulators such as SREBP1 by defining an actionable, downstream step linking lipid state to persistent EGFR signaling and EGFR‐TKI resistance. Consistent with prior evidence that cholesterol‐rich membrane microdomains can modulate receptor tyrosine kinase activity and therapy response [23, 24], our data further provide a mechanistic explanation for how hypercholesterolemia may reduce sensitivity to EGFR‐TKIs in LUAD.

Beyond tumor‐intrinsic signaling, our results highlight tumor‐extrinsic mechanisms unique to brain metastasis. The BBB represents a critical rate‐limiting barrier to brain colonization [12, 47, 48, 49, 50, 51]. We show that cholesterol compromises BBB integrity by decreasing endothelial membrane fluidity and accelerating ubiquitin–proteasome–dependent Claudin‐5 degradation, thereby facilitating tumor cell extravasation. In parallel, the brain immune microenvironment exerts strong selective pressure during metastatic seeding and outgrowth [13, 52]. We demonstrate that cholesterol promotes microglial M2 polarization through lipid raft–associated IL‐4R/JAK1/STAT6 activation, linking membrane microdomain organization to immunosuppressive niche remodeling. To avoid overinterpretation, we explicitly note that local brain cholesterol concentrations were not directly quantified; lipid raft markers were used as functional proxies for cholesterol‐enriched membrane microdomains. Together, these coordinated tumor‐intrinsic and tumor‐extrinsic effects provide an integrated model for how systemic hypercholesterolemia can preferentially enhance LUAD brain colonization compared with extracranial sites.

It is worth noting that we systematically documented the tumor formation rates, lesion counts, and anatomical distribution across all experimental models. Our findings indicate that the tail vein injection model is particularly well‐suited for investigating the proliferation and metastatic progression of primary lung cancer. This model predominantly supports lung colonization (79.2%) but exhibits a relatively low brain metastasis incidence (20.8%), with occasional metastases to the bone (20.8%) and liver (12.5%). In contrast, for studies specifically focused on brain metastasis, we recommend the intracardiac injection model, which achieves a substantially higher brain metastasis rate (58.3%) while still recapitulating metastatic dissemination to the lung (47.2%) and bone (22.2%).

### 3.2 Clinical Relevance and Translational Implications

The translational implications of our findings are particularly relevant for EGFR‐mutant LUAD‐BM, where intracranial progression and acquired resistance remain frequent despite third‐generation EGFR‐TKIs such as osimertinib [48, 49, 50, 51, 52, 53, 54]. Acquired resistance typically emerges within months in many patients, and brain metastases remain challenging due to the BBB and the specialized neural immune microenvironment [12, 13, 53, 54]. Prior clinical observations have associated hypercholesterolemia with reduced EGFR‐TKI responses in EGFR‐mutant NSCLC [24], but mechanistic explanations and brain‐specific validation have been limited.

In our retrospective LUAD‐BM cohort, baseline fasting hypercholesterolemia at lung cancer diagnosis was independently associated with shorter metastasis‐free intervals and worse survival outcomes, while statin exposure was associated with improved outcomes. Importantly, cholesterol status was defined using fasting lipid profiles obtained at the time of lung cancer diagnosis (prior to systemic therapy), reducing bias from treatment‐induced lipid fluctuations and strengthening temporal interpretability. To mitigate immortal time bias and time‐related confounding in drug‐exposure analyses, statin exposure was modeled as a time‐dependent covariate in Cox regression, thereby strengthening the robustness of the associations observed (Tables S5–S7). Although residual confounding is inherent to retrospective studies, these design and analytic choices increase confidence that the clinical associations are not solely artifacts of follow‐up structure.

From a translational perspective, our mechanistic data suggest a testable model: hypercholesterolemia may define a metabolic context in which EGFR signaling persistence, BBB permissiveness, and microglia‐driven immunosuppression jointly reduce intracranial control under EGFR‐TKIs, whereas cholesterol lowering may partially restore therapeutic sensitivity. This interpretation is consistent with our in vivo results showing that atorvastatin reverses cholesterol‐driven EGFR stabilization and metabolic rewiring, mitigates BBB disruption, alleviates microglia‐dependent immunosuppression, and synergizes with EGFR‐TKIs to suppress brain metastatic progression. Notably, these findings support statins as a sensitizing adjunct rather than a standalone anticancer therapy, aligning with their established clinical use and safety, and with prior reports of pleiotropic anti‐inflammatory and vascular effects [55, 56].

A key implication is patient stratification. If validated prospectively, baseline TC/LDL‐C thresholds could be used to enrich clinical trials for patients most likely to benefit from statin co‐therapy (e.g., hypercholesterolemic EGFR‐mutant LUAD‐BM). Future trials should predefine lipid thresholds, specify statin type/intensity and adherence monitoring, and control for major confounders such as cardiovascular comorbidities, steroid exposure, prior radiotherapy, and line of systemic therapy—variables known to influence intracranial outcomes and treatment tolerance [57, 58, 59, 60, 61]. In addition to overall survival, intracranial progression‐free survival, intracranial response rate, and MRI‐based response kinetics would provide clinically actionable endpoints aligned with the proposed mechanisms.

Obesity‐related studies have reported complex and sometimes paradoxical associations between body mass index (BMI) and brain metastasis risk. Tyagi et al. described an “obesity paradox” in which low BMI correlated with increased brain metastasis incidence in specific contexts [62]. In our LUAD‐BM cohort, BMI was not an independent predictor of key clinical endpoints after adjustment for molecular subtype and treatment exposure, suggesting that cholesterol metabolism may represent a more relevant and actionable metabolic determinant than generalized adiposity in EGFR‐driven LUAD‐BM. This distinction is clinically meaningful because lipid levels are directly measurable and modifiable, whereas BMI is an imprecise surrogate that may not capture the metabolically relevant state during targeted therapy.

## 4 Conclusion

**FIGURE 9:**
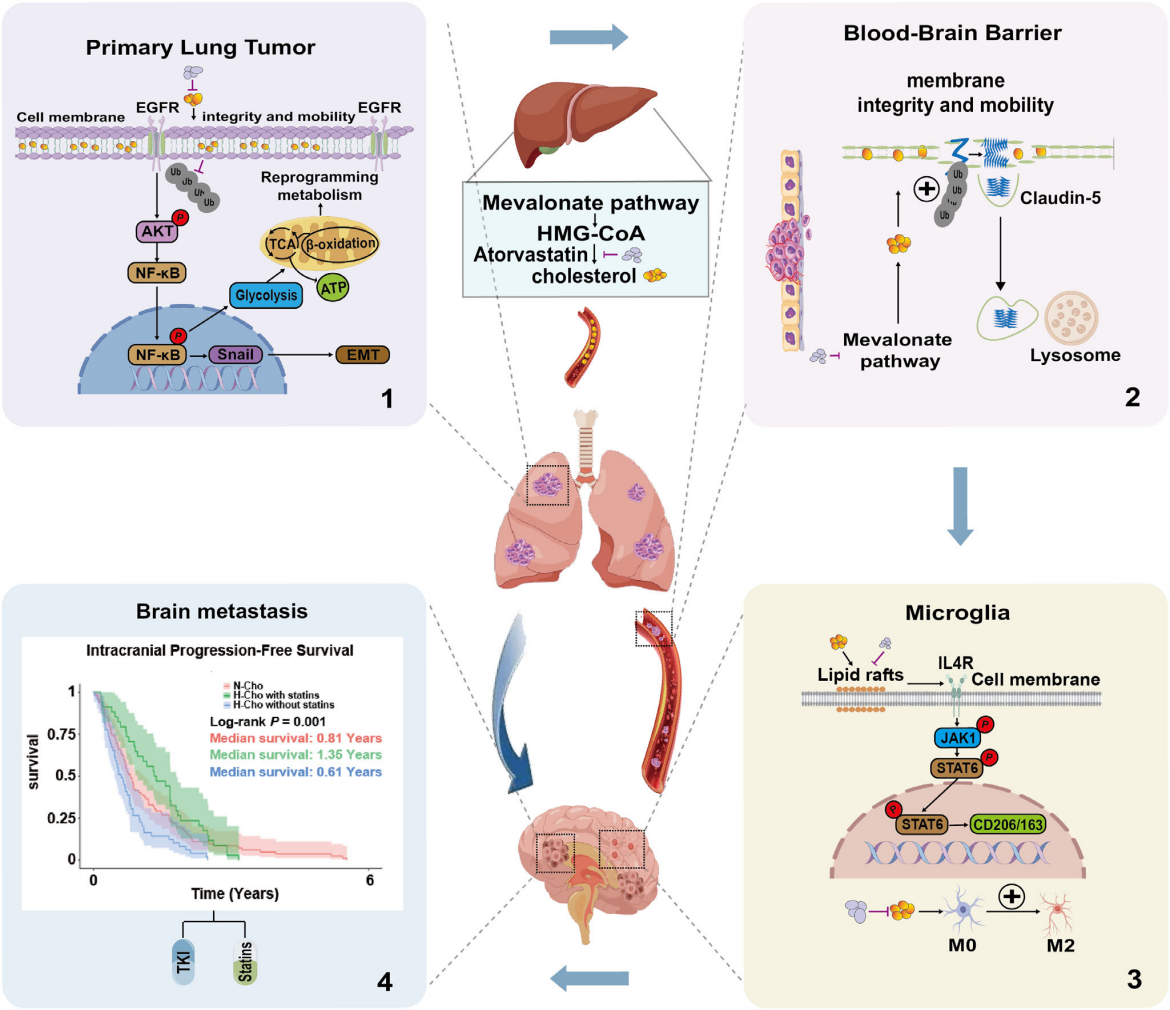
Schematic illustration of cholesterol‐driven LUAD‐BM and the therapeutic role of statins. Cholesterol promotes LUAD‐BM through multiple mechanisms: (1) In primary lung tumors, cholesterol stabilizes EGFR by reducing ubiquitination and proteasomal degradation, thereby activating AKT/NF‐κB signaling, enhancing glycolysis, and EMT. (2) In the BBB, cholesterol disrupts membrane integrity by promoting Claudin‐5 degradation through the mevalonate pathway and ubiquitin–lysosomal trafficking. (3) In the brain microenvironment, cholesterol facilitates IL‐4Rα recruitment into lipid rafts on microglia, leading to JAK1/STAT6 signaling activation and polarization into M2‐like phenotypes, which support tumor colonization. (4) These changes collectively enhance LUAD‐BM progression and reduce the efficacy of EGFR‐TKIs. Atorvastatin, an HMG‐CoA reductase inhibitor, counteracts these effects by suppressing cholesterol biosynthesis and reprogramming the tumor and brain microenvironment, thereby improving survival and treatment response.

In summary, our study extends established lipid metabolism–brain metastasis frameworks by defining cholesterol as a downstream mediator that links EGFR signaling persistence, BBB disruption, and microglia‐dependent immunosuppression to LUAD brain metastatic progression and therapeutic resistance. By demonstrating that atorvastatin counteracts these interconnected mechanisms and enhances EGFR‐TKI response in vivo—together with supportive retrospective clinical associations—our findings highlight a clinically feasible metabolic‐targeting strategy that merits prospective evaluation in LUAD‐BM (Figure 9).

### 4.1 Limitations and Future Directions

Despite the comprehensive mechanistic insights gained from this study, several directions for future refinement may further broaden our understanding. First, although our xenograft models provided a robust platform for validating the protumorigenic role of cholesterol in brain metastasis, genetically engineered mouse models (GEMMs) such as Kras^LSL‐G12D/+ or EGFR^L858R/T790M knock‐ins may offer additional physiological nuance by capturing endogenous tumor initiation and the evolving interactions between tumor, stroma, and systemic metabolism. These complementary perspectives would help extend, rather than alter, the mechanistic framework established here. Second, our characterization of cholesterol‐induced microglial M2 polarization—supported by BV‐2 and HMC3 cell models and in vivo microglia depletion—lays a solid foundation for dissecting neuroimmune mechanisms. Future incorporation of microglia‐specific reporter systems (e.g., CX3CR1‐GFP or TMEM119‐CreER) or lineage‐tracing strategies could provide finer spatial‐temporal resolution of microglial dynamics and the activation of the IL‐4R/JAK1/STAT6 pathway within the metastatic niche. Finally, the identification of lipid‐related biomarkers and the development of non‐invasive liquid biopsy may substantially advance the translational potential of our findings. In this regard, our group is currently conducting a prospective clinical study aimed at establishing a non‐invasive liquid biopsy to monitor brain‐metastatic risk and therapeutic response (Clinical registration: ChiCTR2500111023).

## 5 Experimental Section

### 5.1 Ethical Approval and Informed Consent

A total of 200 patients histologically diagnosed with lung adenocarcinoma brain metastasis (LUAD‐BM) at Huanhu Hospital Affiliated to Tianjin Medical University between 2022 and 2024 were enrolled in this study. Ethical approval was granted by the Ethical Committee of Huanhu Hospital Affiliated to Tianjin Medical University (Approval No.: 2025‐180). In strict adherence to the Declaration of Helsinki and Good Clinical Practice (GCP) guidelines, all patients provided written informed consent for sample collection and data analysis prior to surgery. Additionally, all animal experimental protocols were approved by the Institutional Animal Care and Use Committee (IACUC) of Tianjin Medical University General Hospital (Approval No.: IRB2024‐DWFL‐033).

### 5.2 Clinical Registration Number

Prospective clinical trial registration number: ChiCTR2500111023 (focused on establishing a non‐invasive liquid biopsy for monitoring brain metastatic risk and therapeutic response).

### 5.3 Proteomic Datasets

Proteomic datasets generated in this study have been deposited in the OMIX, China National Center for Bioinformation/Beijing Institute of Genomics, Chinese Academy of Sciences (https://ngdc.cncb.ac.cn/omix: accession no. OMIX013794).

### 5.4 Cell Lines and Reagents

The cell lines used in this study included human LUAD cell lines A549 (ATCC, CCL‐185, EGFR wild‐type, RRID: CVCL_0023) and PC‐9 (ATCC, EGFR 19del, △E746‐A750, RRID: CVCL_B260), as well as murine LUAD cell lines LLC (ATCC, low EGFR expression, RRID: CVCL_4358) and its brain‐tropic derivative LLC‐BMT3. PC‐9 cells harbor an EGFR exon 19 deletion (ΔE746–A750), representing the most common EGFR mutation in human LUAD, and were used to model EGFR‐mutant disease and to evaluate cholesterol–EGFR–TKI interactions in vivo. A549 cells, which are EGFR wild‐type, were used as controls to assess the EGFR dependency of cholesterol‐mediated effects. These LUAD cell lines were maintained in DMEM high‐glucose medium (Gibco, USA) supplemented with 10% FBS (Gibco, USA) and 1% penicillin‐streptomycin (Gibco, USA). LLC‐BMT3 was derived from brain metastatic tissues of C57BL/6 mice through three rounds of iterative isolation and puromycin (2 µg/mL, MedChemExpress, China) resistance screening. Murine brain microvascular endothelial cells (Bend.3, Wuhan Punuo Sai Life Technology, China) and microglia (BV‐2, ATCC, RRID: CVCL_0182) were maintained in DMEM high‐glucose medium (Gibco, USA) supplemented with 10% FBS (Gibco, USA) and 1% penicillin‐streptomycin (Gibco, USA), while human microglia (HMC3, ATCC, RRID: CVCL_II76) were cultured in MEM (with NEAA, Gibco, USA) containing 10% FBS and 1% penicillin‐streptomycin. All cells were incubated at 37°C in a 5% CO_2_ incubator, with drug treatments initiated 24 h post‐seeding and tested for Mycoplasma using MycoAlert Mycoplasma Detection Kit (Lonza, China) regularly. Cholesterol, atorvastatin, cycloheximide (CHX), chloroquine (CQ), MG132, and pharmacological inhibitors (Osimertinib, MK‐2206, JSH‐23, AS1517499, and Filgotinib) were purchased from Selleck (China) and dissolved in serum or DMSO before use. For animal studies, drugs were initially dissolved in DMSO and subsequently diluted in 0.5% carboxymethylcellulose sodium (CMC‐Na; Selleck, China) to the required concentrations.

### 5.5 Establishment of High Brain‐Metastatic LLC‐BMT3 Cell Line

C57BL/6J mice were obtained from the Beijing Vital River Laboratory Animal Technology Co., Ltd. (Beijing, China). All mice were housed in a specific pathogen‐free (SPF) breeding barrier with individual ventilated cages. Tribromethyl alcohol (MedChemExpress, China) was used to anesthetize mice. Mice should be sacrificed by cervical dislocation after anesthesia in the following conditions: near death or immobile, have significant weight loss, or are unable to feed or drink. LLC‐Luc cells were injected into the carotid artery of C57BL/6J mice. Brain metastases were confirmed by in vivo bioluminescence imaging after 3–4 weeks. Brains were harvested aseptically, and GFP‐positive regions were dissected, enzymatically digested, filtered, and subjected to puromycin selection to derive brain‐tropic cells (LLC‐BMT1). This selection process was repeated three times to generate LLC‐BMT3 cells.

### 5.6 High‐Cholesterol Mouse Model

Male C57BL/6J mice or (4 weeks old) were fed a high‐fat diet (HFD, D12492, Research Diets, 60 kcal% from fat, USA) for 12 weeks to induce hypercholesterolemia. For cholesterol‐lowering treatment, atorvastatin (10 mg/kg/day) was administered orally. Body weight was monitored bi‐daily, and serum total cholesterol (TC), LDL‐C, HDL‐C, and triglycerides (TG) levels were assessed in week 12. Hepatic lipid accumulation was evaluated by Oil Red O staining.

In selected experiments, male C57BL/6J mice or BALB/C nude mice (4 weeks old) were fed a Western diet (WD, D09062501, Research Diets, western diet with 1.25% cholesterol, 42 kcal% from fat, USA) for 6 weeks to induce hypercholesterolemia. For cholesterol‐lowering treatment, atorvastatin (10 mg/kg/day) was administered orally. Serum TC, LDL‐C, HDL‐C, and TG levels were assessed in week 6.

### 5.7 Brain Metastasis Model

Tail Vein Injection Metastasis Model: Luciferase‐labeled LLC‐BMT3 cells were harvested during the logarithmic growth phase, washed twice with sterile PBS, and resuspended at a concentration of 1 × 10^7^ cells/mL. C57BL/6J mice were gently restrained, and 100 µL of the cell suspension (equivalent to 1 × 10^6^ cells) was slowly injected into the lateral tail vein using a 29‐gauge insulin syringe. Following injection, mice were monitored until full recovery and subsequently maintained under standard conditions for longitudinal assessment of metastatic progression.

Intracardiac Injection Brain Metastasis Model: For the establishment of the intracardiac brain metastasis model, luciferase‐labeled LLC‐BMT3 or PC‐9 cells were harvested, washed, and resuspended in sterile PBS. A total of 5 × 10^4^ LLC‐BMT3 cells or 1.5 × 10^5^ PC‐9 cells in 50 µL PBS was prepared for each mouse. Mice were anesthetized with 1.5% sodium pentobarbital (50 mg/kg, intraperitoneally) and placed in a supine position. Using a 26‐gauge needle, the cell suspension was slowly injected into the left cardiac ventricle, with correct placement confirmed by pulsatile backflow of arterial blood. After injection, mice were monitored until full recovery from anesthesia and then maintained under standard conditions for subsequent bioluminescence imaging and survival analysis.

Tumor burden was monitored using a bioluminescence imaging apparatus (BLI, IVIS, USA), and Kaplan‐Meier curves were used to visualize survival time. After modeling, the tumor tissues were carefully harvested, fixed by formalin, embedded in paraffin, and used for immunohistochemistry (IHC) analysis.

### 5.8 H&E and Immunohistochemical Staining

Tumor sections were prepared from formalin‐fixed paraffin‐embedded (FFPE) tissue blocks, then dewaxed, hydrated, and treated for antigen retrieval in citrate buffer (pH = 6) at 100 °C for 20 min. For H&E staining, brain sections were stained by hematoxylin and eosin. For IHC staining, after dewaxing, hydration, and antigen repair, tissue sections were blocked with goat serum for 1 h at room temperature and then incubated with the appropriate primary antibodies at 4 °C overnight. The antibodies were listed in Table S2. After washing with PBS, the sections were incubated with horseradish peroxidase (HRP)‐conjugated secondary antibodies for 1 h at room temperature. After DAB was developed, the sections were covered with coverslips and observed with a bright‐field microscope.

### 5.9 In Vitro Blood‐Brain Barrier (BBB) Model

To assess cholesterol‐mediated BBB disruption and tumor cell transmigration in vitro, we utilized Bend.3 murine brain microvascular endothelial cells (a well‐established model for BBB tight junction formation) to construct an endothelial monolayer. Confluent Bend.3 cells were treated for 48 h with either DMSO (control), 12.5 µM water‐soluble cholesterol (Cho), 12.5 µm Cho + 10 µm atorvastatin (ATO), or 12.5 µm Cho + Claudin‐5 overexpression (via pcDNA3.1‐Claudin‐5 transfection, validated by immunoblotting). Fluorescently labeled LLC‐BMT3 cells (DiI‐stained) were then seeded onto the apical surface of the treated Bend.3 monolayers; after 24 h of co‐incubation, non‐migrated cells were removed, and transmigrated LLC‐BMT3 cells (on the basolateral side) were visualized by fluorescence microscopy (scale bar: 50 µm) and quantified across 3 random fields per sample to evaluate BBB permeability.

### 5.10 Clinical Sample Collection and Analysis

A retrospective cohort of LUAD‐BM patients diagnosed and treated at Huanhu Hospital Affiliated to Tianjin Medical University between January 2022 and December 2024 was analyzed. Baseline fasting serum lipid levels, including total cholesterol (TC) and low‐density lipoprotein cholesterol (LDL‐C), were measured under fasting conditions at the time of lung adenocarcinoma diagnosis, prior to initiation of systemic anticancer therapy. Hypercholesterolemia was defined according to standard clinical diagnostic criteria as TC > 5.18 mmol/L and/or LDL‐C > 3.37 mmol/L, whereas values at or below these thresholds were classified as normal cholesterol. Out of 809 screened patients, 200 met all eligibility criteria and were included in the final analysis. Patients were stratified according to baseline cholesterol status, with further subdivision of hypercholesterolemic patients based on statin use. Statin therapy was initiated prior to cancer diagnosis in all statin users, with good documented adherence.

Clinical variables collected included demographic characteristics, lifestyle factors, comorbidities, EGFR mutation status, treatment history, and survival outcomes. Statin exposure was incorporated as a time‐dependent covariate in Cox regression analyses to minimize time‐related confounding. Immunohistochemistry was performed on formalin‐fixed paraffin‐embedded brain metastatic tissues using antibodies against p‐EGFR, p‐AKT, LDHA, CD206, and CD8.

### 5.11 Statistical Analysis

All statistical analyses were performed using GraphPad Prism 8 software and R language (v3.6.2). An independent sample Student's t‐test was used for comparison between two experimental groups. One‐way and two‐way analyses of variance (ANOVA) were used to compare at least three experimental groups. The error bars in the figures represent the mean ± standard deviation (SD). Restricted cubic splines (RCS) are known to be able to accurately capture the dose‐response relationship between exposure and outcome. Hence, we constructed RCS curves for the derived logistic regression model to provide additional validation for the non‐linear relationships detected between TC, LDL‐C levels, and the level of metastasis. Cox proportional hazards models for clinical data. A *P* value of less than 0.05 was considered statistically significant, defined as ns, not significant, ^*^ *p* < 0.05, ^***^ *p* < 0.001, ^****^ *p* < 0.0001.

## Author Contributions

Y.C., X.C., and X.S. contributed equally to this work. C.K. conceived the project and designed the research. X.L. provided insights on research design. Y.C., X.Y., M.W., and X.C. performed bioinformatics analyses. Y.C., X.S., H.L., Y.C., and A.L. provided clinical samples and performed histopathological assessments. X.C., Q.Z., Q.W., J.Z., and Y.W. provided technical support in experiments. Y.C., X.S., Q.C., and X.C. conducted experiments. Y.C., C.K., and X.L. analyzed data and wrote the manuscript. All authors discussed the results and approved the manuscript.

## Supporting information




**Supporting File 1**: advs73843‐sup‐0001‐SuppMat.docx.

**Supporting File 2**: advs73843‐sup‐0002‐TableS1‐S4.xlsx.

**Supporting File 3**: advs73843‐sup‐0003‐TableS5‐S7.docx.

## Data Availability

The data that support the findings of this study are available from the corresponding author upon reasonable request.
