# Supplementary material for: Cholesterol Promotes Lung Adenocarcinoma Brain Metastasis by Stabilizing EGFR Protein to Drive EMT, Metabolic Reprogramming, and Premetastatic Niche Formation
Source: Adv Sci (Weinh). 2026 Jan 21;13(17):e73843. doi: 10.1002/advs.73843 (PMC13042990; doi:10.1002/advs.73843)
Supplement: Supplementary file 1 — Supporting File 1: advs73843‐sup‐0001‐SuppMat.docx. [file ADVS-13-e73843-s003.docx]

**Supporting Information**

Cholesterol Promotes Lung Adenocarcinoma Brain Metastasis by Stabilizing EGFR Protein to Drive EMT, Metabolic Reprogramming, and Premetastatic Niche Formation

Ying Chen, Xiaoteng Cui, Xinyi Shi, Xu Yang, Yujing Cao, Haolin LI, Qi Zhan, Qixue Wang, Ang Li, Qihong Cheng, Yunfei Wang, Junhu Zhou, MingJie Wang, Chunsheng Kang*, and Xiaomin Liu*

**Supplementary materials and methods**

*Immunofluorescence*: Cells were seeded onto sterilized 10 mm glass coverslips in 12-well plates. After drug treatment, cells were fixed with 4% paraformaldehyde (PFA) (Solarbio, China) at room temperature for 15 minutes, permeabilized with or without 0.1% Triton X-100, and blocked with 5% BSA. Primary antibodies (Table S2, Supporting Information) were applied overnight at 4 ℃, followed by incubation with fluorescence-conjugated secondary antibodies. Nuclei were counterstained with DAPI (Sigma, Germany). Mice underwent transcranial perfusion with 4 % PFA, followed by extraction and fixation of the brains in 4 % PFA for 24 h. After antigen retrieval with citric acid, brain sections were incubated with primary antibodies at 4 ℃ overnight. The next day, the sections were washed three times in phosphate-buffered saline (PBS) and incubated for 1 h at room temperature with the corresponding secondary antibody. DNA was stained with DAPI for 10 min at room temperature. If more than two target proteins needed to be stained, the sections were incubated in Tyramide Signal Amplification (TSA) solution at 37 ℃ for 30 min after the secondary antibody incubation. The steps for antigen retrieval to TSA were then repeated until all proteins were stained, followed by DAPI staining. Coverslips were mounted using antifade reagent and imaged using confocal microscopy. All the images were observed by FV-1000 laser scanning confocal microscope (Olympus Corporation, Japan). The antibodies were listed in Table S2 (Supporting Information).

*Western Blot Analysis and Co-Immunoprecipitation*: Protein was extracted from cells with radioimmunoprecipitation assay (RIPA) protein lysis buffer (Solarbio, China) containing freshly of protease inhibitor cocktail and PMSF. Then, 30 µg of protein was subjected to SDS-PAGE. Proteins were separated by gel electrophoresis and transferred to polyvinylidene fluoride (PVDF) membranes. The membranes were blocked by 5% bovine serum albumin (BSA) for 2 h at room temperature, followed by incubation with primary antibodies at 4 ℃ overnight. After washing three times with phosphate buffered saline-Tween 20 (PBST), the membrane incubated in blocking buffer with secondary antibodies. Chemiluminescence detection was performed using G:BOXF3 (Syngene, UK) with Western HRP Substrate (Sigma, Germany). For co-IP analysis, the cells were lysed by IP lysis buffer (Beyotime Biotechnology, China) and then incubated with Protein A/G PLUS-Agarose (Selleck, China) and primary antibodies on a shaker at 4 ℃ overnight. After washing the sample five times with IP lysis buffer, western blot analysis was performed. The antibodies were listed in Table S2 (Supporting Information).

*Proteomic LC-MS/MS analysis*: Proteomic LC-MS/MS analysis was performed by PROTEINT (Tianjin, China). Every cell sample was thoroughly mixed with 200 µL lysis buffer (4% SDS, 1% Protease inhibitor cocktail (Sigma) and put on ice for 15 min. Then the samples were ultrasonicated for 10 min (3s-on, 3s-off). The lysis samples were heated at 95 ℃, 750 rpm for 1 h. The concentration was measured by BCA method. The samples were added with 2 µL 500 mM DTT and kept at 56 ℃ for 1 h. Then 20 µL 500 mM IAM was added and the samples were kept at room temperature blocked of light for 45 min. 60 µg protein was purified with SP3 beads and 20 µL digestion buffer (50 mM NH_4_CO_3_, 50 mM CaCl_2_, 2.4 µg trypsin) was used to resuspend the beads. The proteins were digested overnight at 37 ℃ and 1 µg trypsin was further added to digest for another 3 h at 37 ℃. The peptides were then purified by SP3 beads and eluted with 20 µL 2% ACN. Equal amounts of iRT (purchased from Biognosys) were added into each DIA run on a Thermo Scientific U3000 nanoflow LC system followed by Q Exactive HF mass spectrometer. Peptide samples were dissolved with loading buffer (2% ACN) and separated on a 150 μm ID × 30 cm column (C18, 1.9 μm, 120 Å, Dr. Maisch GmbH) using a 150 min gradient (A: 2% ACN, 0.1% FA; B: 80% ACN, 0.1% FA; 0-3 min, 3 to 9% B; 3-127 min, 9% to 63% B; 127-131 min, 63% B; 131-149 min, 63-3% B) with a flow rate of 600 nL/min. The spray voltage was set at 2,000 V in positive ion mode, and the ion transfer tube temperature was set at 270. For DIA a 60 K resolution MS scan @ m/z 200 MS was performed, and the AGC target value was set at 1e6 or 20 ms of max injection time by the orbitrap mass analyzer (350 – 1,500 m/z). The MS/MS AGC target value was set at 1e6 with an auto setting of max injection time generated by HCD fragmentation at a resolution of 30,000 @ m/z 200. The NCE was set at NCE 28%. For proteome DIA MS runs, fragment analysis was subdivided into 40 DIA isolation windows with different widths depending on the DIA search results. MS scans were also performed before each DIA cycle. The DIA data were searched against the human UniProt database (20,365 sequences) using DIA-NN (v1.8.1) with default settings, with trypsin/P digest rule, high protein and peptide confidential level and FDR of 0.01. The output quantified protein proteins with at least 30% appearance in all samples were-selected for further analysis. Proteins were normalized with Median normalization method. The absent value was filed with 1000.

*RNA Extraction and Quantitative RT-PCR*: Cells were lysed in TRIzol Reagent (Invitrogen, USA). The lysate was mixed with chloroform and centrifuged at 12,000 rpm for 15 min at 4 ℃. Transfer the upper aqueous phase to a clean tube and add an equal volume of isopropanol. The samples were thoroughly mixed and stored at −20 ℃ overnight. The total RNA concentration was measured and used for qRT-PCR. According to the manufacturer's protocol, cDNA was synthesized from 2 µg of total RNA using a reverse transcription kit purchased from Promega (Madison, USA). qRT-PCR using the DNA Engine Opticon 2 Two-Color qRTPCR detection system (Bio-Rad Laboratories, USA) and the results were normalized to the Actin. ΔΔCt method was used for quantification analysis. The primers were in Table S3 (Supporting Information).

*Seahorse assay XFe24 extracellular flux analysis*: The Seahorse XF Glycolysis Stress Test Kit (Agilent, USA) was used to evaluate extracellular acidification rate (ECAR). A549, PC-9 and LLC cells were seeded in Seahorse XFe24 plates (Agilent, USA). To measure the effects of cholesterol and inhibitors was added to the culture medium. Seahorse XF Glycolysis Stress Test Kit (Agilent, USA) were used following the manufacturer’s protocol. Briefly, a sensor cartridge (Agilent, USA) was hydrated using the Seahorse XF Calibrant at 37 ℃ in a non-CO_2_ incubator overnight. The medium was prepared by supplementing Seahorse XF DMEM medium (Agilent, USA) with essential additives with glucose (100 mM), oligomycin (100 μM), and 2-deoxyglucose (2-DG, 500 mM) and warmed to 37 ℃. The compounds included in the kit were solubilized by the medium and loaded into the ports on the sensor cartridge. After calibrating the prepared sensor cartridge and placing the cell culture microplate at 37 ℃ in a non-CO_2_ incubator for 45 min to 1 h, the extracellular acidification rate (ECAR) was measured on a Seahorse XFe24 Analyzer (Agilent, USA).

*FRAP Membrane Fluidity Assay*: We incubated the cells with a 1 μg/mL DiI solution (Sigma, Germany) for 10 min in a confocal culture dish at room temperature, and then washed the cells 3 times with PBS. For the laser scanning confocal microscope, we used a ×100 oil immersion objective in the FV-1000 laser scanning confocal microscope. We photo-bleached a limited area (~1×1 cm^2^) of the marking film with a 100 % laser pulse. Scans with a total duration of 30 s resulted in complete or near-complete photobleaching. After photobleaching, the fluorescence intensity was recorded every 10 seconds for 5 minutes. After background normalization, changes in fluorescence intensity were plotted over time to generate a fluorescence recovery curve.

*Evans Blue permeability assay*: Blood–brain barrier integrity was evaluated using Evans Blue (EB) extravasation. Mice were injected with 2% Evans Blue solution (2 mL/kg, i.v. via the tail vein). After 2 h, animals were deeply anesthetized and transcardially perfused with cold PBS until the effluent from the right atrium became colorless, to remove intravascular dye. Brains were then carefully removed and photographed to document gross EB extravasation. For quantitative analysis, brain tissues were weighed, minced, and incubated in formamide (1 mL/100 mg tissue) at 60 °C overnight to extract EB. Samples were centrifuged (e.g., 12 000 × g, 20 min), and the absorbance of the supernatant was measured at 620 nm using a microplate reader. Evans Blue content was calculated according to a standard curve and expressed as μg EB per g brain tissue or as relative optical density.

*Transmission electron microscopy (TEM)*: Brains were harvested, and the ischemic cerebral cortex was cut into 2 mm^3^ pieces and fixed in 2.5 % glutaraldehyde (Servicebio, China) for 2 h at room temperature, then treated with 1 % osmium tetroxide solution, followed by dehydration in graded ethanol and drying with hexamethyldisilazane. After embedding into epoxy resin, samples were sliced into sections with a thickness of 70 nm, and stained with 2 % uranyl acetate and lead citrate. Finally, slides were sputtered with gold-palladium and imaged by an H7760 microscope.

*EdU Cell Proliferation Assay*: Cell proliferation was assessed using a Click-iT EdU Imaging Kit (Beyotime Biotechnology, China). After 2 h of EdU incubation, cells were fixed, permeabilized, and stained per manufacturer instructions. The percentage of EdU-positive cells was determined by fluorescence microscopy.

*In Vitro Co-culture System*: To simulate microglia-tumor cell interactions, BV-2 microglia (pre-treated with DMSO, cholesterol, or cholesterol + ATO for 48 h) were seeded in the lower chamber and tumor cells in the upper chamber. After 48 h, LUAD cells were harvested and subjected to invasion, and proliferation assays.

*Cell invasion assays*: We used 24-well Transwell plates with 8 μm pore filters (Corning, Corning, USA) for invasion assays. The inserts were pre-coated with 50 μl of diluted Matrigel (DMEM, 1:9). The filters were air-dried and hydrated with 60 μl serum-free DMEM. Approximately 5×10^4^ cells were suspended in 200 μl of DMEM medium with 1 % FBS and placed in the upper chamber, while 600 μl DMEM medium with 20 % FBS was added to the lower chamber. After 48 h of incubation, cells were fixed in 4 % paraformaldehyde for 30 min and stained with 0.1 % crystal violet for 20 min. Next, non-invading cells on the upper surface were removed. Cells on the underside of filters were counted and photographed in five microscopic fields. All experiments were performed at least three times. 3D tumor sphere invasion assays, A549, PC-9 and LLC cells (1-3×10^3^/well) with targeted gene knockdown or overexpression were seeded onto low adherence 96-well plates. After sphere formation, invasion gel (50 μ/well; R&D Systems, USA) was added to the wells, and plates were incubated for 48 h. Images were acquired at specifc intervals to examine the invasion ability of modified tumor cells.

*Wound-healing migration assay*: Cells were seeded in 6-well plates and grown to a confluent monolayer for about 12-24 h. The monolayer was scratched with a 20 μl pipette tip. The cells were then washed with PBS and cultured in serum-free DMEM medium for 48 h. Migration was photographed and recorded at 0 and 48 h under a microscope.

*Cell proliferation assays*: Cells were counted and seeded in 96-well plates (800-1000 cells/well). After incubation for 24 h, 10 μl of Cell Counting Kit-8 (CCK-8, Dojindo, Japan) was added to the culture medium and incubated for 2 h at 37 ℃. Then, the optical density (OD) value at 450 nm was measured by using SpectraMax Paradigm (Molecular Device, USA). All of the values were standardized by comparison with the data from the untreated cells. Three independent experiments were performed.

*Co-culture of CD8⁺ T cells with microglia and flow cytometry analysis*: Spleens from C57BL/6J mice were mechanically dissociated and passed through a 70-µm cell strainer. After red blood cell lysis, CD8⁺ T cells were purified using a negative-selection magnetic bead kit according to the manufacturer’s protocol, and resuspended in RPMI-1640 supplemented with 10% FBS. BV-2 microglial cells were pre-treated with DMSO, cholesterol (Cho, 12.5 µmol/L), or cholesterol plus atorvastatin (ATO, 5 µmol/L) for 48 h, washed twice with PBS, and then co-cultured with purified CD8⁺ T cells for 72 h in a Transwell system (microglia in the lower chamber, CD8⁺ T cells in the upper insert). After co-culture, CD8⁺ T cells were harvested, washed with ice-cold FACS buffer (PBS containing 2% FBS), and stained for surface markers with fluorochrome-conjugated antibodies against CD3, CD8, and PD-1 for 30 min at 4 °C in the dark. Cells were washed, resuspended in FACS buffer, and analyzed on a flow cytometer. Lymphocytes were first gated by forward/side scatter, followed by CD3⁺CD8⁺ T-cell gating; PD-1 expression was then evaluated within the CD3⁺CD8⁺ population, and overlaid histogram plots were generated for the NCD+DMSO, Cho, and Cho+ATO groups

**Table S1. Clinical information of a total of 200 LUAD-BM patients from Huanhu Hospital Affiliated to Tianjin Medical University**

**[Excel]**

**Table S2.** **Antibody for Western blot, Immunofluorescence and Immunohistochemical Staining**

**[Excel]**

**Table S3. Primer sequences used in this study.**

**[Excel]**

**Table S4. shRNAs ans siRNAs sequences used in this study.**

**[Excel]**

**Table S5. Table S4. Prognostic factors for the interval from primary diagnosis to brain metastasis of LUAD-BM patients**

**[Word Cox analysis]**

**Table S6. Table S5. Prognostic factors for overall survival of LUAD-BM patients**

**[Word Cox analysis]**

**Table S7. Prognostic factors for intracranial progression-free survival of LUAD-BM patients**

**[Word Cox analysis]**

**Figure S1**


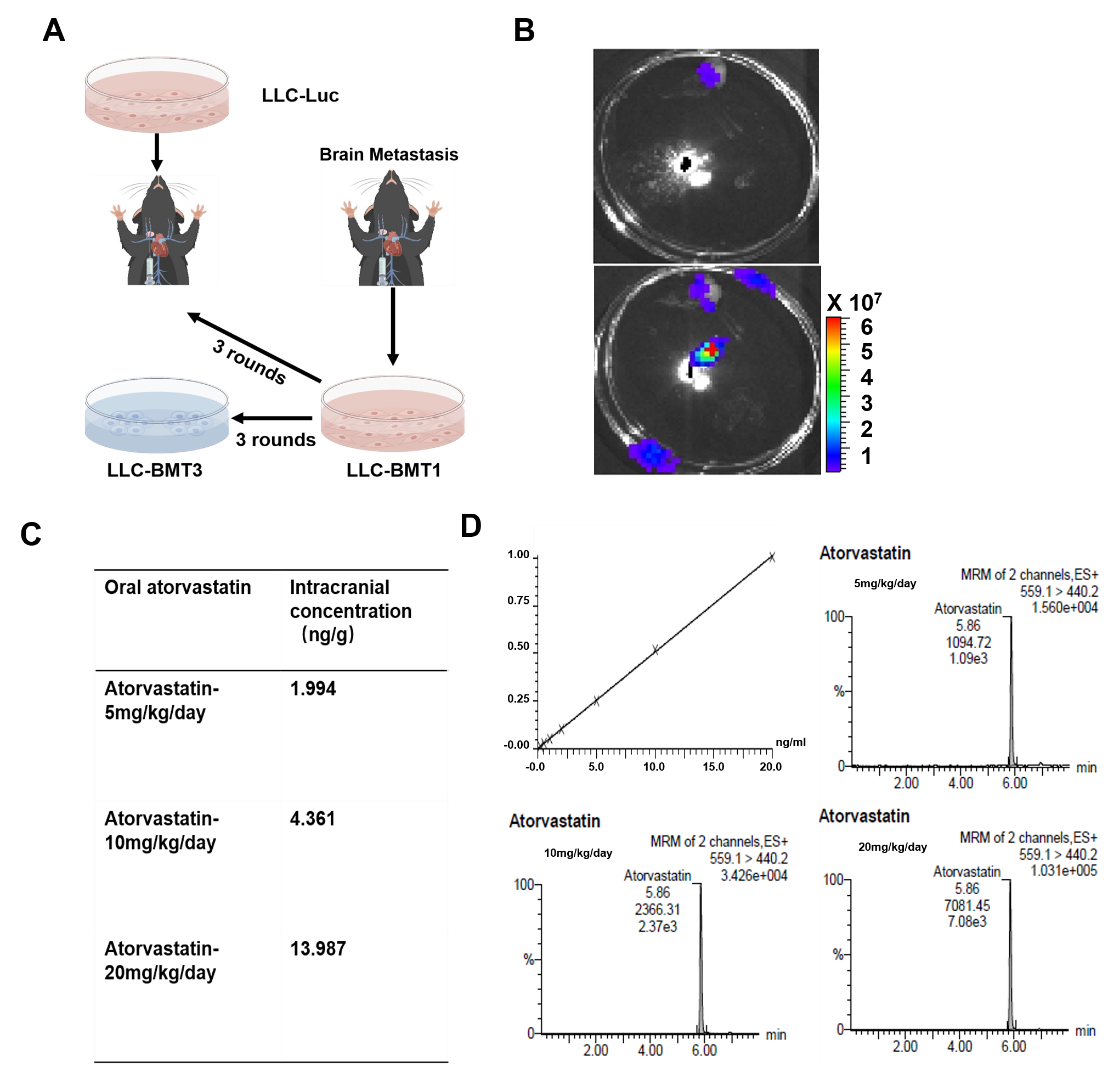


**Figure S1.** Construction of LUAD brain metastatic cell populations. A) Diagram showing the in vivo selection procedure of brain metastatic cell populations (BMT3) from LLC LUAD cancer cells. B) Cells from the brain metastatic sites (LLC-BMT1) are then isolated and passage through three rounds to generate LLC-BMT3 cells, which are also cultured in vitro. C) Quantification of ATO intracranial concentrations (ng/g) following oral gavage of 5, 10, or 20 mg/kg/day. Concentrations increased in a dose-dependent manner (1.994 ng/g, 4.361 ng/g, and 13.987 ng/g, respectively). D) The drug concentrations of ATO standards were detected by LC-MS for acquisition of the standard curve.

**Figure S2**


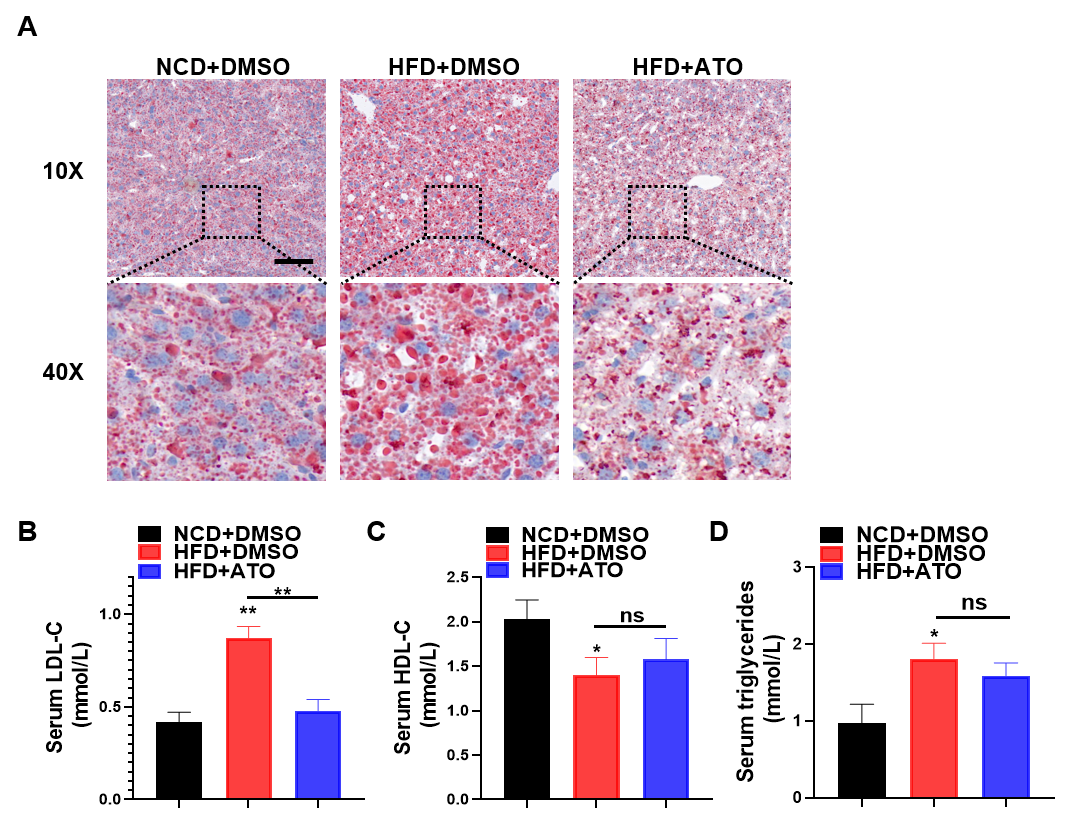


**Figure S2.** High-fat diet induces hepatic steatosis and dyslipidemia in mice. A) Photomicrograph of livers stained with oil red O (scale bar, 300 μm). B-D) At week 12, LLC-BMT3 cells were injected via tail vein. Effects of high-fat diet on serum LDL-C (B), serum HDL-C (C) and serum triglycerides (D). ***P* < 0.01, **P* < 0.05, ns, not significant (one-way ANOVA).

**Figure S3**


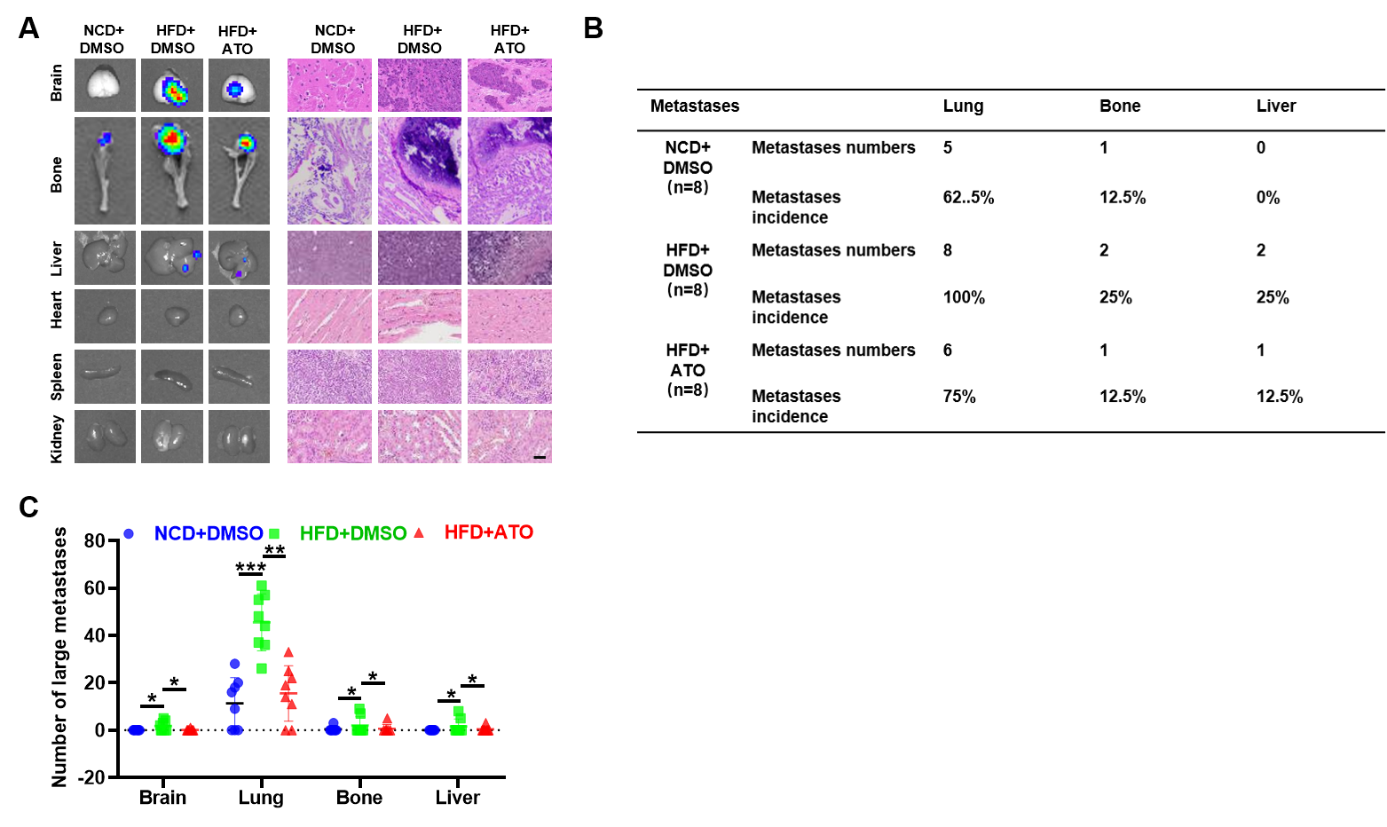


**Figure S3.** hypercholesterolemia modestly increases systemic metastatic dissemination.

A) Representative bioluminescence images and H&E staining of major organs (brain, bone, liver, heart, spleen and kidney) harvested from NCD, HFD, and HFD+ATO groups following tail-vein injection of LLC-BMT3 cells (Scale bar, 200 μm). B) Quantification of the percentages of mice that developed lung, bone and liver metastases after LLC-BMT3 cells were injected via tail vein. C) Quantification of metastatic nodule numbers in each organ. ****P* < 0.001, ***P* < 0.01, **P* < 0.05, ns, not significant (one-way ANOVA).

**Figure S4**


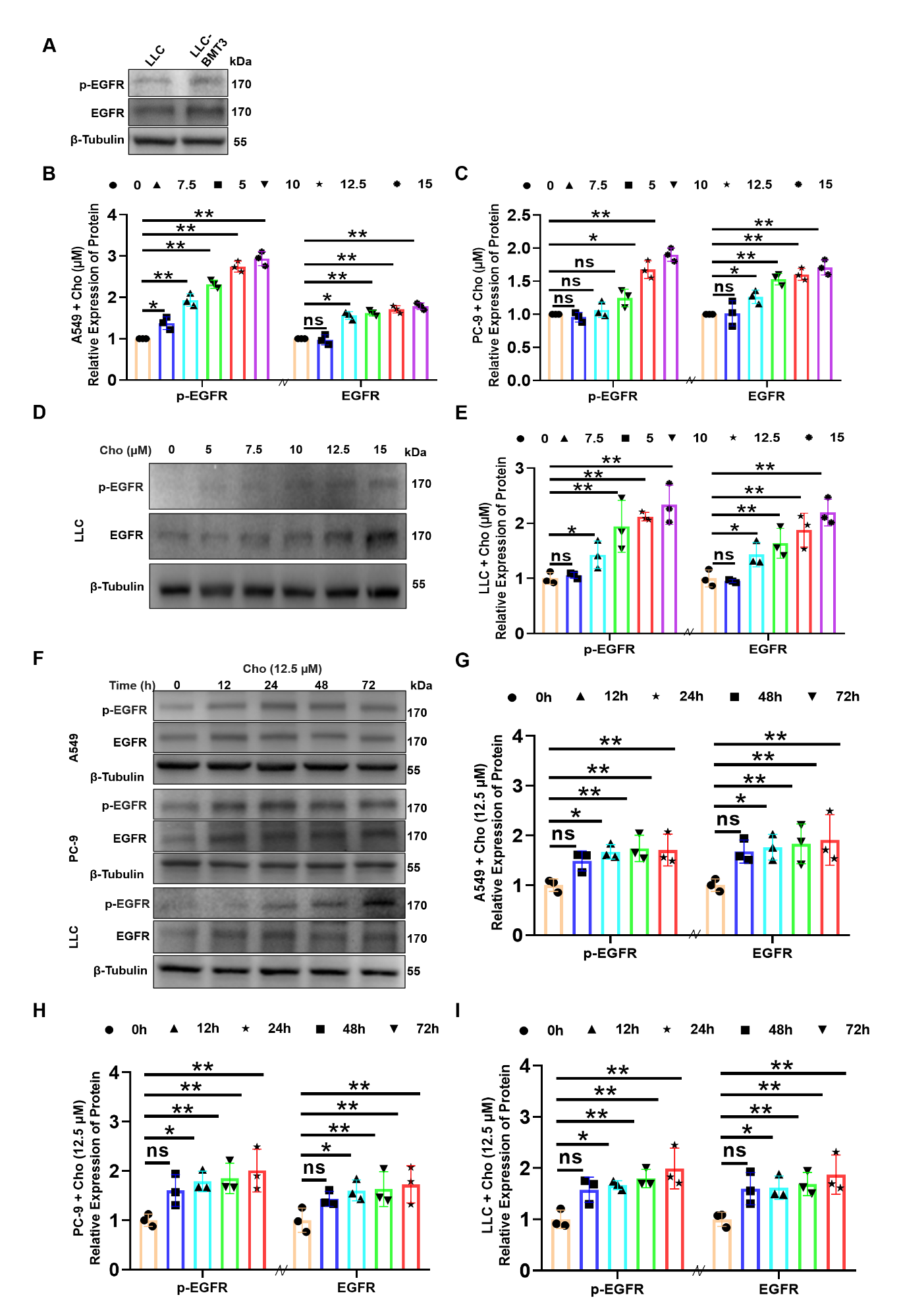


**Figure S4.** Cholesterol regulates p-EGFR and EGFR expression in lung cancer cells in a dose- and time-dependent manner. A) Western blotting analysis of EGFR, p-EGFR and β-Tubulin in LLC cells and LLC-BMT3 cells. B-C) A549 (B) and PC-9 (C) cells were treated with increasing concentrations of cholesterol (0, 5, 7.5, 10, 12.5, and 15 μmol/L) for 48 hours. Western blotting was performed to assess p-EGFR and total EGFR protein levels. β-Tubulin served as a loading control. Quantitative analysis is shown. **P* < 0.05, ***P* < 0.01, ns, not significant (one-way ANOVA). D-E) LLC cells were treated with the same cholesterol concentrations for 48 hours. Western blotting results (D) and corresponding densitometric quantification (E) show p-EGFR and EGFR levels, with β-Tubulin as the internal control. **P* < 0.05, ***P* < 0.01, ns, not significant (one-way ANOVA). F-I) A549, PC-9, and LLC cells were treated by 12.5 ng/mL Cho at different time points. Western blotting analysis was performed to detect the expression levels of p-EGFR, EGFR and β-Tubulin (F). Quantitative analyses are presented (G-I). **P* < 0.05, ***P* < 0.01, ns, not significant (one-way ANOVA).

**Figure S5**


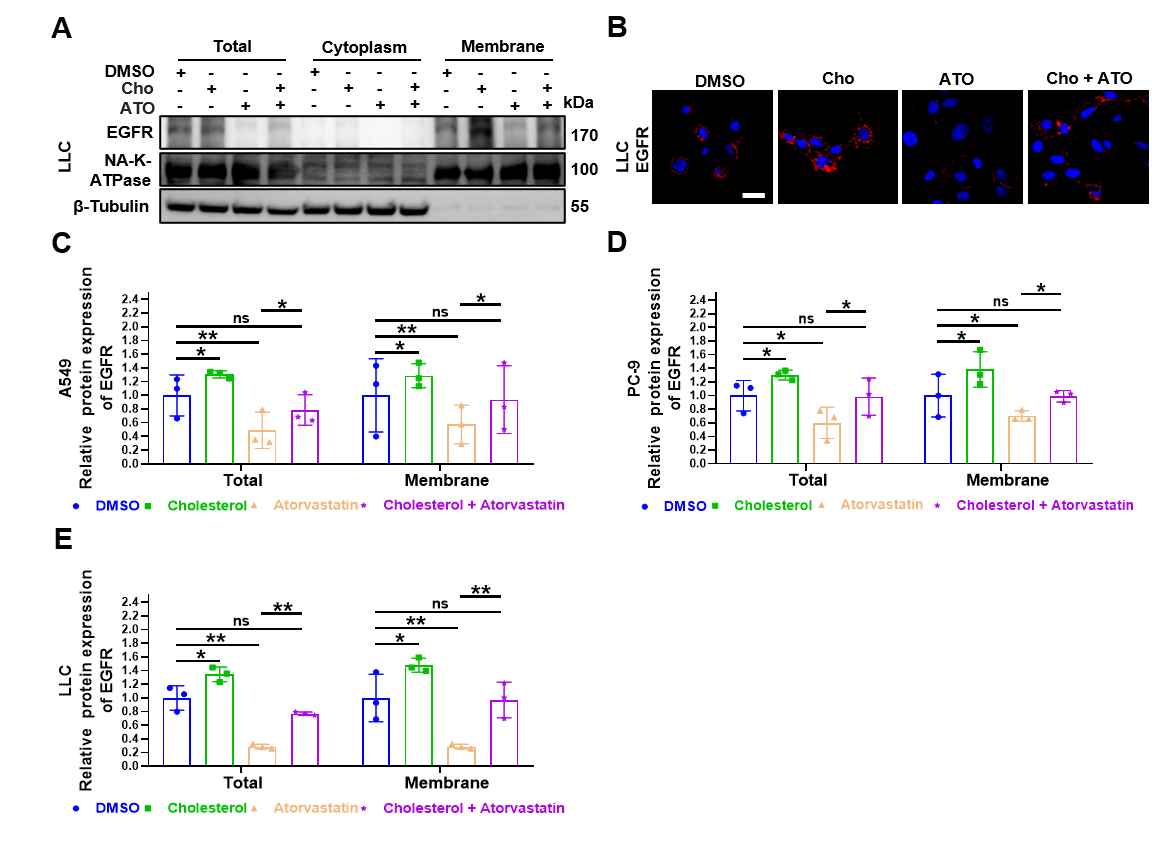


**Figure S5.** Cholesterol regulates the membrane-localized levels of EGFR in LLC cells. A) Cytosolic and membrane fractions were isolated from LLC cells treated with DMSO, 12.5 μmol/L Cho, ATO, or ATO combined with Cho. Western blotting was performed to detect p-EGFR and total EGFR levels in each compartment. Na-K-ATPase and β-Tubulin were used as membrane and cytosolic markers, respectively. B) Immunofluorescence staining of EGFR in LLC cells treated with DMSO, 12.5 μmol/L Cho, ATO, or the combination of ATO and Cho (scale bar, 60 μm). C-E) Cytosolic and membrane fractions of A549 (C), PC-9 (D), and LLC (E) cells treated under the same conditions (DMSO, Cho, ATO, or ATO + Cho) were subjected to western blotting to quantitatively analyze the expression and distribution of p-EGFR and EGFR. β-Tubulin served as a loading control. **P* < 0.05, ***P* < 0.01, ns, not significant (one-way ANOVA).

**Figure S6**


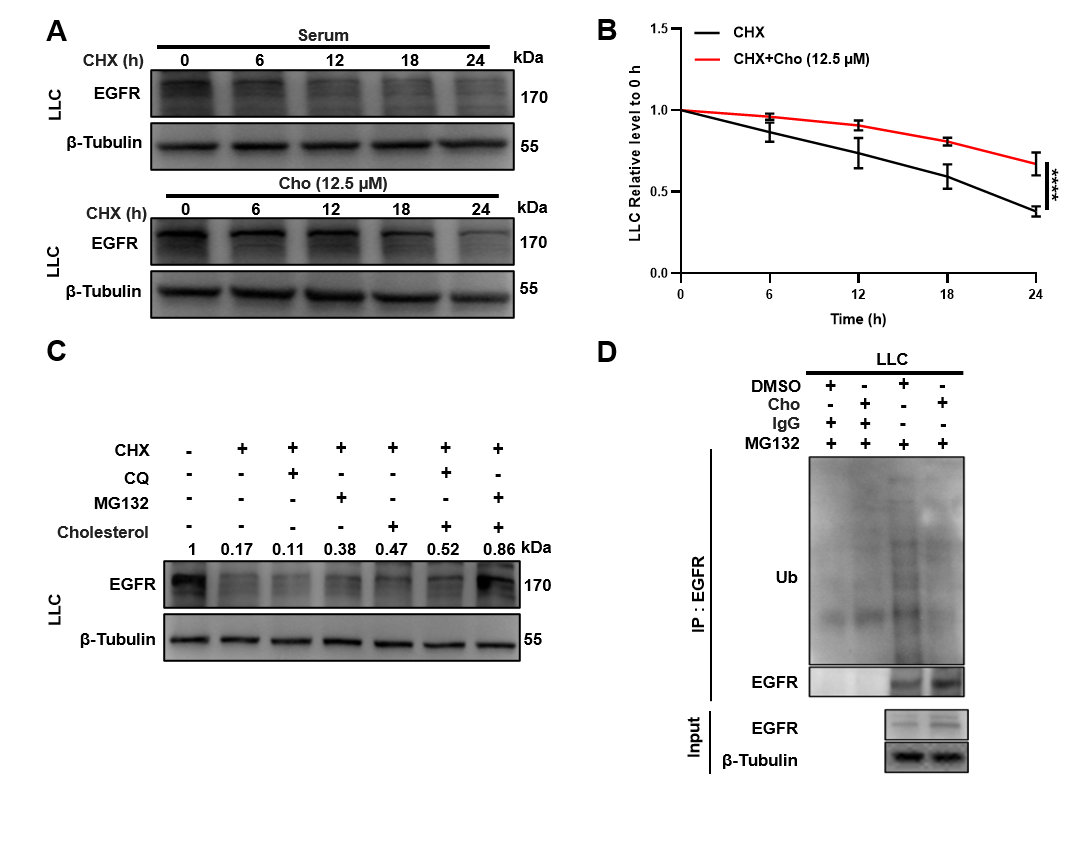


**Figure S6.** Cholesterol affected the membrane-localized levels of EGFR by reduced EGFR ubiquitination. A-B) Western blot analysis (A) and quantitative assessment (B) of EGFR degradation in LLC cells treated with CHX (10 μg/mL) alone or in combination with Cho (12.5 μmol/L) for 48 h. *****P* < 0.0001 (two-way ANOVA). C) Western blotting analysis of EGFR in LLC cells treated with CHX (10 μg/mL), MG132 (10 μM), CQ (25μM), or Cho (12.5 µM) for 48 h, respectively. D) Co-IP assays were conducted with anti-EGFR antibody in LLC cells treated with DMSO or Cho (12.5 µM) for 48 h, respectively, followed by immunoblotting with ubiquitin.

**Figure S7**


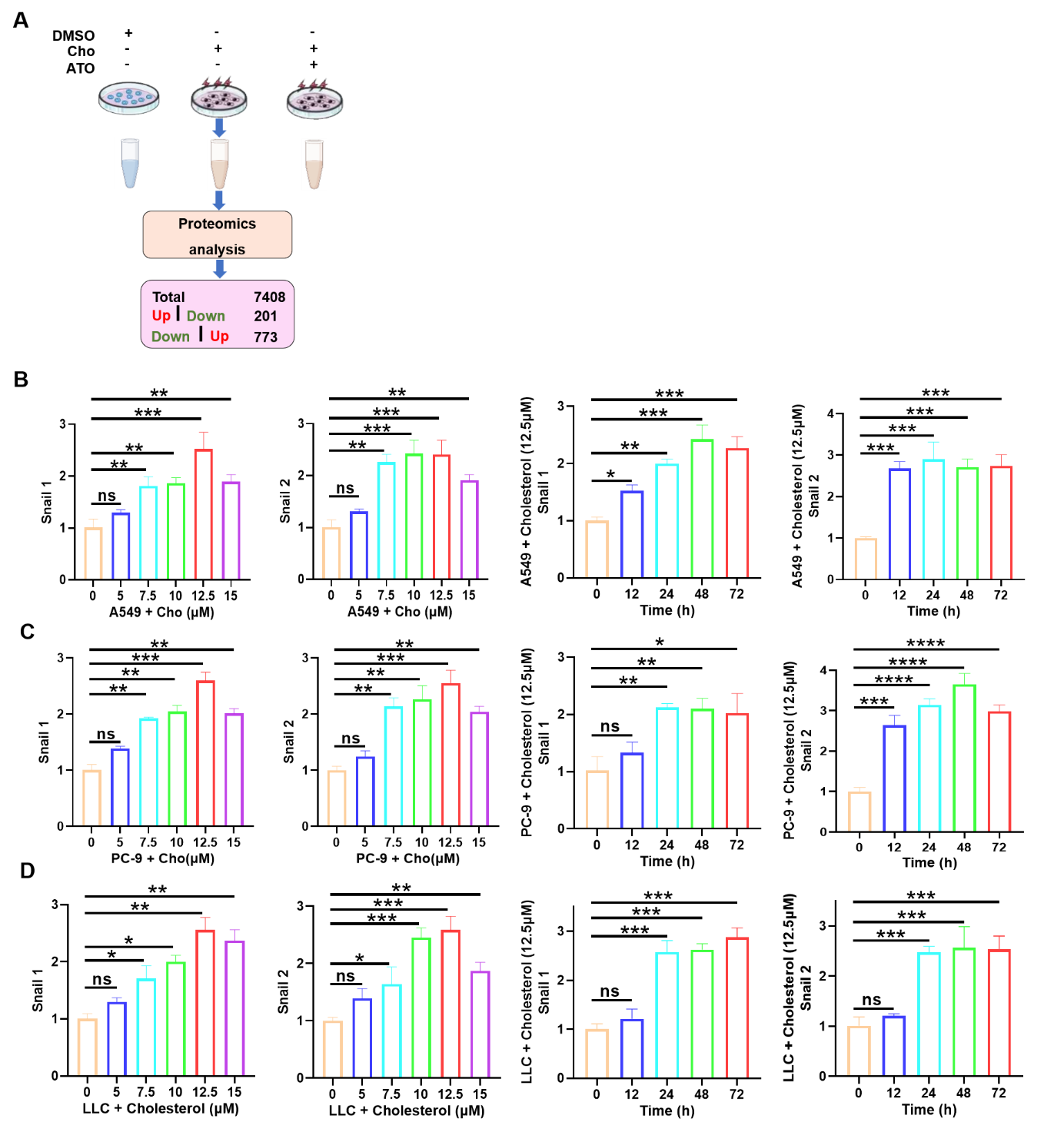


**Figure S7.** Cholesterol can upregulate the expression of EMT related transcription factors in LUAD. A) Schematic representation of the liquid chromatography-mass spectrometry (LC-MS) workflow used to analyze metabolic alterations in PC-9 cells treated with DMSO, cholesterol (Cho, 12.5 μmol/L), or atorvastatin (ATO) combined with Cho.B-D) Validation of changes in the expression of EMT-related genes in A549 (B), PC-9 (C) and LLC (D) cells were treated with 0, 5, 7.5, 10, 12.5, and 15 μmol/L Cho for 48 h or 12.5 ng/mL Cho at different time points using RT-qPCR. **P* < 0.05, ***P* < 0.01, ****P* < 0.001, *****P* < 0.0001, ns, not significant (one-way ANOVA).

**Figure S8**


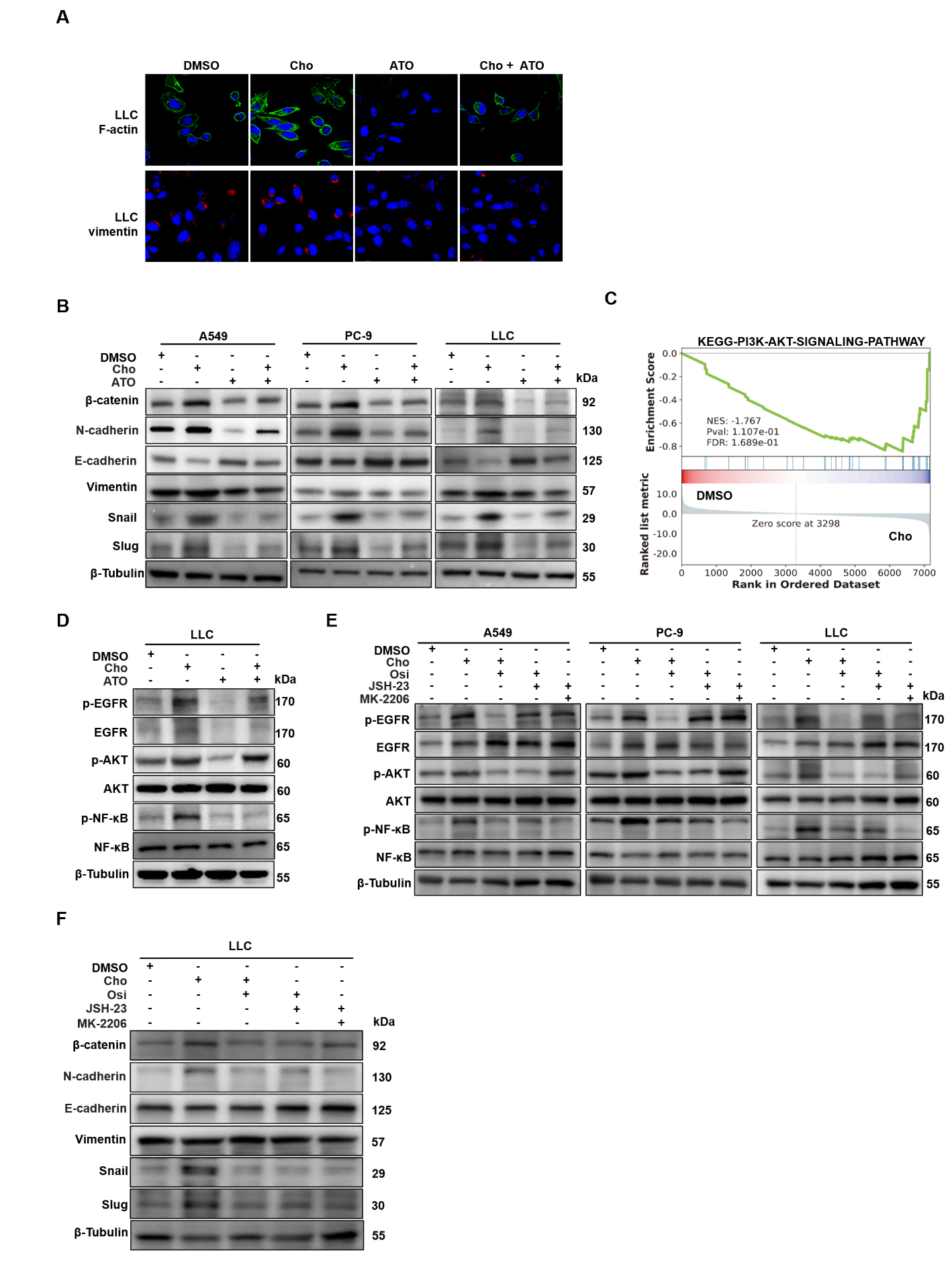


**Figure S8.** Cholesterol can upregulate the expression of EMT related transcription factors in LUAD. A) Immunofluorescence assays of F-actin and Vimentin in LLC cells treated with DMSO, 12.5 μmol/L Cho, ATO or ATO plus 12.5 μmol/L Cho (scale bar, 60 μm). B) Western blotting to check the expression levels of β-catenin, N-cadherin, E-cadherin, Snail, Slug, Vimentin and β-Tubulin in A549, PC-9 and LLC cells treated with DMSO, Cho, ATO or ATO plus Cho. C) PC-9 cells were treated with DMSO, 12.5 μmol/L Cho or ATO plus 12.5 μmol/L Cho, followed by mass spectrometry-based proteomic analysis (n = 3 biological replicates per group). Gene set enrichment analysis (GSEA) identified significant enrichment in PI3K-AKT-SIGNALING pathways. D) Western blot of p-EGFR, EGFR, p-AKT, AKT, p-NF-κB, NF-κB and β-Tubulin in LLC cells treated with DMSO, 12.5 μmol/L Cho, ATO or ATO plus 12.5 μmol/L Cho. E) Western blotting to check the expression levels of p-EGFR, EGFR, p-AKT, AKT, p-NF-κB, NF-κB and β-Tubulin in LLC cells treated with DMSO, 12.5 μmol/L Cho, Osi, MK-2206 or JSH-23. F) Western blot analysis of EMT markers in LLC cells treated with DMSO, Cho, Osi, MK-2206, or JSH-23.

**Figure S9**


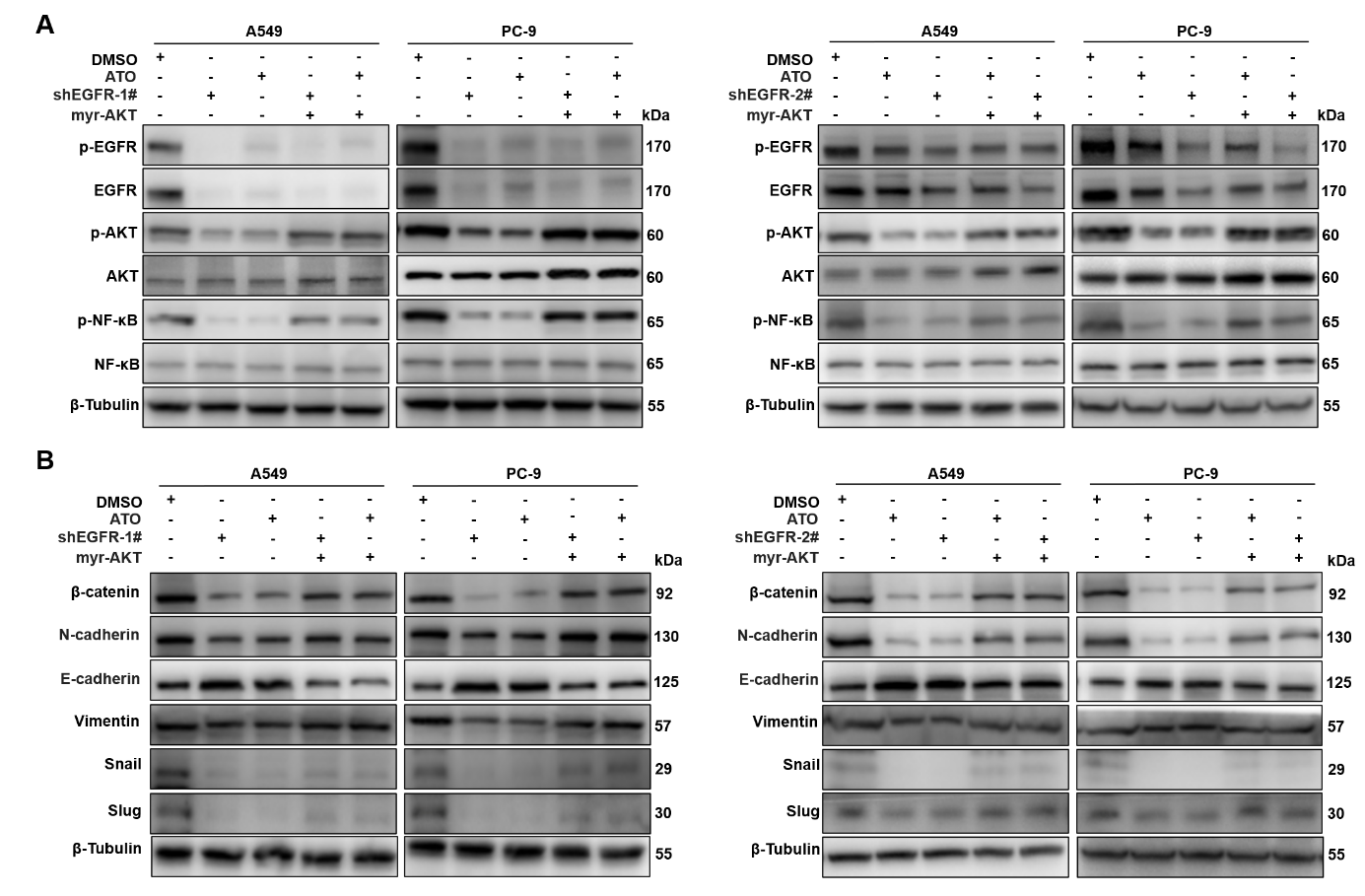


**Figure S9.** EGFR knockdown and AKT reactivation clarify the causal role of the EGFR–AKT axis in cholesterol-induced EMT. A, B) Western blot analyses showing the effects of atorvastatin (ATO), EGFR knockdown (shEGFR-1# and shEGFR-2#), and constitutively active AKT (myr-AKT) expression on downstream signaling (A) and EMT marker (B) expression in LUAD cells.

**Figure S10**

**
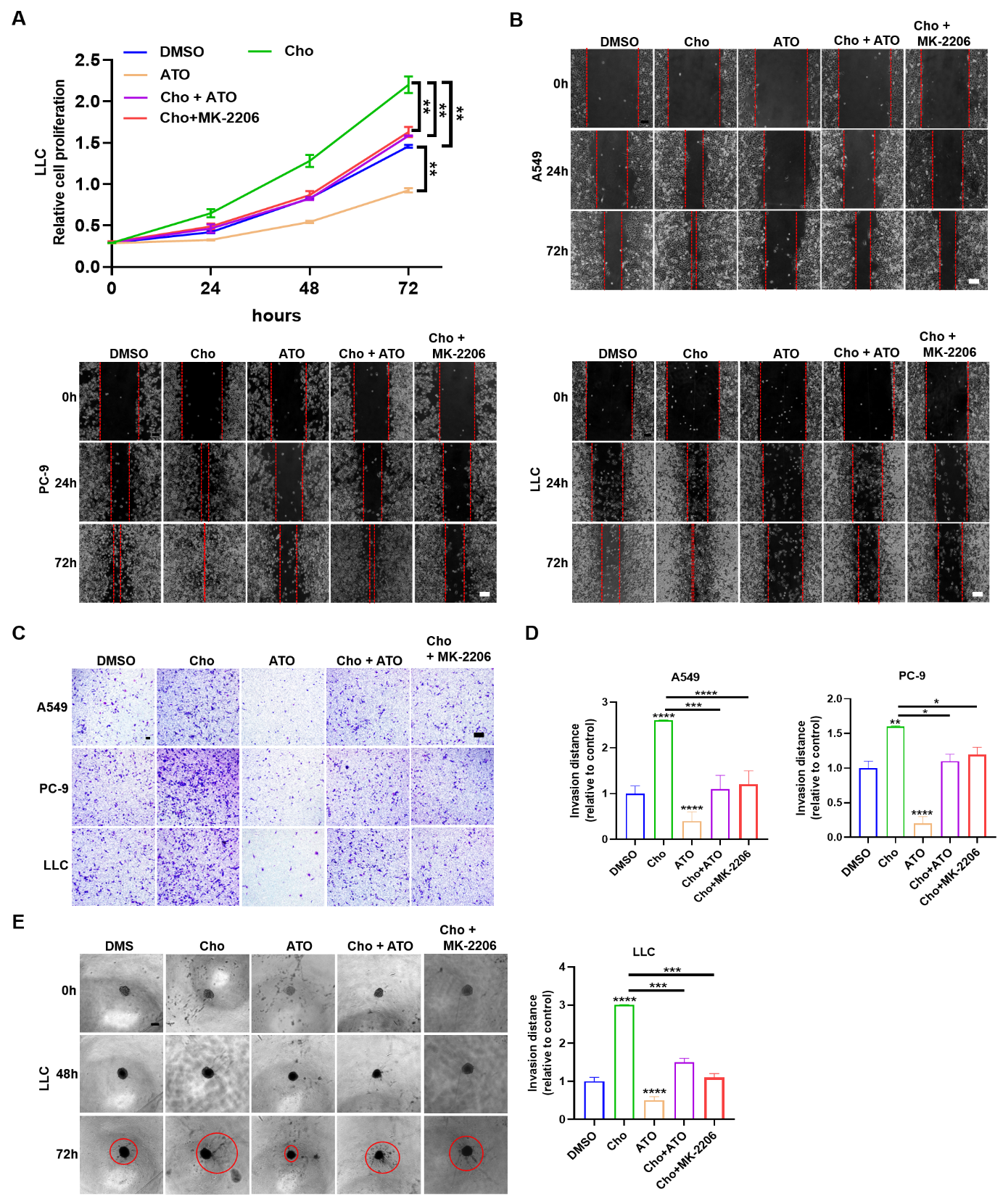
**

**Figure S10.** Cholesterol promotes the EMT process by activating EGFR/AKT/NF-κB/β-catenin pathway in LUAD. A) Growth curves for LLC cells treated with DMSO, Cho, Osi, MK-2206. **P* < 0.05, ***P* < 0.01, ****P* < 0.001, *****P* < 0.0001 (two-way ANOVA). B, C) Wound healing analysis (B) and transwell assays (scale bar, 300 μm) (C) for A549, PC-9 and LLC cells treated with DMSO, 12.5 μmol/L Cho, Osi, MK-2206. (scale bar, 300 μm). D) Quantification of 3D tumor sphere invasion assays for A549 and PC-9 cells treated with DMSO, 12.5 μmol/L Cho, Osi, MK-2206 (scale bar, 300 μm), **P* < 0.05, ***P* < 0.01, ****P* < 0.001, *****P* < 0.0001 one-way ANOVA). E) Brightfield images of 3D tumor sphere invasion assays and quantification for LLC cells treated with DMSO, 12.5 μmol/L Cho, Osi, MK-2206 (scale bar, 300 μm), ****P* < 0.001, *****P* < 0.0001 one-way ANOVA).

**Figure S11**


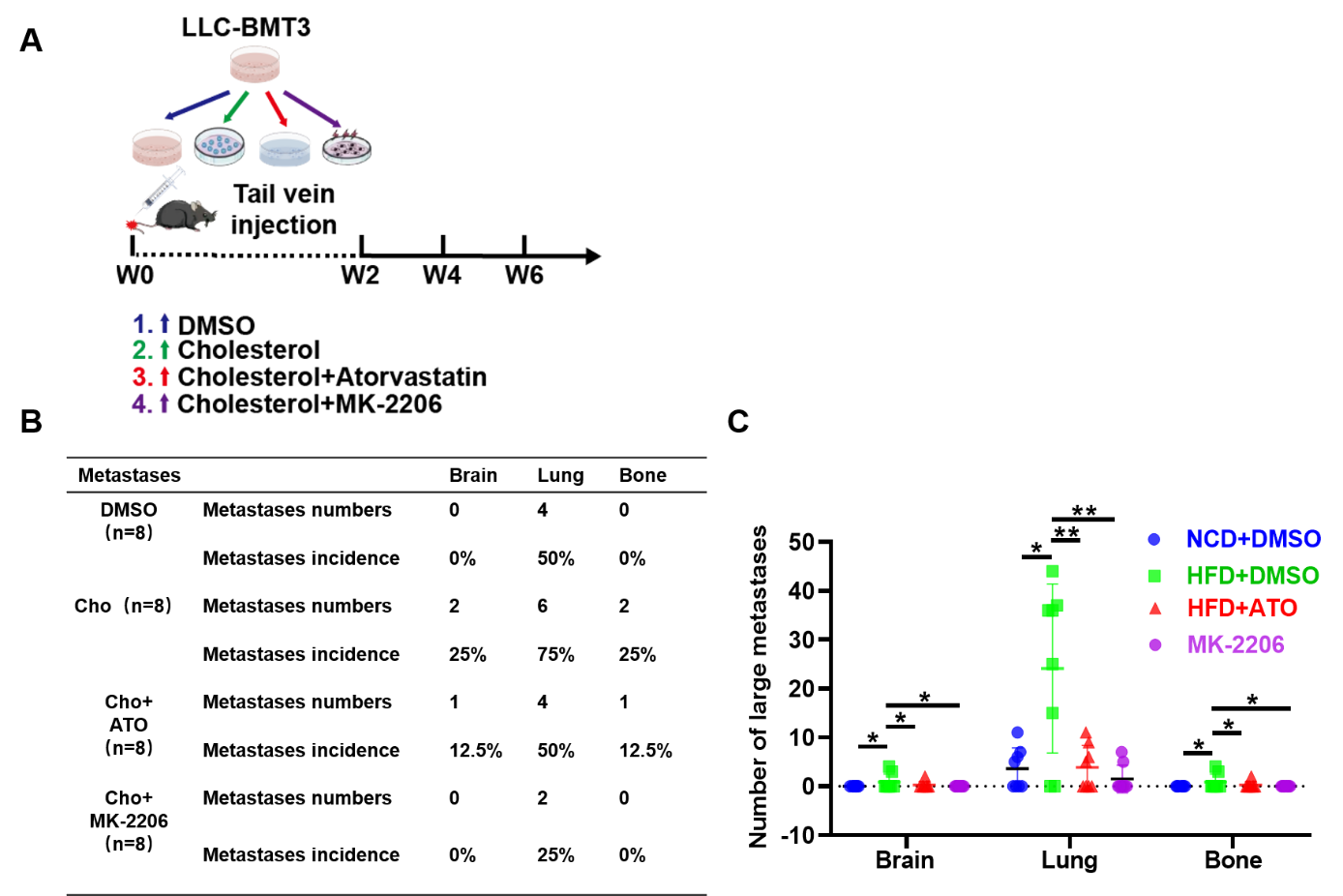


**Figure S11.** Cholesterol promotes the proliferation, invasion, and metastasis of *in situ* LUAD cells by modulating the EGFR/AKT/NF-κB pathway. A) Schematic representation of the in vivo experimental design: LLC-BMT3 cells were pretreated with DMSO, cholesterol (Cho, 12.5 μmol/L), atorvastatin (ATO) plus Cho, or MK-2206 plus Cho for 48 h, and then injected via the tail vein into C57BL/6J male mice. B) Quantification of the percentages of mice that developed brain, lung and bone metastases after LLC-BMT3 cells were injected via tail vein. C) Quantification of metastatic nodule numbers in each organ. ***P* < 0.01, **P* < 0.05, ns, not significant (one-way ANOVA).

**Figure S12**


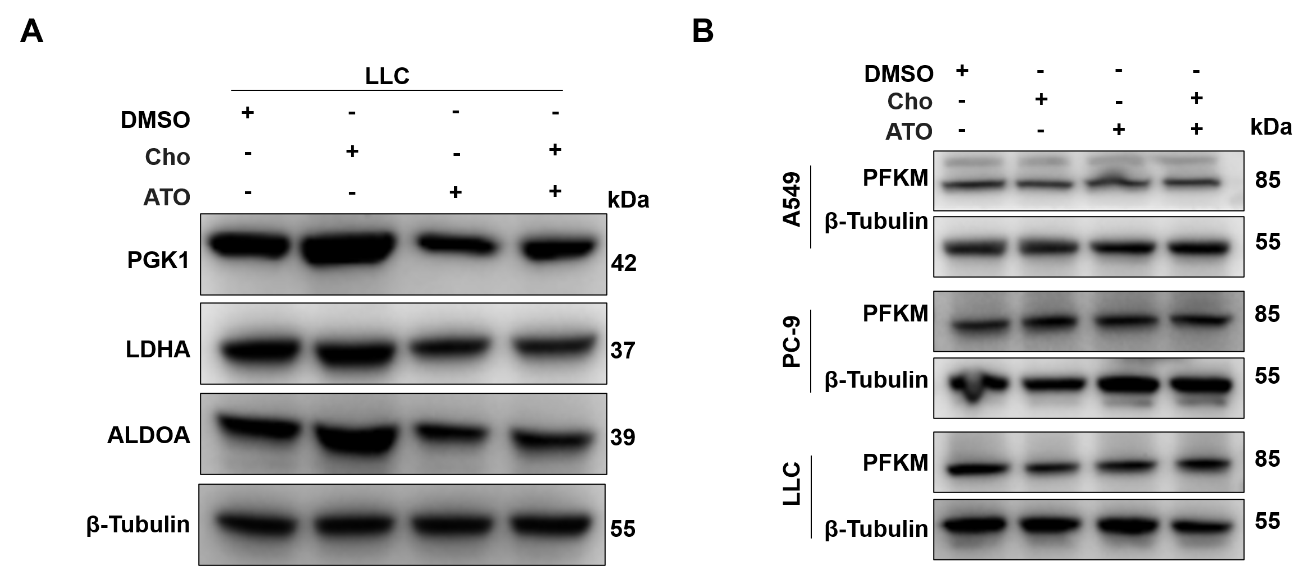


**Figure S12.** Cholesterol modulates the expression of glycolysis-related enzymes. A) Western blot analysis of the glycolytic enzymes PGK1, LDHA, ALDOA, and β-Tubulin in LLC cells following treatment with DMSO, 12.5 μmol/L Cho, ATO or ATO plus 12.5 μmol/L Cho. B) Expression levels of PFKM, were examined by western blotting in A549, PC-9, and LLC cells treated under the same conditions as above. β-Tubulin was used as a loading control.

**Figure S13**


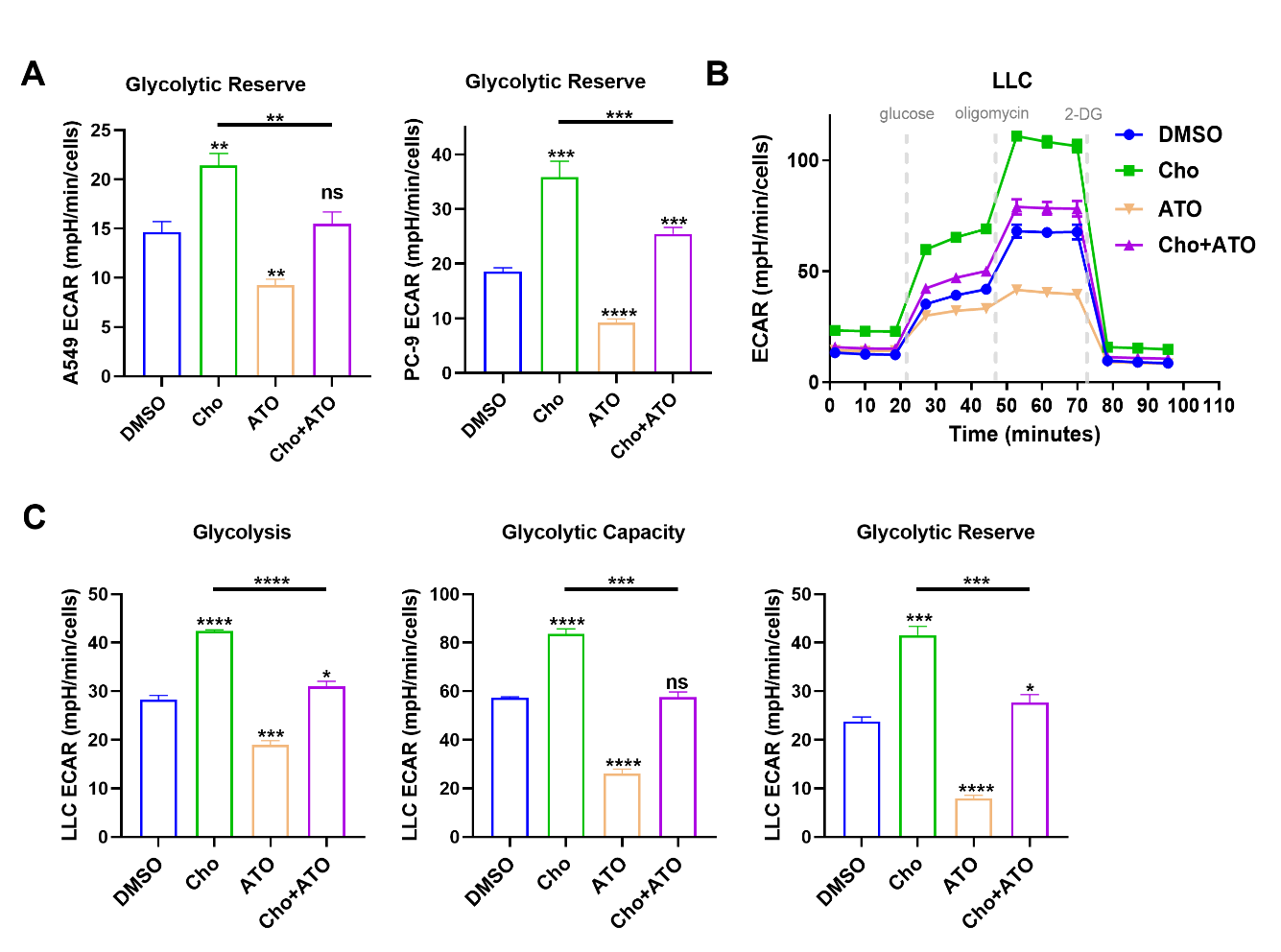


**Figure S13.** Cholesterol reprograms the energy metabolism of LUAD cells. A) A549 and PC-9 cells were treated with DMSO, cholesterol (Cho, 12.5 μmol/L), or MK-2206 combined with Cho for 48 h. Glycolytic reserve was assessed using the Seahorse XF Glycolytic Rate Assay. ***P* < 0.01, ****P* < 0.001, *****P* < 0.0001, ns, not significant (one-way ANOVA). The glycolytic functions. B-C) LLC cells were treated with DMSO, 12.5 μmol/L Cho, ATO or ATO plus 12.5 μmol/L Cho for 48 h. Glycolytic function was evaluated using the Seahorse XF Glycolytic Rate Assay. (B) Time course of extracellular acidification rate (ECAR). (C) Quantification of glycolysis, glycolytic capacity, and glycolytic reserve (C). **P* < 0.05, ****P* < 0.001, *****P* < 0.0001, ns, not significant (one-way ANOVA).

**Figure S14**


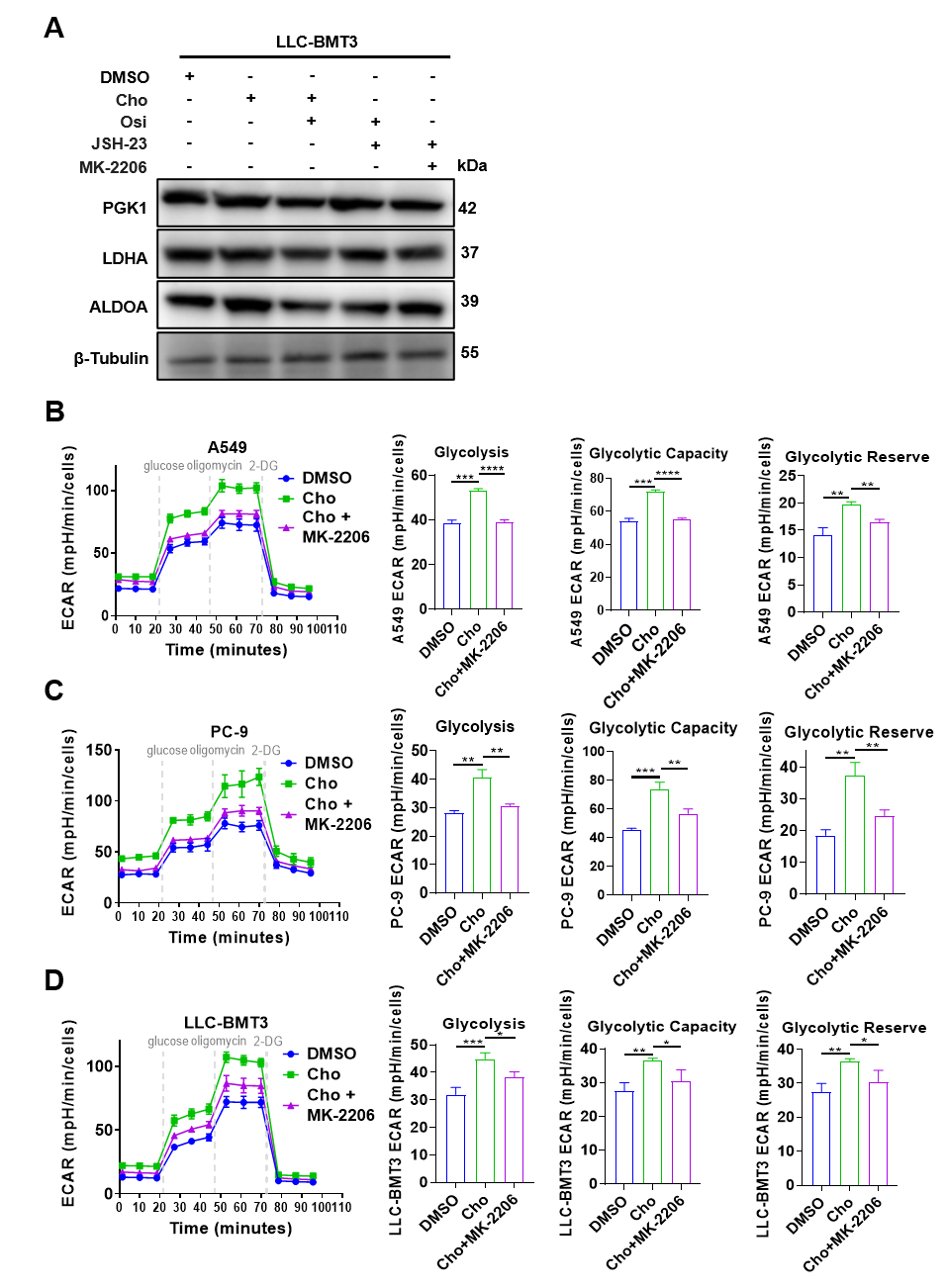


**Figure S14.** Cholesterol reshapes energy metabolism in LUAD via AKT signaling inhibition. A) Western blotting to check the expression levels of PGK1, LDHA, ALDOA, and β-Tubulin in LLC-BMT3 cells treated with DMSO, 12.5 μmol/L Cho, Osi, MK-2206 or JSH-23. B-D) A549 (B), PC-9 (C) and LLC-BMT3 (D) cells were treated with DMSO, Cho, or MK-2206 plus Cho for 48 h. Glycolytic function was assessed by Seahorse XF Glycolytic Rate Assay, including extracellular acidification rate (ECAR) over time, glycolytic activity, glycolytic capacity, and glycolytic reserve. **P* < 0.05, ***P* < 0.01, ****P* < 0.001, ****P < 0.0001 (one-way ANOVA).

**Figure S15**

**
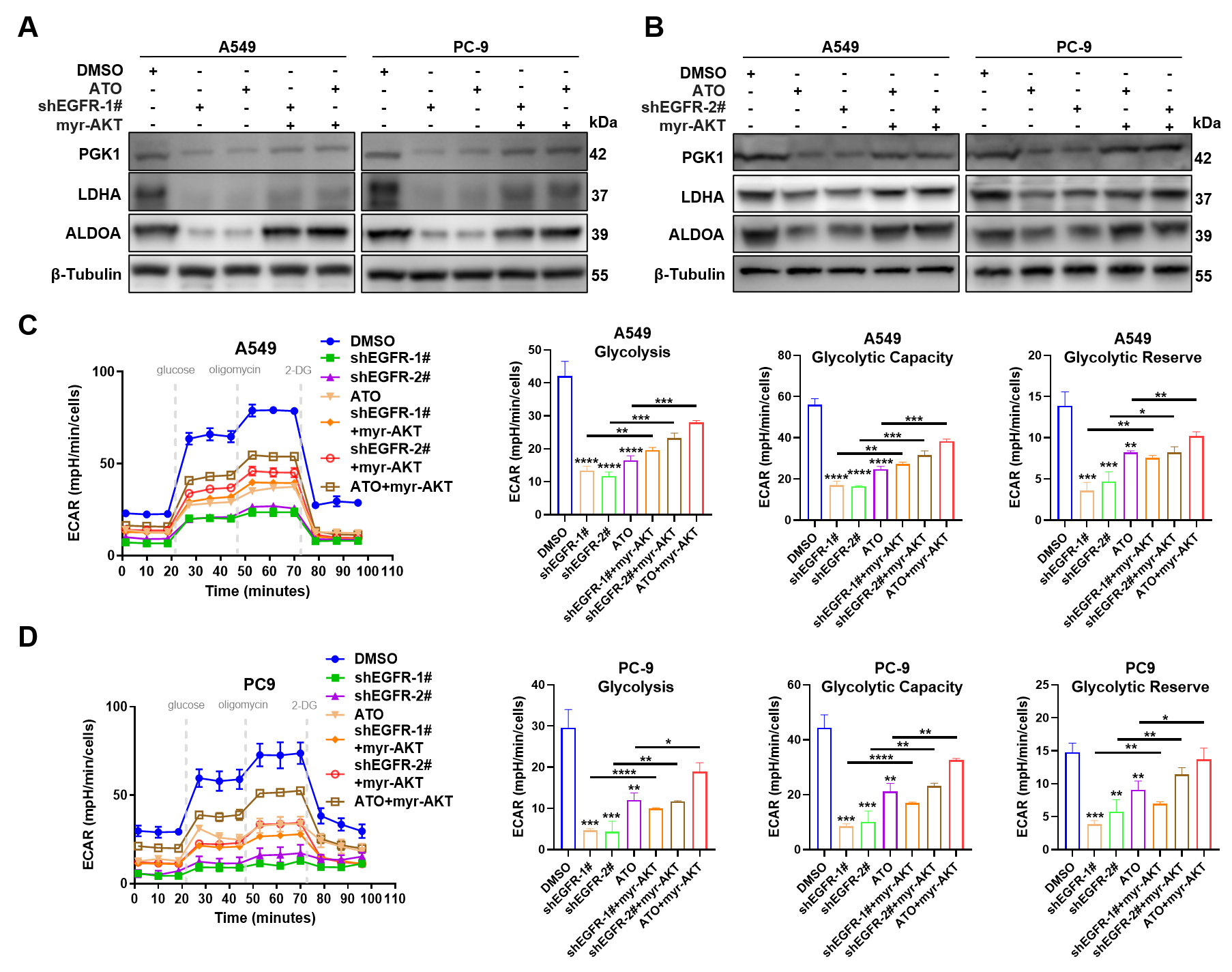
**

**Figure S15.** EGFR knockdown and AKT reactivation clarify the causal role of the EGFR–AKT axis in cholesterol-induced energy metabolism. A) Western blot analyses showing the effects of atorvastatin (ATO), EGFR knockdown (shEGFR-1# and shEGFR-2#), and constitutively active AKT (myr-AKT) expression on downstream PGK1, LDHA, ALDOA, and β-Tubulin expression in LUAD cells. B) Glycolytic function was assessed by Seahorse XF Glycolytic Rate Assay, including extracellular acidification rate (ECAR) over time, glycolytic activity, glycolytic capacity, and glycolytic reserve. **P* < 0.05, ***P* < 0.01, ****P* < 0.001, ****P < 0.0001 (one-way ANOVA).

**Figure S16**

**
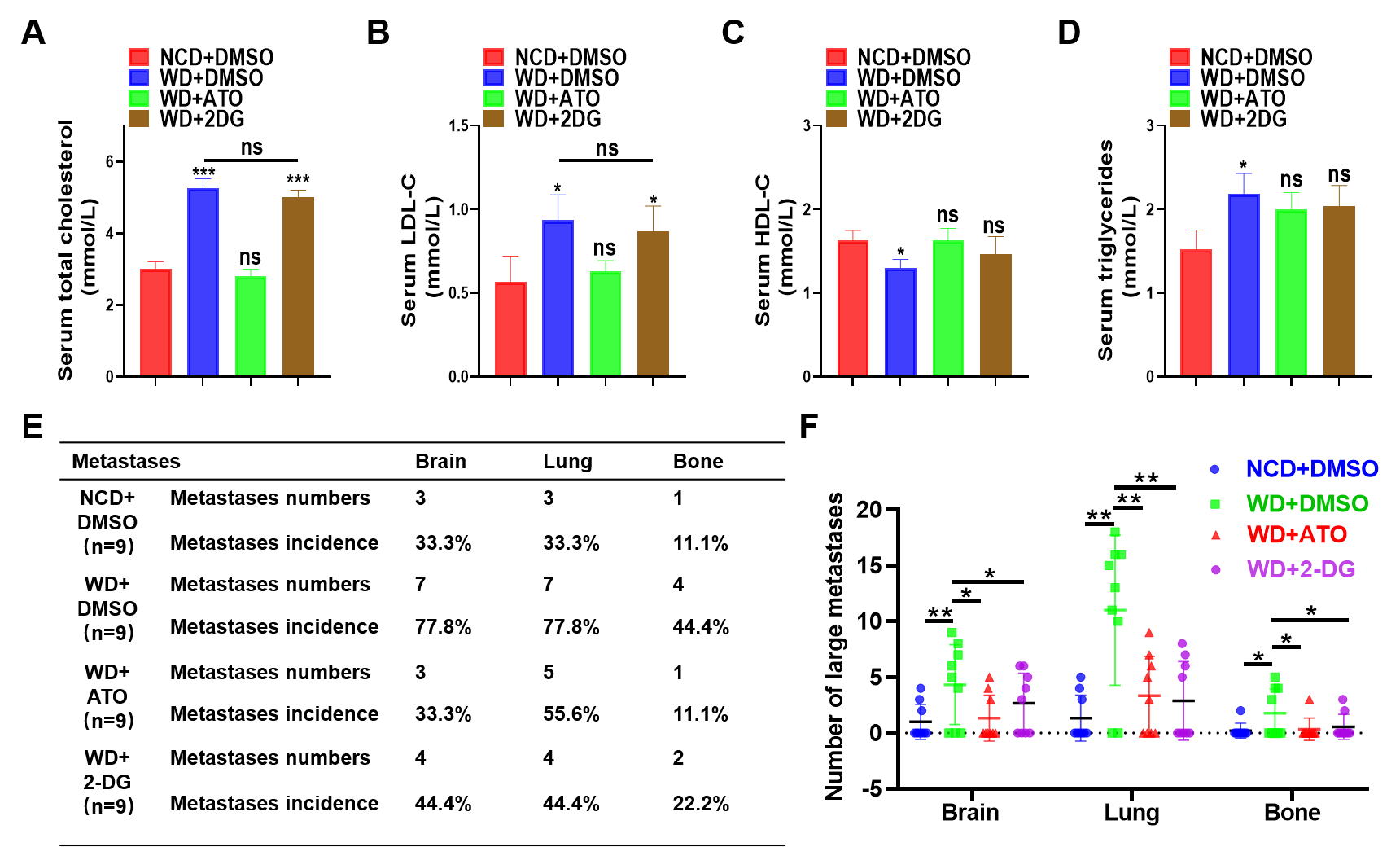
**

**Figure S16.** Cholesterol promotes the brain metastasis of PC-9 cells by modulating the energy metabolism. A-D) At week 6, LPC-9 cells were injected via tail vein. Effects of high-fat diet on serum TC (A), LDL-C (B), serum HDL-C (C) and serum triglycerides (D). **P* < 0.05, ****P* < 0.001, ns, not significant (one-way ANOVA). E) Quantification of the percentages of mice that developed brain, lung and bone metastases after PC-9 cells were injected via intracardiac injection. F) Quantification of metastatic nodule numbers in each organ. **P* < 0.05, ***P* < 0.01, ns, not significant (one-way ANOVA).

**Figure S17**

**
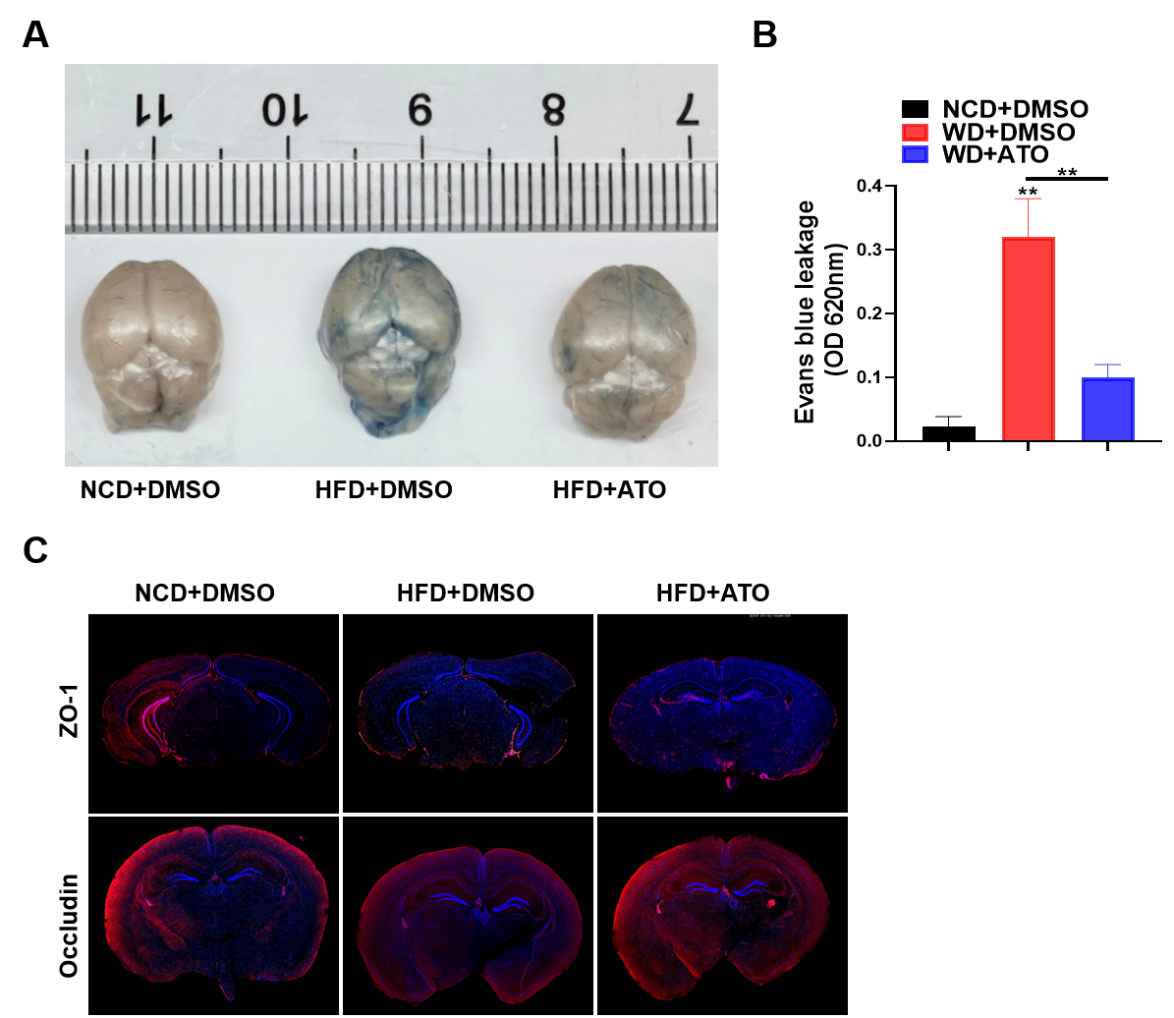
**

**Figure S17.** Cholesterol impairs blood–brain barrier (BBB) integrity, with minimal effects on ZO-1/Occludin. A) Representative images of Evans Blue extravasation in brains from mice fed a NCD, HFD, or HFD+ATO. B) Quantification of Evans Blue content in brain tissues across treatment groups. ***P* < 0.01 (one-way ANOVA). C) Immunofluorescence staining of brain sections from C57BL/6J male mice fed NCD, HFD or HFD were orally administered ATO (10 mg/kg/day) for 12 weeks, ZO-1 and Occludin (red) expression was stained.

**Figure S18**

**
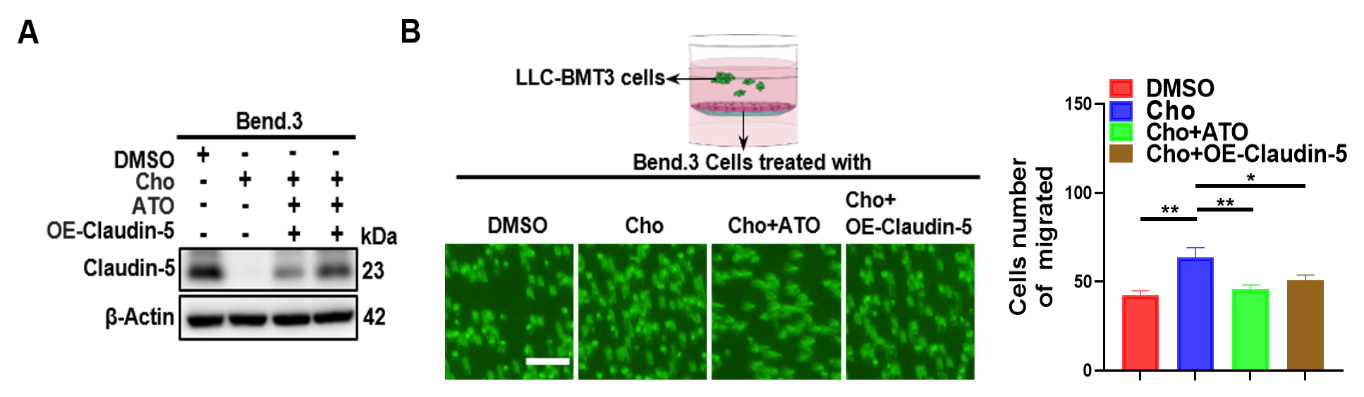
**

**Figure S18.** Claudin-5 overexpression mitigates cholesterol-induced BBB disruption and tumor cell trans-endothelial migration. A) Western blot analysis showing that Claudin-5 overexpression rescues cholesterol-induced downregulation of Claudin-5 protein in Bend.3 endothelial cells, whereas atorvastatin (ATO) further restores Claudin-5 expression under cholesterol exposure. B) Representative fluorescence images showing GFP-positive tumor cells that successfully crossed the BBB layer under different conditions. Cholesterol markedly increased trans-endothelial tumor cell migration, whereas Claudin-5 overexpression significantly reduced cell passage across the endothelial barrier. Scale bar, 50 μm. Quantification of GFP-positive tumor cells migrating through the endothelial monolayer. ***P* < 0.01, ****P* < 0.001 (one-way ANOVA).

**Figure S19**

**
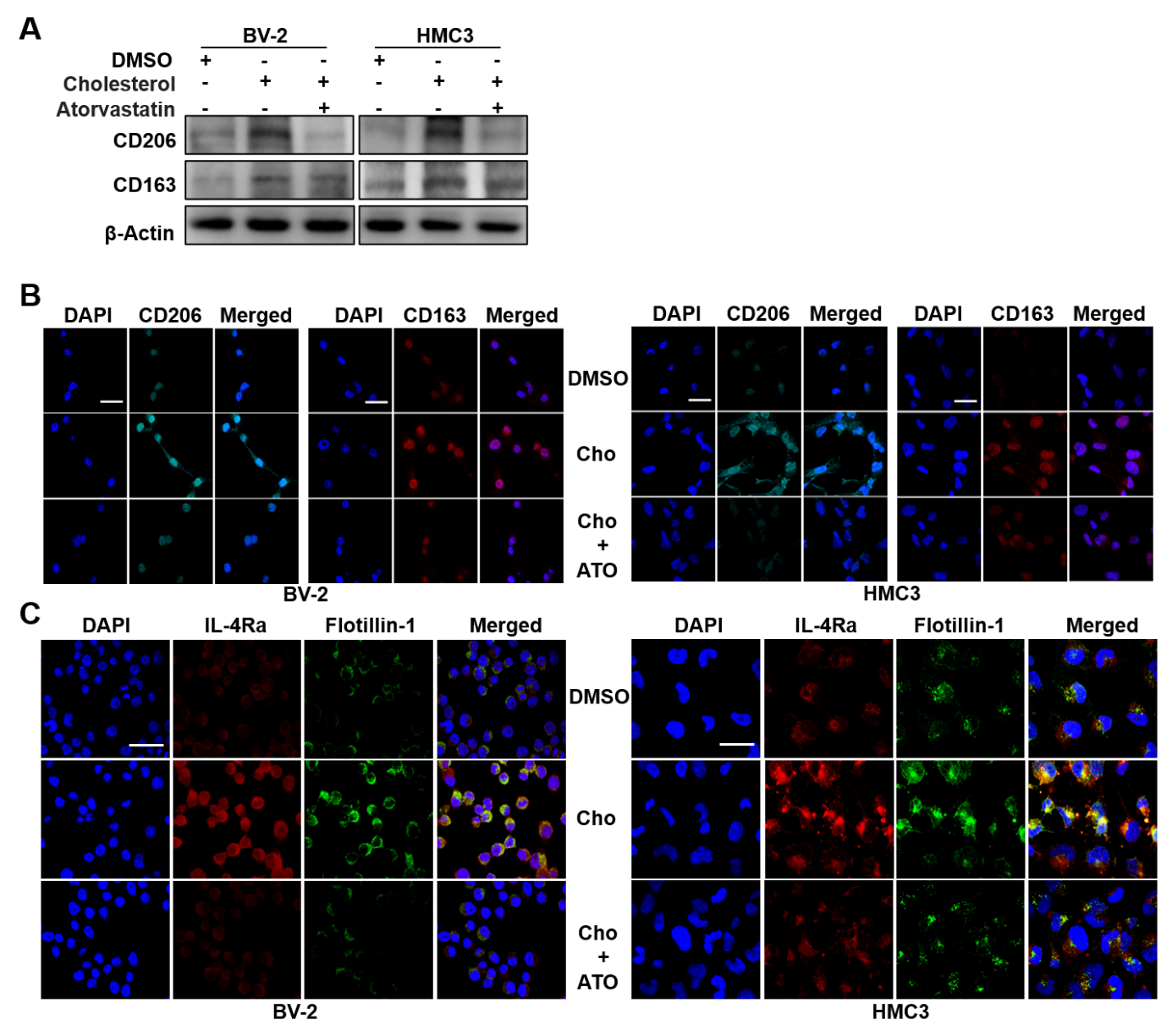
**

**Figure S19.** Cholesterol induces M2 microglial polarization by promoting IL-4Rα recruitment to lipid rafts. A-B) Western blotting (A) and Immunofluorescence assays (B) to check the expression levels of CD206 (cyan), CD163 (red) and β-Actin in BV-2 and HMC3 cells treated with DMSO, Cho (12.5 μmol/L) or Cho plus ATO (12.5 μmol/L) (scale bar, 60 μm). C) Immunofluorescence analysis to assess the expression and localization of IL-4Rα (red) and lipid raft marker Flotillin-1 (green) in BV-2 and HMC3 cells treated with DMSO, Cho, or ATO plus Cho (scale bar, 60 μm).

**Figure S20**

**
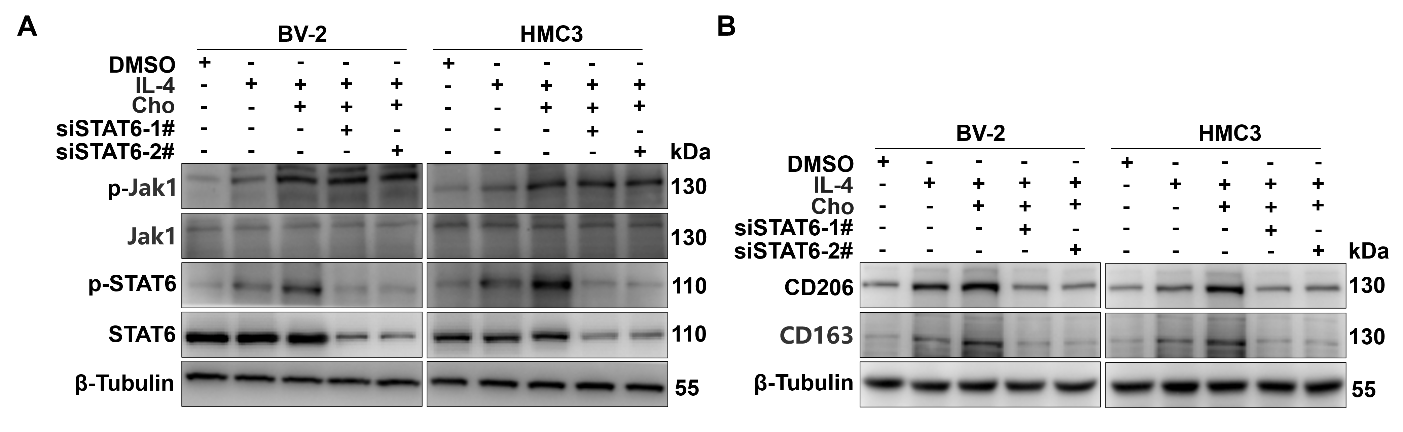
**

**Figure S20.** STAT6 knockdown further clarified the causal role of the JAK1/STAT6 axis in cholesterol-induced M2 microglial polarization. A, B) Western blot analyses showing the effects of STAT6 knockdown (siSTAT6-1# and siSTAT6-2#) expression on downstream signaling (A) and M2 marker (B) expression in microglial cells.

**Figure S21**

**
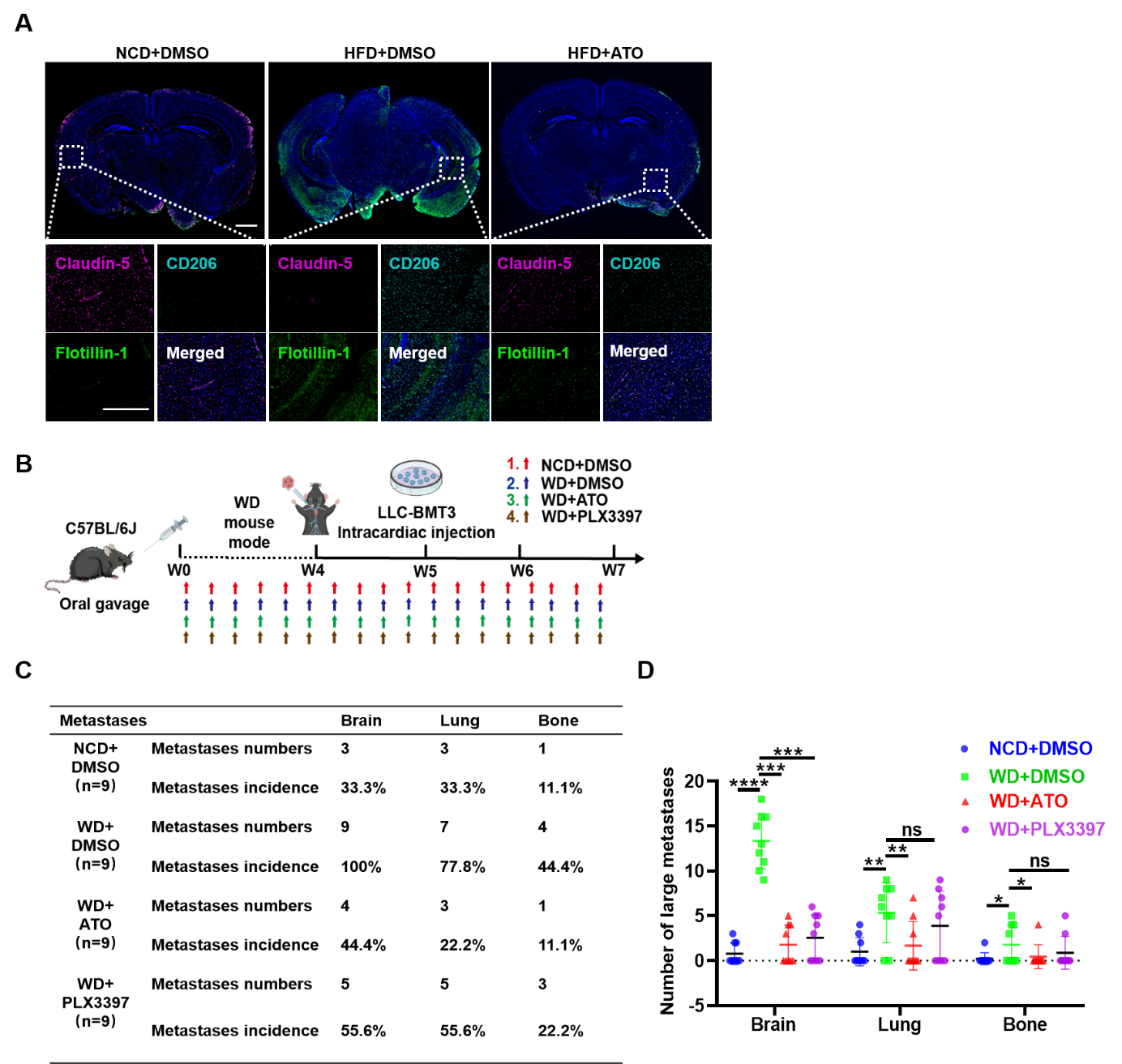
**

**Figure S21.** Hypercholesterolemia remodels the brain metastasis by enhancing lipid-raft abundance, promoting M2 microglial accumulation, and impairing BBB integrity. A) Brain sections were obtained from C57BL/6J male mice fed a NCD, HFD, or HFD with oral ATO (10 mg/kg/day) for 12 weeks. Expression levels of Claudin-5 (pink, BBB integrity marker)， Flotillin-1 (green, lipid-raft marker) and CD206 (cyan, M2 marker) was assessed by immunofluorescence staining (scale bar, 1 mm). B) Schematic diagram illustrating that C57BL/6J mice male mice (n = 9) received NCD, WD, WD with daily oral ATO (10 mg/kg) or WD with daily PLX3397 (40 mg/kg). At week 6, LLC-BMT3 cells were administered via intracardiac injection. C) Quantification of the percentages of mice that developed brain, lung and bone metastases after LLC-BMT3 cells were injected via intracardiac injection. D) Quantification of metastatic nodule numbers in each organ. **P* < 0.05, ***P* < 0.01, ****P* < 0.001, ****P < 0.0001, ns, not significant (one-way ANOVA).

**Figure S22**

**
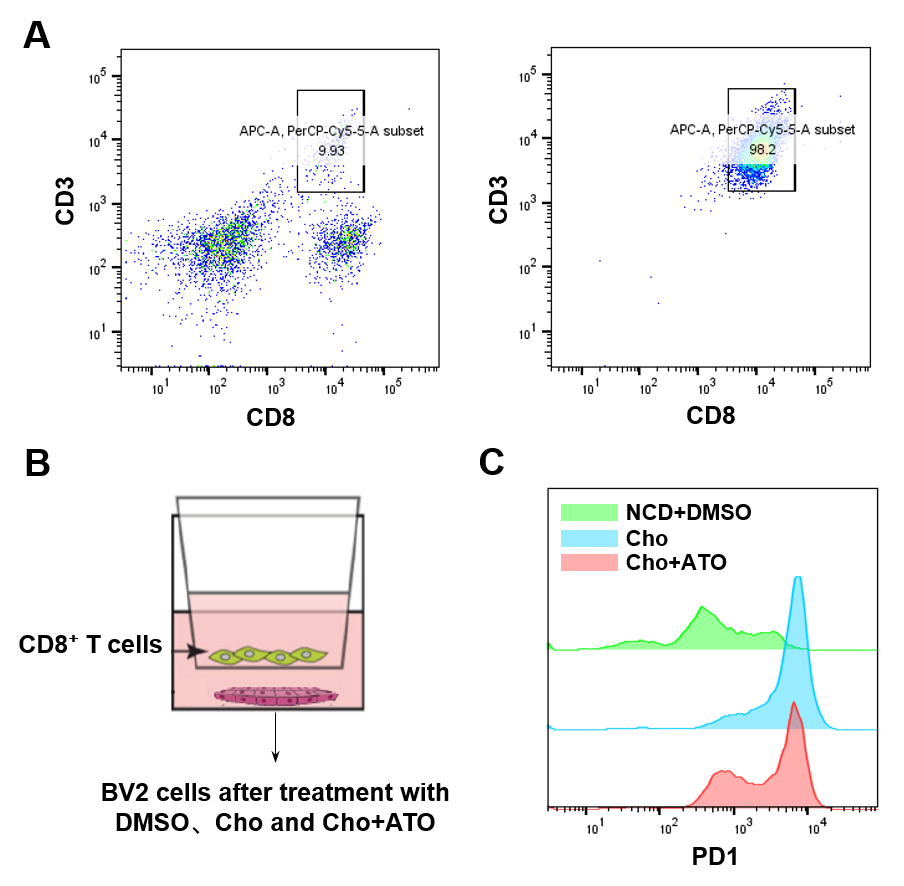
**

**Figure S22.** Cholesterol-activated microglia suppress CD8⁺ T-cell function via PD-1 upregulation. A) Flow-cytometry gating strategy for purification of CD8⁺ T cells. Representative plots show the sequential CD3⁺ and CD8⁺ gating steps used to isolate CD8⁺ T-cell populations. B) Schematic illustration of the transwell-based microglia–T-cell co-culture system. BV-2 microglia were pre-treated with DMSO, Cho, or Cho+ ATO for 48 h prior to co-culture with purified CD8⁺ T cells for 72 h. C) Flow-cytometry histograms showing PD-1 expression on CD8⁺ T cells following co-culture with microglia under the indicated treatments.

**Figure S23**

**
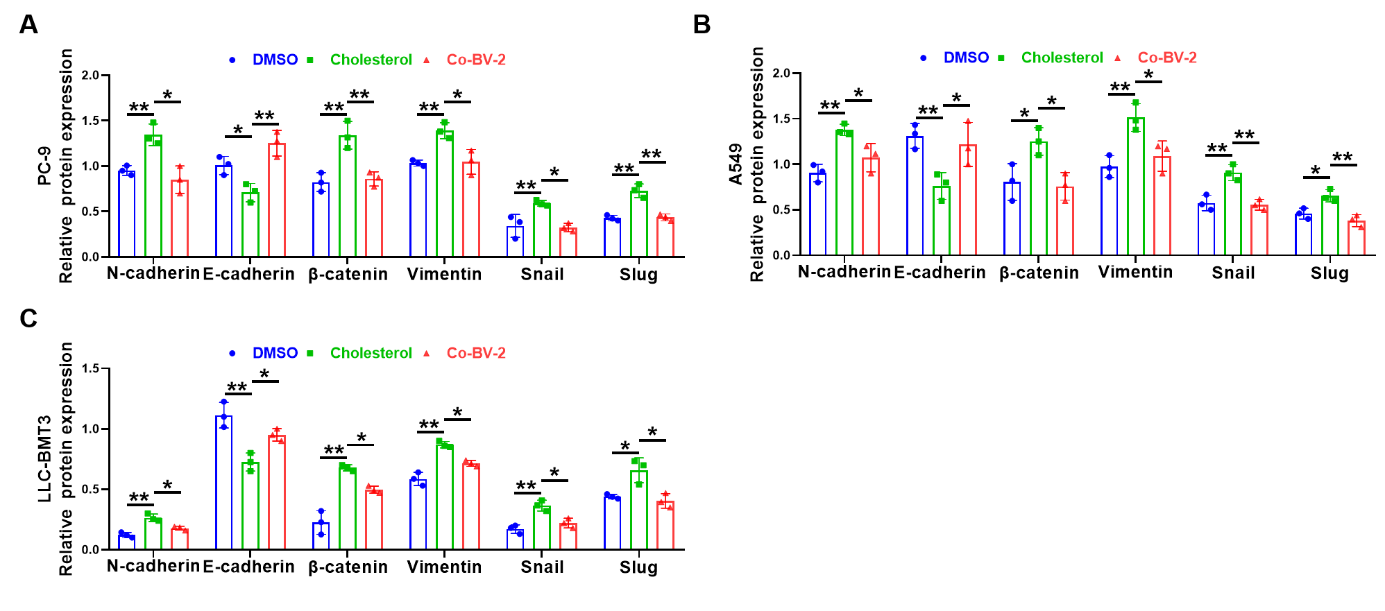
**

**Figure S23.** Cholesterol-treated microglia upregulated MET-associated markers in A549, PC-9 and LLC-BMT3 cells. A-C) EMT-related protein expression (β-catenin, N-cadherin, E-cadherin, Snail, Slug, Vimentin, and β-Tubulin) of A549 (A), PC-9 (B), and LLC (C) cells treated under the same conditions (DMSO, Cho , or supernatant from BV-2 cells.) were subjected to western blotting to quantitatively analyze the expression and β-Tubulin served as a loading control. **P* < 0.05, ***P* < 0.01 (one-way ANOVA).

**Figure S24**

**
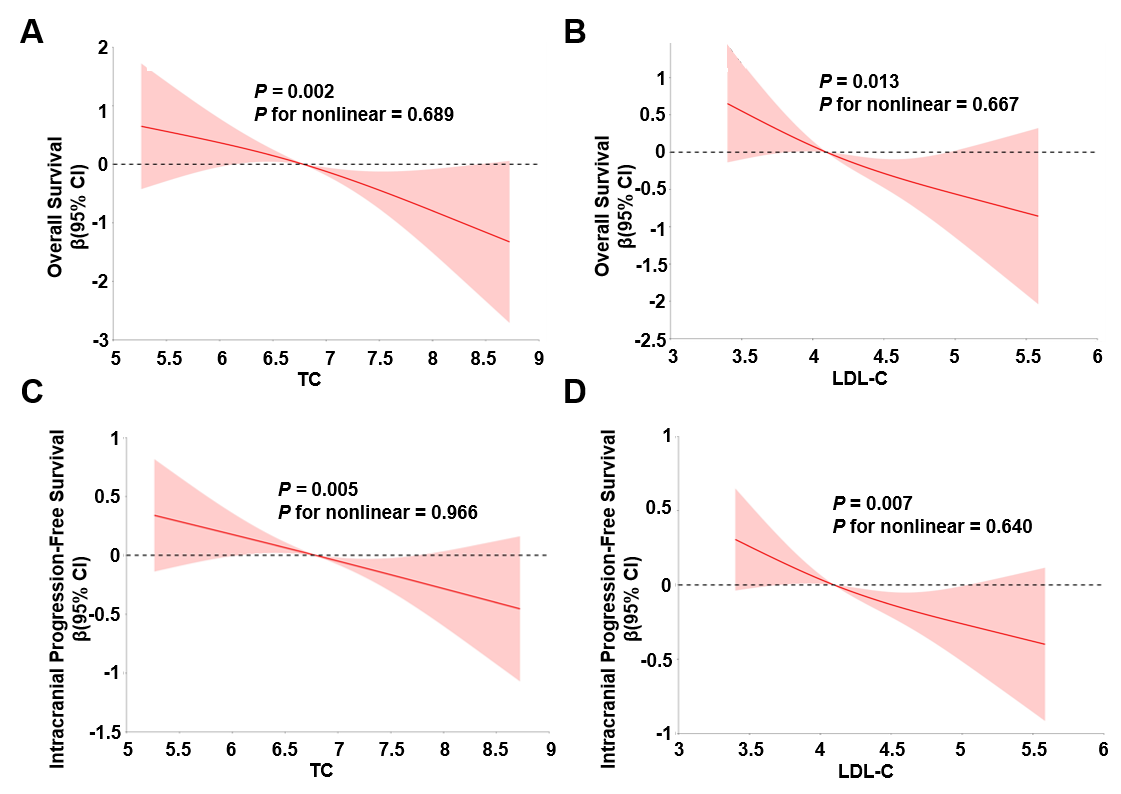
**

**Figure S24.** Clinical correlation of cholesterol with LUAD-BM. A-B) Restricted Cubic Splines for the association of the Interval from OS with TC (A) and LDL-C (B). C-D) Restricted Cubic Splines for the association of IPFS with TC (C) and LDL-C (D).

**Figure S25**

**
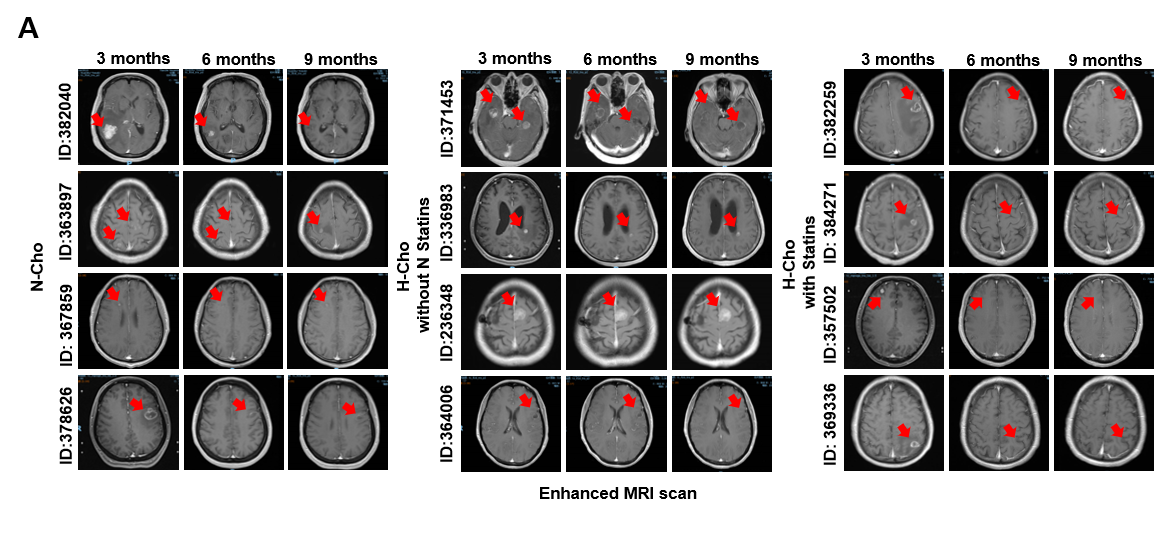
**

**Figure S25.** Representative contrast-enhanced brain MRI scans from LUAD-BM patients across different cholesterol and treatment groups. A) Representative intracranial MRI images of LUAD-BM cases from different patient groups during post-treatment follow-up.

**Figure S26**

**
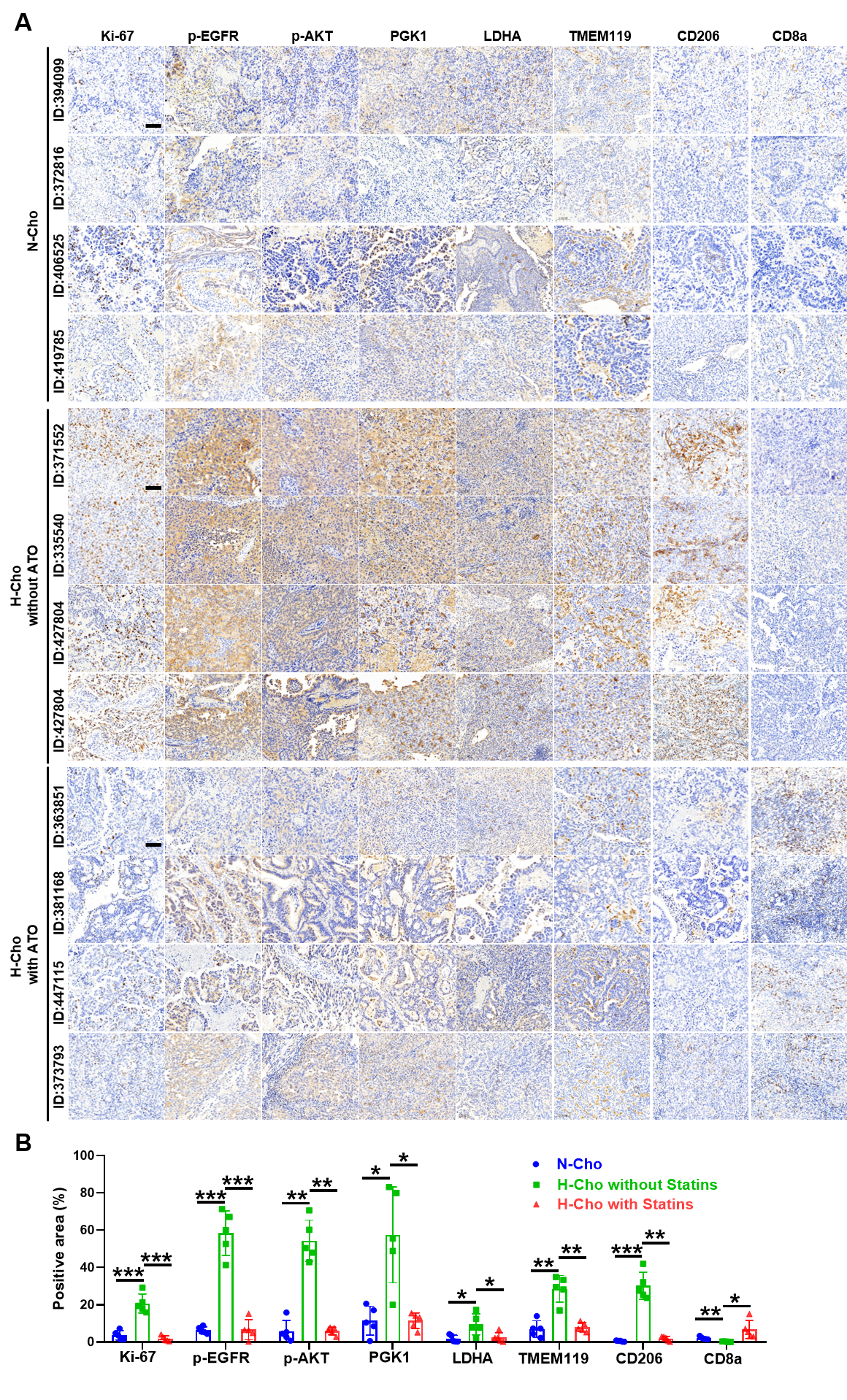
**

**Figure S26.** Clinical pathological analysis reveals cholesterol-associated pro-tumor and immunosuppressive signatures, mitigated by statins. A-B) Representative images of FFPE sections of primary tumor tissues in different groups for IHC staining of Ki67, p-EGFR, p-AKT, PGK1, LDHA, Iba1, CD206 and CD8a (Scale bar, 60 μm) (A) and quantitative analysis of IHC positive area. **P* < 0.05, ***P* < 0.01, ****P* < 0.001 (one-way ANOVA) (B).
