# Supplementary material for: Cholesterol Promotes Lung Adenocarcinoma Brain Metastasis by Stabilizing EGFR Protein to Drive EMT, Metabolic Reprogramming, and Premetastatic Niche Formation
Source: Adv Sci (Weinh). 2026 Jan 21;13(17):e73843. doi: 10.1002/advs.73843 (PMC13042990; doi:10.1002/advs.73843)
Supplement: Supplementary file 3 — Supporting File 3: advs73843‐sup‐0003‐TableS5‐S7.docx. [file ADVS-13-e73843-s001.docx]

### Table S5. Prognostic factors for the interval from primary diagnosis to brain metastasis of LUAD-BM patients

| Variables | Univariate analysis | | | | |  | multivariate analysis | | | | |
| --- | --- | --- | --- | --- | --- | --- | --- | --- | --- | --- | --- |
|  | β | S.E | Z | *P* | HR (95%CI) |  | β | S.E | Z | *P* | HR (95%CI) |
| **Groups** |  |  |  |  |  |  |  |  |  |  |  |
| N-Cho |  |  |  |  | 1.00 (Reference) |  |  |  |  |  | 1.00 (Reference) |
| H-Cho without Statins | 0.54 | 0.24 | 2.21 | 0.027 | 1.71 (1.06 ~ 2.74) |  | 0.70 | 0.28 | 2.49 | 0.013 | 2.01 (1.16 ~ 3.48) |
| H-Cho with Statins | -0.18 | 0.25 | -0.73 | 0.462 | 0.83 (0.51 ~ 1.36) |  | 0.04 | 0.27 | 0.14 | 0.885 | 1.04 (0.61 ~ 1.78) |
| **Age** | -0.01 | 0.01 | -1.15 | 0.249 | 0.99 (0.97 ~ 1.01) |  | -0.00 | 0.01 | -0.41 | 0.681 | 1.00 (0.97 ~ 1.02) |
| **Gender** |  |  |  |  |  |  |  |  |  |  |  |
| Male |  |  |  |  | 1.00 (Reference) |  |  |  |  |  | 1.00 (Reference) |
| Female | -0.04 | 0.20 | -0.19 | 0.846 | 0.96 (0.65 ~ 1.42) |  | 0.11 | 0.27 | 0.40 | 0.687 | 1.11 (0.66 ~ 1.88) |
| **BMI** | 0.02 | 0.04 | 0.37 | 0.708 | 1.02 (0.94 ~ 1.10) |  | 0.05 | 0.06 | 0.82 | 0.412 | 1.05 (0.94 ~ 1.17) |
| Mutation types |  |  |  |  |  |  |  |  |  |  |  |
| No positive |  |  |  |  | 1.00 (Reference) |  |  |  |  |  | 1.00 (Reference) |
| EGFR | -0.22 | 0.21 | -1.02 | 0.309 | 0.81 (0.53 ~ 1.22) |  | 0.49 | 0.67 | 0.73 | 0.464 | 1.63 (0.44 ~ 6.05) |
| Other mutation types | -0.18 | 0.32 | -0.56 | 0.572 | 0.83 (0.44 ~ 1.57) |  | 0.34 | 0.65 | 0.52 | 0.603 | 1.40 (0.39 ~ 5.05) |
| **Diabetes** |  |  |  |  |  |  |  |  |  |  |  |
| No |  |  |  |  | 1.00 (Reference) |  |  |  |  |  | 1.00 (Reference) |
| Yes | 0.14 | 0.30 | 0.47 | 0.637 | 1.15 (0.64 ~ 2.07) |  | 0.45 | 0.35 | 1.29 | 0.196 | 1.57 (0.79 ~ 3.09) |
| **Cardiovascular Disease** |  |  |  |  |  |  |  |  |  |  |  |
| No |  |  |  |  | 1.00 (Reference) |  |  |  |  |  | 1.00 (Reference) |
| Yes | -0.19 | 0.21 | -0.89 | 0.373 | 0.83 (0.54 ~ 1.26) |  | -0.34 | 0.29 | -1.17 | 0.241 | 0.71 (0.40 ~ 1.26) |
| **Line of therapy** |  |  |  |  |  |  |  |  |  |  |  |
| 1 |  |  |  |  | 1.00 (Reference) |  |  |  |  |  | 1.00 (Reference) |
| 2 | -0.11 | 0.24 | -0.45 | 0.649 | 0.90 (0.56 ~ 1.44) |  | 0.05 | 0.30 | 0.18 | 0.857 | 1.06 (0.59 ~ 1.90) |
| 3 | -0.53 | 0.29 | -1.84 | 0.066 | 0.59 (0.34 ~ 1.04) |  | -0.63 | 0.35 | -1.80 | 0.073 | 0.53 (0.27 ~ 1.06) |
| **TKI treatment** |  |  |  |  |  |  |  |  |  |  |  |
| No |  |  |  |  | 1.00 (Reference) |  |  |  |  |  | 1.00 (Reference) |
| Yes | -0.25 | 0.20 | -1.26 | 0.209 | 0.78 (0.53 ~ 1.15) |  | -0.51 | 0.66 | -0.78 | 0.438 | 0.60 (0.16 ~ 2.19) |
| **Smoking history** |  |  |  |  |  |  |  |  |  |  |  |
| No |  |  |  |  | 1.00 (Reference) |  |  |  |  |  | 1.00 (Reference) |
| Yes | 0.26 | 0.22 | 1.22 | 0.223 | 1.30 (0.85 ~ 1.98) |  | 0.29 | 0.32 | 0.89 | 0.372 | 1.33 (0.71 ~ 2.52) |
| **Alcohol use** |  |  |  |  |  |  |  |  |  |  |  |
| No |  |  |  |  | 1.00 (Reference) |  |  |  |  |  | 1.00 (Reference) |
| Yes | 0.58 | 0.31 | 1.87 | 0.061 | 1.79 (0.97 ~ 3.30) |  | 0.33 | 0.43 | 0.78 | 0.433 | 1.40 (0.61 ~ 3.21) |
| **Surgical intervention** |  |  |  |  |  |  |  |  |  |  |  |
| No |  |  |  |  | 1.00 (Reference) |  |  |  |  |  | 1.00 (Reference) |
| Yes | 0.28 | 0.27 | 1.05 | 0.295 | 1.32 (0.78 ~ 2.24) |  | 0.21 | 0.33 | 0.65 | 0.518 | 1.24 (0.65 ~ 2.35) |
| ECOG | 0.04 | 0.06 | 0.81 | 0.419 | 1.05 (0.94 ~ 1.16) |  | 0.06 | 0.06 | 0.97 | 0.331 | 1.06 (0.94 ~ 1.21) |
| **Metastasis Count** |  |  |  |  |  |  |  |  |  |  |  |
| No |  |  |  |  | 1.00 (Reference) |  |  |  |  |  | 1.00 (Reference) |
| Yes | 0.16 | 0.20 | 0.83 | 0.406 | 1.18 (0.80 ~ 1.73) |  | 0.12 | 0.23 | 0.54 | 0.591 | 1.13 (0.72 ~ 1.77) |
| HR: Hazard Ratio, CI: Confidence Interval | | | | | | | | | | | |

### Table S6. Prognostic factors for overall survival of LUAD-BM patients

| Variables | Univariate analysis | | | | |  | multivariate analysis | | | | |
| --- | --- | --- | --- | --- | --- | --- | --- | --- | --- | --- | --- |
|  | β | S.E | Z | *P* | HR (95%CI) |  | β | S.E | Z | *P* | HR (95%CI) |
| **Groups** |  |  |  |  |  |  |  |  |  |  |  |
| N-Cho |  |  |  |  | 1.00 (Reference) |  |  |  |  |  | 1.00 (Reference) |
| H-Cho without Statins | -0.28 | 0.26 | -1.11 | 0.267 | 0.75 (0.46 ~ 1.24) |  | -0.33 | 0.30 | -1.11 | 0.268 | 0.72 (0.40 ~ 1.29) |
| H-Cho with Statins | 0.41 | 0.22 | 1.84 | 0.066 | 1.51 (0.97 ~ 2.35) |  | 0.79 | 0.27 | 2.90 | 0.004 | 2.20 (1.29 ~ 3.74) |
| **Age** | 0.01 | 0.01 | 0.48 | 0.630 | 1.01 (0.98 ~ 1.03) |  | 0.01 | 0.01 | 0.47 | 0.641 | 1.01 (0.98 ~ 1.03) |
| **Gender** |  |  |  |  |  |  |  |  |  |  |  |
| Male |  |  |  |  | 1.00 (Reference) |  |  |  |  |  | 1.00 (Reference) |
| Female | -0.28 | 0.19 | -1.44 | 0.151 | 0.76 (0.52 ~ 1.11) |  | -0.38 | 0.26 | -1.48 | 0.138 | 0.68 (0.41 ~ 1.13) |
| **BMI** | -0.01 | 0.04 | -0.30 | 0.763 | 0.99 (0.92 ~ 1.07) |  | -0.02 | 0.04 | -0.53 | 0.596 | 0.98 (0.90 ~ 1.07) |
| Mutation types |  |  |  |  |  |  |  |  |  |  |  |
| No positive |  |  |  |  | 1.00 (Reference) |  |  |  |  |  | 1.00 (Reference) |
| EGFR | -0.26 | 0.21 | -1.25 | 0.213 | 0.77 (0.51 ~ 1.16) |  | -0.04 | 0.80 | -0.04 | 0.965 | 0.97 (0.20 ~ 4.66) |
| Other mutation types | -0.09 | 0.31 | -0.29 | 0.774 | 0.92 (0.50 ~ 1.67) |  | 0.28 | 0.71 | 0.39 | 0.697 | 1.32 (0.33 ~ 5.26) |
| **Diabetes** |  |  |  |  |  |  |  |  |  |  |  |
| No |  |  |  |  | 1.00 (Reference) |  |  |  |  |  | 1.00 (Reference) |
| Yes | 0.10 | 0.31 | 0.31 | 0.757 | 1.10 (0.60 ~ 2.02) |  | 0.31 | 0.36 | 0.87 | 0.384 | 1.36 (0.68 ~ 2.75) |
| **Cardiovascular Disease** |  |  |  |  |  |  |  |  |  |  |  |
| No |  |  |  |  | 1.00 (Reference) |  |  |  |  |  | 1.00 (Reference) |
| Yes | -0.12 | 0.22 | -0.53 | 0.595 | 0.89 (0.58 ~ 1.37) |  | 0.08 | 0.29 | 0.27 | 0.784 | 1.08 (0.61 ~ 1.92) |
| **Line of therapy** |  |  |  |  |  |  |  |  |  |  |  |
| 1 |  |  |  |  | 1.00 (Reference) |  |  |  |  |  | 1.00 (Reference) |
| 2 | -0.25 | 0.28 | -0.91 | 0.363 | 0.78 (0.45 ~ 1.34) |  | -0.08 | 0.35 | -0.22 | 0.825 | 0.93 (0.47 ~ 1.82) |
| 3 | -0.46 | 0.25 | -1.81 | 0.071 | 0.63 (0.39 ~ 1.04) |  | -0.46 | 0.31 | -1.46 | 0.144 | 0.63 (0.34 ~ 1.17) |
| **TKI treatment** |  |  |  |  |  |  |  |  |  |  |  |
| No |  |  |  |  | 1.00 (Reference) |  |  |  |  |  | 1.00 (Reference) |
| Yes | -0.24 | 0.19 | -1.21 | 0.225 | 0.79 (0.54 ~ 1.16) |  | -0.14 | 0.75 | -0.19 | 0.847 | 0.87 (0.20 ~ 3.75) |
| **Smoking history** |  |  |  |  |  |  |  |  |  |  |  |
| No |  |  |  |  | 1.00 (Reference) |  |  |  |  |  | 1.00 (Reference) |
| Yes | 0.34 | 0.21 | 1.63 | 0.104 | 1.41 (0.93 ~ 2.12) |  | 0.14 | 0.33 | 0.43 | 0.670 | 1.15 (0.60 ~ 2.21) |
| **Alcohol use** |  |  |  |  |  |  |  |  |  |  |  |
| No |  |  |  |  | 1.00 (Reference) |  |  |  |  |  | 1.00 (Reference) |
| Yes | 0.39 | 0.26 | 1.48 | 0.138 | 1.48 (0.88 ~ 2.48) |  | -0.06 | 0.41 | -0.15 | 0.880 | 0.94 (0.42 ~ 2.11) |
| **Surgical intervention** |  |  |  |  |  |  |  |  |  |  |  |
| No |  |  |  |  | 1.00 (Reference) |  |  |  |  |  | 1.00 (Reference) |
| Yes | -0.01 | 0.26 | -0.04 | 0.971 | 0.99 (0.59 ~ 1.65) |  | -0.09 | 0.32 | -0.27 | 0.788 | 0.92 (0.49 ~ 1.71) |
| ECOG |  | 0.00 | NA |  | NA (NA ~ NA) |  |  | 0.00 | NA |  | NA (NA ~ NA) |
| **Metastasis Count** | -0.07 | 0.19 | -0.37 | 0.714 | 0.93 (0.64 ~ 1.36) |  | -0.10 | 0.23 | -0.42 | 0.675 | 0.91 (0.58 ~ 1.42) |
| No |  |  |  |  | 1.00 (Reference) |  |  |  |  |  | 1.00 (Reference) |
| Yes | -0.03 | 0.20 | -0.13 | 0.895 | 0.97 (0.66 ~ 1.43) |  | 0.07 | 0.25 | 0.27 | 0.784 | 1.07 (0.66 ~ 1.74) |
| HR: Hazard Ratio, CI: Confidence Interval | | | | | | | | | | | |

### Table S7. Prognostic factors for intracranial progression-free survival of LUAD-BM patients

| Variables | Univariate analysis | | | | |  | multivariate analysis | | | | |
| --- | --- | --- | --- | --- | --- | --- | --- | --- | --- | --- | --- |
|  | β | S.E | Z | *P* | HR (95%CI) |  | β | S.E | Z | *P* | HR (95%CI) |
| **Groups** |  |  |  |  |  |  |  |  |  |  |  |
| N-Cho |  |  |  |  | 1.00 (Reference) |  |  |  |  |  | 1.00 (Reference) |
| H-Cho without Statins | -0.21 | 0.21 | -1.00 | 0.319 | 0.81 (0.54 ~ 1.22) |  | -0.37 | 0.24 | -1.57 | 0.117 | 0.69 (0.43 ~ 1.10) |
| H-Cho with Statins | 0.54 | 0.19 | 2.90 | 0.004 | 1.72 (1.19 ~ 2.49) |  | 0.56 | 0.21 | 2.73 | 0.006 | 1.75 (1.17 ~ 2.62) |
| **Age** | 0.01 | 0.01 | 0.69 | 0.490 | 1.01 (0.99 ~ 1.02) |  | -0.00 | 0.01 | -0.13 | 0.894 | 1.00 (0.98 ~ 1.02) |
| **Gender** |  |  |  |  |  |  |  |  |  |  |  |
| Male |  |  |  |  | 1.00 (Reference) |  |  |  |  |  | 1.00 (Reference) |
| Female | -0.24 | 0.16 | -1.51 | 0.131 | 0.79 (0.58 ~ 1.07) |  | -0.33 | 0.19 | -1.70 | 0.088 | 0.72 (0.49 ~ 1.05) |
| **BMI** | -0.02 | 0.03 | -0.78 | 0.433 | 0.98 (0.93 ~ 1.03) |  | -0.02 | 0.03 | -0.51 | 0.610 | 0.98 (0.92 ~ 1.05) |
| Mutation types |  |  |  |  |  |  |  |  |  |  |  |
| No positive |  |  |  |  | 1.00 (Reference) |  |  |  |  |  | 1.00 (Reference) |
| EGFR | -0.31 | 0.17 | -1.80 | 0.072 | 0.74 (0.53 ~ 1.03) |  | 0.04 | 0.50 | 0.08 | 0.934 | 1.04 (0.39 ~ 2.77) |
| Other mutation types | -0.52 | 0.25 | -2.09 | 0.037 | 0.60 (0.37 ~ 0.97) |  | -0.14 | 0.46 | -0.30 | 0.767 | 0.87 (0.35 ~ 2.16) |
| **Diabetes** |  |  |  |  |  |  |  |  |  |  |  |
| No |  |  |  |  | 1.00 (Reference) |  |  |  |  |  | 1.00 (Reference) |
| Yes | -0.09 | 0.23 | -0.39 | 0.699 | 0.91 (0.58 ~ 1.44) |  | -0.06 | 0.27 | -0.20 | 0.838 | 0.95 (0.55 ~ 1.61) |
| **Cardiovascular Disease** |  |  |  |  |  |  |  |  |  |  |  |
| No |  |  |  |  | 1.00 (Reference) |  |  |  |  |  | 1.00 (Reference) |
| Yes | 0.24 | 0.17 | 1.39 | 0.165 | 1.27 (0.91 ~ 1.77) |  | 0.46 | 0.20 | 2.34 | 0.019 | 1.59 (1.08 ~ 2.33) |
| **Line of therapy** |  |  |  |  |  |  |  |  |  |  |  |
| 1 |  |  |  |  | 1.00 (Reference) |  |  |  |  |  | 1.00 (Reference) |
| 2 | -0.15 | 0.22 | -0.68 | 0.498 | 0.86 (0.56 ~ 1.33) |  | 0.09 | 0.26 | 0.35 | 0.728 | 1.10 (0.65 ~ 1.84) |
| 3 | -0.13 | 0.21 | -0.63 | 0.531 | 0.88 (0.58 ~ 1.32) |  | -0.04 | 0.24 | -0.18 | 0.856 | 0.96 (0.60 ~ 1.53) |
| **TKI treatment** |  |  |  |  |  |  |  |  |  |  |  |
| No |  |  |  |  | 1.00 (Reference) |  |  |  |  |  | 1.00 (Reference) |
| Yes | -0.37 | 0.16 | -2.32 | 0.020 | 0.69 (0.50 ~ 0.94) |  | -0.46 | 0.48 | -0.97 | 0.335 | 0.63 (0.25 ~ 1.61) |
| **Smoking history** |  |  |  |  |  |  |  |  |  |  |  |
| No |  |  |  |  | 1.00 (Reference) |  |  |  |  |  | 1.00 (Reference) |
| Yes | 0.22 | 0.17 | 1.30 | 0.192 | 1.25 (0.89 ~ 1.74) |  | 0.03 | 0.25 | 0.10 | 0.919 | 1.03 (0.63 ~ 1.66) |
| **Alcohol use** |  |  |  |  |  |  |  |  |  |  |  |
| No |  |  |  |  | 1.00 (Reference) |  |  |  |  |  | 1.00 (Reference) |
| Yes | 0.25 | 0.22 | 1.14 | 0.253 | 1.28 (0.84 ~ 1.95) |  | -0.25 | 0.31 | -0.80 | 0.423 | 0.78 (0.42 ~ 1.44) |
| **Surgical intervention** |  |  |  |  |  |  |  |  |  |  |  |
| No |  |  |  |  | 1.00 (Reference) |  |  |  |  |  | 1.00 (Reference) |
| Yes | -0.25 | 0.20 | -1.23 | 0.217 | 0.78 (0.53 ~ 1.16) |  | -0.38 | 0.22 | -1.74 | 0.083 | 0.68 (0.44 ~ 1.05) |
| ECOG | 0.15 | 0.05 | 3.10 | 0.002 | 1.16 (1.06 ~ 1.28) |  | 0.14 | 0.05 | 2.68 | 0.007 | 1.15 (1.04 ~ 1.28) |
| **Metastasis Count** |  |  |  |  |  |  |  |  |  |  |  |
| No |  |  |  |  | 1.00 (Reference) |  |  |  |  |  | 1.00 (Reference) |
| Yes | -0.01 | 0.16 | -0.05 | 0.964 | 0.99 (0.73 ~ 1.35) |  | 0.07 | 0.18 | 0.40 | 0.692 | 1.08 (0.75 ~ 1.54) |

HR: Hazard Ratio, CI: Confidence Interval
